# Safety and efficacy of jaktinib in the treatment of Janus kinase inhibitor‐naïve patients with myelofibrosis: Results of a phase II trial

**DOI:** 10.1002/ajh.26709

**Published:** 2022-10-04

**Authors:** Yi Zhang, Hu Zhou, Zhongxing Jiang, Dengshu Wu, Junling Zhuang, Wei Li, Qian Jiang, Xiuli Wang, Jinwen Huang, Huanling Zhu, Linhua Yang, Xin Du, Fei Li, Ruixiang Xia, Feng Zhang, Jianda Hu, Yan Li, Yu Hu, Jing Liu, Chenghao Jin, Kai Sun, Zeping Zhou, Liqing Wu, Wenjuan Yu, Jie Jin

**Affiliations:** ^1^ Department of Hematology The First Affiliated Hospital, Zhejiang University School of Medicine Hangzhou P.R. China; ^2^ Zhejiang Provincial Key Laboratory of Hematopoietic Malignancy Zhejiang University Hangzhou P.R. China; ^3^ Zhejiang Provincial Clinical Research Center for Hematological Disorders Hangzhou P.R. China; ^4^ Zhejiang University Cancer Center Hangzhou P.R. China; ^5^ Department of Hematology The Affiliated Cancer Hospital of Zhengzhou University, Henan Cancer Hospital Zhengzhou P.R. China; ^6^ Department of Hematology The First Affiliated Hospital of Zhengzhou University Zhengzhou P.R. China; ^7^ Department of Hematology Xiangya Hospital, Central South University Changsha P.R. China; ^8^ Department of Hematology Peking Union Medical College Hospital (Dongdan Campus) Beijing P.R. China; ^9^ Department of Hematology, Cancer Center The First Hospital of Jilin University Changchun P.R. China; ^10^ Peking University Institute of Hematology, National Clinical Research Center for Hematologic Disease Beijing Key Laboratory of Hematopoietic Stem Cell Beijing P.R. China; ^11^ Department of Oncology Hematology The Second Hospital of Jilin University Changchun P.R. China; ^12^ Department of Hematology Sir Run Run Shaw Hospital, Zhejiang University School of Medicine Hangzhou P.R. China; ^13^ Department of Hematology West China Hospital, Sichuan University Chengdu P.R. China; ^14^ Department of Hematology The Second Hospital of Shanxi Medical University Taiyuan P.R. China; ^15^ Department of Hematology Guangdong Provincial People's Hospital, Guangdong Academy of Medical Sciences Guangzhou P.R. China; ^16^ Department of Hematology The First Affiliated Hospital of Nanchang University Nanchang P.R. China; ^17^ Department of Hematology The First Affiliated Hospital of Anhui Medical University Hefei P.R. China; ^18^ Department of Hematology The First Affiliated Hospital of Bengbu Medical College Bengbu P.R. China; ^19^ Fujian Institute of Hematology Fujian Medical University Union Hospital Fuzhou P.R. China; ^20^ Department of Hematopathology The First Hospital of China Medical University Shenyang P.R. China; ^21^ Department of Hematology, Union Hospital, Tongji Medical College Huazhong University of Science and Technology Wuhan P.R. China; ^22^ Department of Hematology Third Xiangya Hospital of Central South University Changsha P.R. China; ^23^ Department of Hematology Jiangxi Provincial People's Hospital Nanchang P.R. China; ^24^ Department of Hematology Henan Provincial People's Hospital Zhengzhou P.R. China; ^25^ Department of Hematology The Second Affiliated Hospital of Kunming Medical University Kunming P.R. China; ^26^ Suzhou Zelgen Biopharmaceuticals Co, Ltd Suzhou P.R. China

## Abstract

Myelofibrosis (MF) is associated with several constitutional symptoms. Currently, there are few therapeutic options for MF. Jaktinib, a novel, small‐molecule inhibitor of JAK, is currently being studied for its potential to treat MF. This phase 2 trial investigated efficacy and safety of jaktinib in the treatment of MF patients. The primary end point was the proportion of patients with ≥35% reduction in spleen volume (SVR35, proportion of patients with ≥35% reduction in spleen volume) at week 24. The secondary end points included improvement of anemia, rates of symptom response, and safety profile. Between January 8, 2019 and August 29, 2020, 118 patients were recruited and treated with either jaktinib 100 mg BID or 200 mg QD. At week 24, 54.8% (34/62) of patients in the 100 mg BID group and 31.3% (15/48) in the 200 mg QD group achieved SVR35 (*p* = .0199). Jaktinib treatment increased hemoglobin level to ≥20 g/L in 35.6% (21/59) of patients with hemoglobin ≤100 g/L at baseline. The proportion of patients who achieved a ≥50% improvement in total symptom score at week 24 was 69.6% (39/56) in the BID group and 57.5% (23/40) in the QD group. The most common ≥ grade 3 hematological treatment‐emergent adverse events (TEAEs; ≥ 10%) were anemia (100 mg BID: 24.2%, 200 mg QD: 28.8%), thrombocytopenia (16.7%, 11.5%), and neutropenia (3.0%, 11.5%). All non‐hematological TEAEs were mild. These results indicate that jaktinib can shrink the spleen, improve anemia, and other clinical symptoms with good tolerability.

## 1 INTRODUCTION

Myelofibrosis (MF) is a myeloproliferative neoplasm (MPN) that manifests as primary myelofibrosis (PMF) or secondary MF. Secondary MF further classified as post‐polycythemia vera MF (post‐PV MF) and post‐essential thrombocythemia MF (post‐ET MF). Clinically, the main features of MF are bone marrow fibrous tissue hyperplasia, extramedullary hematopoiesis, anemia, hepatosplenomegaly, constitutional symptoms, and progression to leukemia.^1^ The commonly used prognostic scoring systems for primary MF are the International Prognostic Scoring System (IPSS), Dynamic International Prognostic Scoring System (DIPSS), and DIPSS‐plus.^2^ In recent years, the Mutation‐Enhanced International Prognostic Score System (MIPSS70), MIPSS70‐plus, and Genetically Inspired Prognostic Scoring System (GIPSS) have been established to determine the prognosis of primary MF, and the Mysec Prognostic Model (MYSEC‐PM) for secondary MF. Currently, JAK inhibitors are the first‐line treatment for MF with symptomatic splenomegaly. Ruxolitinib is a highly selective and orally bioavailable inhibitor of JAK1 and JAK2.^3^ Phase III clinical trials (NCT00952289 and NCT00934544) have affirmed the safety and efficacy of ruxolitinib and also established its therapeutic benefit in adult MF.^4^, ^5^ Fedratinib, a highly selective JAK2 inhibitor, was found to be effective in phase II and phase III (JAKARTA I and JAKARTA II) clinical trials. Consequently, it was the second drug for MF approved by the USA Food and Drug Administration (US‐FDA).^6^, ^7^ Pacritinib is the first and only JAK1‐sparing inhibitor of JAK2 and IRAK1 which was recently approval by US‐FDA for the management of cytopenic MF. Momelotinib has undergone several clinical trials, with two III phase trials competed, and some III phase trails on going.^8^, ^9^ In China, there are no effective drugs for the management of symptoms of MF patients.

Jaktinib is a deuterated compound of momelotinib that has been found to be a small‐molecule inhibitor of JAK. Currently, it is being studied due to its potential to treat MF patients. Results from preclinical pharmacokinetic studies and the phase I trial in healthy Chinese volunteers revealed that jaktinib has a stable pharmacokinetic profile.^10^ The pharmacological mechanism of jaktinib is similar to that of momelotinib. Compared with JAK1, jaktinib has higher inhibitory effect on JAK2 or TYK2. It can also inhibit ACVR1 like momelotinib does. To further evaluate the efficacy and safety of jaktinib, we conducted a two‐arm, open‐label, multicenter, randomized, two‐stage phase II study in 118 patients with intermediate‐ or high‐risk MF.

## 2 METHODS

### 2.1 Eligibility criteria

Patients were considered eligible for the study if they met the following criteria: 18 years of age or older with a diagnosis of primary MF, post‐PV MF, or post‐ET MF according to the 2016 World Health Organization (WHO) and modified International Working Group for Myelofibrosis Research and Treatment criteria; a life expectancy of 6 months or longer; a Dynamic International Prognostic Scoring System plus (DIPSS‐plus) score of 3 to 4 (intermediate‐2 risk), 5 to 6 (high‐risk), or 1 to 2 (intermediate‐1 risk) with symptomatic splenomegaly; Eastern Cooperative Oncology Group (ECOG) performance status of 0 to 2; palpable splenomegaly (≥5 cm below the left costal margin); no JAK inhibitor exposure in the past; < 10% peripheral blood blasts; and a platelet count ≥75 × 10^9^/L. Patients were excluded if they had received any anti‐MF treatment within 2 weeks or six half‐lives (whichever is longer) prior to the first dose, and had a history of radiotherapy to the spleen within 12 months before screening, malignant tumors in the past 5 years, any significant clinical and laboratory abnormalities or serious diseases that the investigator considered would affect the safety assessment. Details of the inclusion and exclusion criteria, information about the trial design, and the statistical analysis plan are described in Supplementary 1 (Trial protocol). All patients provided written informed consent before undergoing any procedures.

### 2.2 Study design

This two‐arm, open‐label, multicenter, randomized phase II clinical trial was conducted at 22 sites in China. This study was sponsored and designed by Suzhou Zelgen Biopharmaceuticals Co., Ltd. It was approved by the institutional review board/independent ethics committee at each participating site and conducted in accordance with the International Conference on Harmonization guidelines for Good Clinical Practice.

In the first stage of the study, 104 patients from 21 sites were randomly assigned to two groups using an interactive web response system at a 1:1 ratio: the open‐label jaktinib 100 mg BID group and 200 mg QD group. A medium‐term analysis showed that jaktinib at a dose of 100 mg BID showed good efficacy. Therefore, 14 more participants were enrolled in the 100 mg BID group in the second stage of the study. Treatment assignments were known to all members of the research team and the patients. Eligible participants were scheduled to undergo at least four treatment cycles for 24 weeks (6 weeks per cycle). Clinic visits were performed every 1 to 2 weeks during the first cycle, every 3 weeks during the 2nd to 4th cycles, every 6 weeks during 5th‐8th cycles, and every 12 weeks after 48 weeks. Response was assessed once per cycle whereas safety was assessed during each visit. Spleen assessment was performed every 12 weeks by computed tomography (CT) or magnetic resonance imaging (MRI). Myeloproliferative Neoplasm Symptom Assessment Form Total Symptom Score (MPN‐SAF TSS) assessment was performed every 6 weeks before 48 weeks and every 12 weeks after 48 weeks of treatment. The dose of jaktinib was adjusted during treatment according to the platelet count and neutrophil count (CTCAE 4.03) (Supplementary 2, Table S1. Dose adjustment). Patients in all arms were treated until disease progression or unacceptable toxicity.

### 2.3 Study end points

The primary end point was a reduction of at least 35% in spleen volume (SVR35) at week 24 compared with baseline. The SVR35 was evaluated using MRI or CT scan by a blinded central reader. Main secondary end points were the proportion of participants who had SVR35 during the study period, time from the first dose to SVR35, duration of spleen response (duration from the time when SVR35 was achieved until the time when the spleen volume increased by 25% or more from the nadir), proportion of transfusion‐dependent patients who converted to transfusion independent (transfusion dependent: requires red blood cell (RBC) transfusion of at least 2U within 30 days prior to administration of the drug, converted to transfusion independent: no RBC transfusion for more than 12 weeks and hemoglobin ≥85 g/L during the treatment phase), the proportion of transfusion‐independent patients whose hemoglobin was ≤100 g/L at baseline and increased by ≥20 g/L after treatment, proportion of patients with at least 50% decrease in the times of RBC infusions (average times of blood transfusions per month after jaktinib treatment compared with the frequency of blood transfusions within 30 days prior to the first dose) during the entire treatment, proportion of patients whose MPN‐SAF TSS decreased by ≥50% at week 24, absolute and percentage decrease in MPN‐SAF TSS on prespecified visit, and safety. Adverse events (AEs) were graded according to the Common Toxicity Criteria for Adverse Events (CTCAE) version 4.03. Overall survival (OS) was defined as the time from the first dose to death or last known alive date.

### 2.4 Statistical analysis

A total of 100 patients were randomly assigned at a 1:1 ratio to the jaktinib 100 mg BID and jaktinib 200 mg QD in the first stage. With 50 patients in a treatment group, the half width of the 95% confidence interval (CI) was not greater than 15%, assuming the proportion of patients who had SVR35 at 24 weeks was 48% and the drop‐out rate was below 10% based on published myelofibrosis studies on momelotinib and ruxolitinib. Having obtained good results at the dosage of 100 mg bid, we started the second stage of the study. To obtain a sample size that meets the requirements for a potential new drug application by China National Medical Products Administration, additional 36 patients were enrolled in the jaktinib 100 mg BID group in the second stage. This provided at least 80% probability to reject the null hypothesis that the SVR35 at 24 weeks was less than 23% at a significance level of two‐sided 0.05 even if the data of patients receiving jaktinib 100 mg BID in stage 1 were not taken into account assuming the SVR35 at week 24 was 48% for jaktinib and the drop‐out rate was 10%. During the study, the sponsor decided to initiate a randomized, active controlled phase III trial following an agreement with the regulatory agency. Therefore, participant recruitment at the second stage was terminated earlier. Finally, 66 patients treated with jaktinib 100 mg BID and 52 patients treated with jaktinib 200 mg QD were enrolled, providing an adequate statistical power based on the aforementioned assumptions, despite the early termination.

Drug efficacy was determined in all patients who received at least one dose of jaktinib in accordance with the intention‐to‐treat (ITT) principle. However, for variables which need to be compared with baseline data, like SVR35, patients with missing baseline were excluded from the analyses. For the primary end point, data of patients who did not undergo the assessment of spleen volume at week 24 were imputed using the last observation carried forward (LOCF) method. Therefore, 13 patients with at least one post‐baseline assessment were included to calculate the SVR35 by LOCF method. Sensitivity analyses for the primary end point included analysis of the per‐protocol analysis set (PPS), analysis using multiple imputation or observed case or non‐responder imputation to handle missing data, and analysis based on the spleen volume were evaluated by the investigator. For binary end points, the Clopper Pearson method was used to calculate the 95% CIs, and the Fisher's exact test was used for between‐group comparisons. The time‐to‐event end points including durability of splenic response, and OS were analyzed using the Kaplan–Meier method. Hazard ratios (HRs) and the corresponding 95% CIs were estimated using the Cox proportional hazards model. Comparison of data of between‐group treatments were performed using a two‐sided log‐rank test. For continuous end points, the values and changes from baseline were summarized for each treatment group, and no imputation was carried out for missing data. All statistical analyses were performed using SAS version 9.4. *p* < .05 was considered as significant (Supplementary 3, Table S2. Data set). The trial was registered with the ClinicalTrials.gov, registration number: NCT03886415.

## 3 RESULTS

**FIGURE 1:**
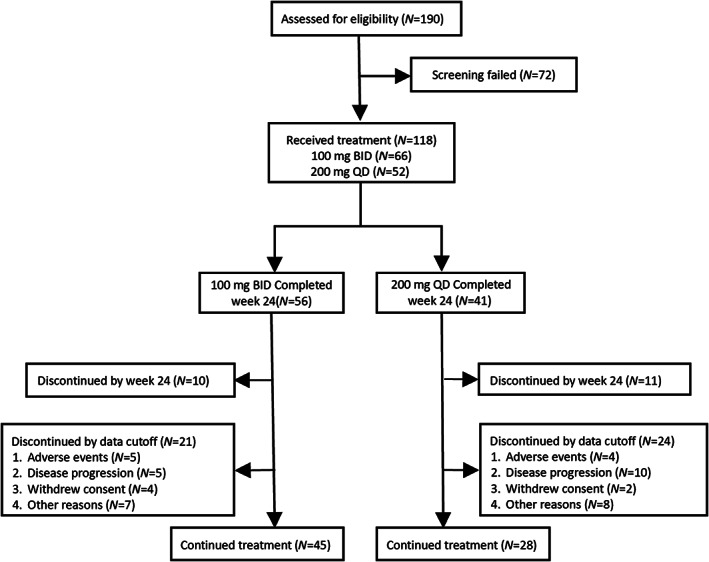
Patient disposition

In this multicenter trial, patients were enrolled from January 8, 2019 to August 29, 2020 at 22 study centers. A total of 190 patients were assessed for eligibility among which 118 were enrolled in the study: 66 were assigned to receive jaktinib 100 mg BID and 52 were assigned to 200 mg QD (Figure 1). There were 71 (60.2%) males and 47 (39.8%) females, with a median age of 60.5 (25 to 77) years; 89 (75.4%) of them had primary MF. Approximately, 70% of patients in both groups were classified as having intermediate‐2 according to DIPSS‐plus, and 59.3% and 7.6% of patients having intermediate‐2 and high risk according to DIPSS. Mutations of JAK2, CALR, and MPL were analyzed. Baseline characteristics were generally balanced between the two groups as shown in Table 1.

**TABLE 1:** Baseline characteristics

	100 mg BID (*N* = 66)	200 mg QD (*N* = 52)	Total (*N* = 118)
Age (years)	59.2 (9.19)	59.5 (10.40)	59.3 (9.70)
Sex			
Male	40 (60.6%)	31 (59.6%)	71 (60.2%)
Female	26 (39.4%)	21 (40.4%)	47 (39.8%)
Disease type			
PMF	48 (72.7%)	41 (78.8%)	89 (75.4%)
Post‐PV MF	12 (18.2%)	4 (7.7%)	16 (13.6%)
Post‐ET MF	6 (9.1%)	7 (13.5%)	13 (11.0%)
Disease duration (years)	1.8 (3.10)	3.1 (4.38)	2.3 (3.76)
MF pathology			
MF‐0	0	1 (1.9%)	1 (0.8%)
MF‐1	4 (6.1%)	0	4 (3.4%)
MF‐2	30 (45.5%)	14 (26.9%)	44 (37.3%)
MF‐3	24 (36.4%)	30 (57.7%)	54 (45.8%)
Unidentified	8 (12.1%)	7 (13.5%)	15 (12.7%)
DIPSS‐plus status			
Intermediate‐1	20 (30.3%)	16 (30.8%)	36 (30.5%)
Intermediate‐2	45 (68.2%)	36 (69.2%)	81 (68.6%)
High‐risk	1 (1.5%)	0	1 (0.8%)
DIPSS status			
Intermediate‐1	20 (30.3%)	19 (36.5%)	39 (33.1%)
Intermediate‐2	40 (60.6%)	30 (57.7%)	70 (59.3%)
High‐risk	6 (9.1%)	3 (5.8%)	9 (7.6%)
JAK2 mutational profile			
Wild type	19 (28.8%)	19 (36.5%)	38 (32.2%)
Mutant	47 (71.2%)	32 (61.5%)	79 (66.9%)
Missing	0	1 (1.9%)	1 (0.8%)
CALR mutational profile			
Wild type	54 (81.8%)	35 (67.3%)	89 (75.4%)
Mutant	11 (16.7%)	17 (32.7%)	28 (23.7%)
Missing	1 (1.5%)	0	1 (0.8%)
MPL mutational profile			
Wild type	59 (89.4%)	52 (100%)	111 (94.1%)
Mutant	6 (9.1%)	0	6 (5.1%)
Missing	1 (1.5%)	0	1 (0.8%)
High risk karyotype*	3 (4.5%)	2 (3.8%)	5 (4.2%)
WBC (10^9^/L)	17.453 (18.9397)	12.291 (8.7736)	15.178 (15.4745)
HGB (g/L)	107.51 (28.088)	101.86 (26.915)	105.02 (27.604)
PLT (10^9^/L)	311.36 (232.523)	315.92 (192.509)	313.37 (214.933)
ECOG performance status			
0	12 (18.2%)	8 (15.4%)	20 (16.9%)
1	53 (80.3%)	40 (76.9%)	93 (78.8%)
2	1 (1.5%)	4 (7.7%)	5 (4.2%)
RBC transfusion history^a^	5 (7.6%)	7 (13.5%)	12 (10.2%)
MFSAF‐TSS	29.6 (19.47)	20.9 (12.45)	25.8 (17.26)
Spleen volume, ml	1660.633 (758.9372)	1818.499 (1061.6298)	1730.201 (904.1398)

*Note*: Data were presented as mean (SD) or *n* (%). No significant differences between groups in any of the listed baseline characteristics. High risk karyotype* including +8, −7/7q‐, inv(17q), inv (3), 12p‐, 11q23 rearrangements and complex karyotypes.

Abbreviations: DIPSS‐plus, Dynamic International Prognostic Scoring System‐plus; ECOG, Eastern Cooperative Oncology Group; HGB, hemoglobin level; PLT, platelet count; TSS, total symptom score; WBC, white blood cell count.

^a^ RBC transfusion history: Number of people who received blood transfusions for MF‐related anemia prior to clinical trials.

**FIGURE 2:**
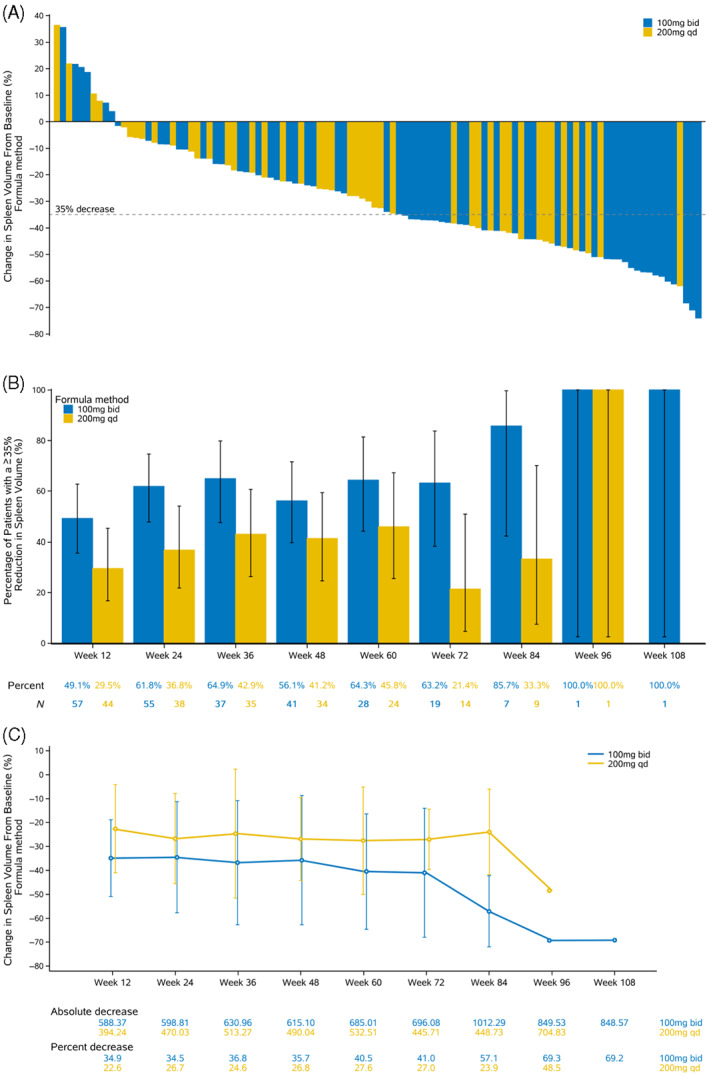
Change in spleen volume. Panel A shows the percent change in spleen volume from the baseline at week 24 or at the last evaluation before week 24. Eight patients with no post‐baseline assessment and four patients who received new antifibrotic drugs within 24 weeks (regarded as treatment failure) are not included. Panel B shows the proportion of patients whose spleen volume reduction is ≥35% from baseline as assessed using the MRI or CT over time. I bars denote 95% confidence interval. Panel C shows the mean absolute and percent changes in volume of spleen over time. I bars denote standard errors. In panels B and C only patients with evaluable results at the time point are included [Color figure can be viewed at wileyonlinelibrary.com]

The number of patients who completed week 24 were 56 in 100 mg BID and 41 in 200 mg QD respectively. Thirteen patients who did not undergo assessment for spleen volume at week 24 were imputed using the LOCF method. Overall, 62 patients who received jaktinib 100 mg BID and 48 patients who received 200 mg QD underwent spleen volume reduction assessment at week 24. In addition, eight of the 118 patients were excluded because did not undergo a single test on week 12 or 24. Most patients receiving jaktinib showed some degree of reduction in the spleen volume. The waterfall plot showing changes in spleen volume of individual patients at week 24 is in Figure 2A. Assessment by the independent review committee (IRC) showed that the proportion of patients who had SVR35 at 24 weeks was 44.5% (49/110) (95% CI: 35.1%–54.3%) in the overall population, 54.8% (34/62) (95% CI: 41.7%–67.5%) in the 100 mg BID group, and 31.3% (15/48) (95% CI: 18.7%–46.3%) in the 200 mg QD group (*p* = .0199). In addition, the reduction in spleen volume progressively increased with the continued treatment. Further analysis showed that the 100 mg BID had better results compared with 200 mg QD at every post‐treatment visit (Figure 2B,C). The benefits of 100 mg BID were also observed in multiple subgroups analyses (Supplementary 4, Figure S1. Subgroup analysis on the primary end point ‐SVR35 at week 24). Overall, the best splenic response rate during therapy was 62.1% (95% CI: 49.3%–73.8%) in the 100 mg BID group and 44.2% in the 200 mg QD group (95% CI: 30.5%–58.7%). The median time to achieve the first SVR35 was 5.5 months (95% CI: 2.8–5.6) in the 100 mg BID group and 11.0 months (95% CI: 5.4 to NE) in the 200 mg QD group (HR = 0.551, *p* = .0172). The median time of duration of SVR35 has not been reached. Among patients who achieved SVR35, 64.0% (95% CI: 48.1–76.2) showed a reduction in the spleen volume which persisted for 12 months or more.

In the ITT population, 35.6% (21/59) of the patients whose baseline hemoglobin was ≤100 g/L had a transfusion‐independent increase of ≥20 g/L in hemoglobin for 12 weeks at all visits after treatment. Of the six patients who were transfusion‐dependent at baseline, two became transfusion‐independent after treatment, all of whom were in the 200 mg QD group. In addition, five of seven (71.4%) patients had ≥50% decrease in the frequency of RBC infusions (Supplementary 5, Table S3. Anemia improvement).

A total of 56 patients in the BID group and 40 patients in the QD group were assessable for MPN‐SAF TSS at week 24. The jaktinib treatment improved the disease‐related symptoms of most patients (Supplementary 6, Figure S2. Change in MPN‐SAF TSS. A), and the improvement was persisted with the continued treatment (Supplementary 6, Figure S2B,C). The proportion of patients who achieved an improvement of ≥50% in the TSS at week 24 was 69.6% (95% CI: 55.9%–81.2%) in the BID group and 57.5% (95% CI: 40.9%–73.0%) in the QD group. Notably, there was no difference between the two groups. The absolute changes in MPN‐SAF TSS score at week 24 were 19.5 ± 16.87 (mean ± SD) and 10.9 ± 11.81, and the percent changes were −62.00 ± 49.341 (mean ± SD) and −42.01 ± 54.635 respectively.

Among the 118 patients in the ITT population, 117 had treatment‐emergent adverse events (TEAEs) of any grade. The most common grade 3 or 4 hematological TEAEs (≥ 10%) were anemia (100 mg BID: 24.2%, 200 mg QD: 28.8%), thrombocytopenia (16.7%, 11.5%), and neutropenia (3.0%, 11.5%). The most common non‐hematological TEAEs (≥ 10%; of any grade) were upper respiratory tract infection (24.2%, 30.8%), elevated creatinine (19.7%, 30.8%), elevated ALT (24.2%, 21.2%), and elevated bilirubin (24.2%, 13.5%), most of which were grade 1 or 2 (Table 2). Several drug‐related neuropathy diseases were reported, including six hypoesthesia (four in BID, two in QD), three peripheral neuropathy (all in the BID), and one sensory disturbance in BID group, most of them were grade 1 to 2. No patient was discontinued because of neuropathy diseases. Serious AEs occurred in 25 (37.9%) patients in the 100 mg BID group and 11 (21.2%) patients in the 200 mg QD group. The events were considered drug‐related in 11 patients in the BID group and two patients in the QD group. Serious AEs that occurred in ≥2 patients were infectious pneumonia (*N* = 8), anemia (*N* = 4), decreased platelet count (*N* = 3), intracerebral hemorrhage (*N* = 2), and acute heart failure (*N* = 2). The incidence of serious AEs associated with the drug was below 5%, except for pneumonia, which was 6.8%. A total of 64 (54.2%) of the 118 patients had their dose adjusted/interrupted, in most cases due to thrombocytopenia (31.4%), anemia (22.0%), and neutropenia (8.5%). Moreover, 12 (10.2%) patients discontinued the treatment because of AEs, 12.1% (8/66) in BID, and 7.7% (4/52) in QD.

**TABLE 2:** Treatment‐emergent adverse events (TEAEs) reported in ≥10% of patients in the ITT population

Term	100 mg bid (*N* = 66)						200 mg qd (*N* = 52)						Total (*N* = 118)					
	1	2	3	4	5	≥3	1	2	3	4	5	≥3	1	2	3	4	5	≥3
All AEs with an incidence ≥10%	13 (19.7%)	25 (37.9%)	23 (34.8%)	2 (3.0%)	2 (3.0%)	27 (40.9%)	13 (25.0%)	13 (25.0%)	21 (40.4%)	4 (7.7%)	0	25 (48.1%)	26 (22.0%)	38 (32.2%)	44 (37.3%)	6 (5.1%)	2 (1.7%)	52 (44.1%)
Increased blood creatinine	13 (19.7%)	0	0	0	0	0	15 (28.8%)	1 (1.9%)	0	0	0	0	28 (23.7%)	1 (0.8%)	0	0	0	0
Increased white blood cell count	15 (22.7%)	1 (1.5%)	0	0	0	0	10 (19.2%)	2 (3.8%)	0	0	0	0	25 (21.2%)	3 (2.5%)	0	0	0	0
Elevated alanine aminotransferase	12 (18.2%)	2 (3.0%)	2 (3.0%)	0	0	2 (3.0%)	10 (19.2%)	1 (1.9%)	0	0	0	0	22 (18.6%)	3 (2.5%)	2 (1.7%)	0	0	2 (1.7%)
Elevated aspartate aminotransferase	10 (15.2%)	2 (3.0%)	1 (1.5%)	0	0	1 (1.5%)	10 (19.2%)	0	0	0	0	0	20 (16.9%)	2 (1.7%)	1 (0.8%)	0	0	1 (0.8%)
Increased neutrophil count	11 (16.7%)	0	0	0	0	0	9 (17.3%)	0	0	0	0	0	20 (16.9%)	0	0	0	0	0
Thrombocytopenia	10 (15.2%)	8 (12.1%)	8 (12.1%)	3 (4.5%)	0	11 (16.7%)	9 (17.3%)	5 (9.6%)	5 (9.6%)	1 (1.9%)	0	6 (11.5%)	19 (16.1%)	13 (11.0%)	13 (11.0%)	4 (3.4%)	0	17 (14.4%)
Upper respiratory tract infection	10 (15.2%)	6 (9.1%)	0	0	0	0	8 (15.4%)	6 (11.5%)	2 (3.8%)	0	0	2 (3.8%)	18 (15.3%)	12 (10.2%)	2 (1.7%)	0	0	2 (1.7%)
Increased monocyte count	11 (16.7%)	0	0	0	0	0	7 (13.5%)	0	0	0	0	0	18 (15.3%)	0	0	0	0	0
Diarrhea	8 (12.1%)	3 (4.5%)	0	0	0	0	9 (17.3%)	0	0	0	0	0	17 (14.4%)	3 (2.5%)	0	0	0	0
Fatigue	8 (12.1%)	0	0	0	0	0	9 (17.3%)	1 (1.9%)	0	0	0	0	17 (14.4%)	1 (0.8%)	0	0	0	0
Thrombocytosis	12 (18.2%)	4 (6.1%)	0	0	0	0	3 (5.8%)	4 (7.7%)	0	0	0	0	15 (12.7%)	8 (6.8%)	0	0	0	0
Dizziness	6 (9.1%)	3 (4.5%)	0	0	0	0	9 (17.3%)	1 (1.9%)	0	0	0	0	15 (12.7%)	4 (3.4%)	0	0	0	0
Anemia	9 (13.6%)	8 (12.1%)	16 (24.2%)	0	0	16 (24.2%)	5 (9.6%)	8 (15.4%)	15 (28.8%)	0	0	15 (28.8%)	14 (11.9%)	16 (13.6%)	31 (26.3%)	0	0	31 (26.3%)
Pruritus	8 (12.1%)	0	0	0	0	0	6 (11.5%)	0	0	0	0	0	14 (11.9%)	0	0	0	0	0
Elevated blood bilirubin	9 (13.6%)	5 (7.6%)	2 (3.0%)	0	0	2 (3.0%)	4 (7.7%)	3 (5.8%)	0	0	0	0	13 (11.0%)	8 (6.8%)	2 (1.7%)	0	0	2 (1.7%)
Abdominal discomfort	8 (12.1%)	0	0	0	0	0	5 (9.6%)	0	0	0	0	0	13 (11.0%)	0	0	0	0	0
Increased γ‐glutamyltransferase	8 (12.1%)	3 (4.5%)	1 (1.5%)	0	0	1 (1.5%)	4 (7.7%)	0	1 (1.9%)	0	0	1 (1.9%)	12 (10.2%)	3 (2.5%)	2 (1.7%)	0	0	2 (1.7%)
Bone pain	6 (9.1%)	0	1 (1.5%)	0	0	1 (1.5%)	6 (11.5%)	0	0	0	0	0	12 (10.2%)	0	1 (0.8%)	0	0	1 (0.8%)
Stomach ache	5 (7.6%)	1 (1.5%)	0	0	0	0	6 (11.5%)	1 (1.9%)	0	0	0	0	11 (9.3%)	2 (1.7%)	0	0	0	0
Hyperuricemia	7 (10.6%)	0	1 (1.5%)	0	0	1 (1.5%)	4 (7.7%)	0	0	2 (3.8%)	0	2 (3.8%)	11 (9.3%)	0	1 (0.8%)	2 (1.7%)	0	3 (2.5%)
Headache	4 (6.1%)	5 (7.6%)	0	0	0	0	6 (11.5%)	2 (3.8%)	0	0	0	0	10 (8.5%)	7 (5.9%)	0	0	0	0
Proteinuria	4 (6.1%)	3 (4.5%)	0	0	0	0	4 (7.7%)	1 (1.9%)	0	0	0	0	8 (6.8%)	4 (3.4%)	0	0	0	0
Urinary tract infection	3 (4.5%)	5 (7.6%)	1 (1.5%)	0	0	1 (1.5%)	2 (3.8%)	2 (3.8%)	0	0	0	0	5 (4.2%)	7 (5.9%)	1 (0.8%)	0	0	1 (0.8%)
Hypertension	3 (4.5%)	5 (7.6%)	5 (7.6%)	0	0	5 (7.6%)	1 (1.9%)	5 (9.6%)	3 (5.8%)	0	0	3 (5.8%)	4 (3.4%)	10 (8.5%)	8 (6.8%)	0	0	8 (6.8%)
Decreased neutrophil count	2 (3.0%)	3 (4.5%)	2 (3.0%)	0	0	2 (3.0%)	2 (3.8%)	4 (7.7%)	5 (9.6%)	1 (1.9%)	0	6 (11.5%)	4 (3.4%)	7 (5.9%)	7 (5.9%)	1 (0.8%)	0	8 (6.8%)
Decreased white blood cell count	1 (1.5%)	5 (7.6%)	0	0	0	0	1 (1.9%)	6 (11.5%)	3 (5.8%)	1 (1.9%)	0	4 (7.7%)	2 (1.7%)	11 (9.3%)	3 (2.5%)	1 (0.8%)	0	4 (3.4%)
Pneumonia	0	2 (3.0%)	3 (4.5%)	0	2 (3.0%)	5 (7.6%)	1 (1.9%)	2 (3.8%)	3 (5.8%)	0	0	3 (5.8%)	1 (0.8%)	4 (3.4%)	6 (5.1%)	0	2 (1.7%)	8 (6.8%)

The last follow‐up date was February 2, 2021. The median follow‐up for the jaktinib 100 mg BID group was 14.2 (range 0.9–24.9) months and that for 200 mg QD group was 15.5 (range 1.6–23.0) months. During the trial, 15 of the 118 (12.7%) patients had an increase of ≥25% in the spleen volume from the lowest recorded volume. No patient progressed to leukemia. Notably, four (3.4%) patients died during the study. The causes of death were severe pneumonia (two in the BID group; one of the patients had concurrent heart failure) and intracerebral hemorrhage (one in each group). Except for one death from pneumonia, all other deaths were not related to jaktinib. The median overall survival time was not reached (Supplementary 7, Figure S3. Survival curves of OS).

## 4 DISCUSSION

The present randomized, phase II study evidently showed that jaktinib can effectively reduce size of spleen, improve symptoms in patients with MF, and cause mild drug‐related side effects. It was evident that the 100 mg dose of BID was significantly more effective spleen volume reduction as compared with that of than the 200 mg QD. This study also found an important clinical value of jaktinib in that it enhances the elimination or reduction of the need for blood transfusion in patients with severe anemia.

Jaktinib is a deuterated compound of momelotinib. According to the biotransformation pathway theory, it has been found that substituting the deuterium for hydrogen in jaktinib may slow down its metabolic rate (isotope effect). This is because carbon‐deuterium bonds are stronger than the carbon‐hydrogen bonds. Results of our previous research study showed that the ratio for the rate of elimination constant between jaktinib and momelotinib was 1: 1.44. In addition, the C_max_ and AUC_0−t_ values of jaktinib were 165% and 168% of that of momelotinib in rats after a single oral administration of jaktinib or momelotinib at a similar concentration of 3 mg/kg to rats. However, there is need for further investigation of the potential benefit of deuterium kinetic isotope effect on the disposition and safety of jaktinib in clinical studies.

Jaktinib can effectively shrink the spleen. In our study, it was found that 44.5% of all the patients (49 out of 110) and 54.8% in the 100 mg BID group reached the primary end point of 35% reduction in spleen volume at week 24 (SVR35 at week 24). It was found that the BID group had higher shrinkage of the spleen. This could be because of the higher drug valley concentration (C_min_) of the BID group, which could continuously inhibit the JAK/STAT pathway. In the COMFORT I and COMFORT II studies of ruxolitinib, SVR35 was reported at the 24th week in 41.9 and 32% of the patients respectively.^4^, ^5^ In the evaluation of ruxolitinib in Chinese patients with MF, it was found that 27% (19 out of 67) achieved 35% SVR at week 24.^11^ In JAKARTA I study,^7^ the SVR35 at week 24 was achieved by 36 and 40% of the patients in the fedratinib 400 mg and 500 mg groups, Furthermore, the SIMPLIFY‐1 study compared the efficacies of momelotinib and ruxolitinib in JAK‐naïve and IPSS intermediate‐ or in high‐risk patients with MF. It was noted that the SVR35 at week 24 was achieved in 26.5% and 29% of the patients respectively.^12^ In the study on SIMPLIFY‐2, it was found that 7% the patients previously treated with ruxolitinib were found to achieve SVR35 at week 24 after the change of ruxolitinib to momelotinib.^13^ Besides, the studies of PERSIST‐1(JAKi Naïve) and PERSIST‐2 carried out before the allowing the treatment with JAKi showed that between 19 and 18% of patients with MF achieved SVR35 at week 24 after treatment with pacritinib.^14^, ^15^ Nonetheless, the use of different prognostic scoring systems (IPSS /DIPSS/DIPSS‐plus), the difference in population, duration of treatment, phase of trial, and supportive treatment may also affect the primary end point.

Based on the results of the present study, it was evident that jaktinib was well‐tolerated. Anemia and thrombocytopenia are the frequent hematologic adverse events and the reasons for treatment discontinuation.^16^, ^17^ The incidences of grade 3 or higher grades of anemia and thrombocytopenia were 24.2% and 16.7% as well as 28.8% and 11.5% in the jaktinib 100 mg BID and 200 mg QD groups respectively. Despite the effective potential of ruxolitinib to reduce the size of spleen, it has been previously found that 75% of patients experience anemia, whereas 25% of the patients would develop severe anemia.^18^, ^19^ In addition, it has been found that grade 3 or higher anemia and thrombocytopenia are found in the COMFORT I (45.2% and 42% respectively). However, although the side effects of fedratinib are mild and can be tolerated, the Wernicke encephalopathy needs special attention.^6^, ^7^ An ongoing clinical trial is investigating whether momelotinib can improve anemia (MOMENTUM trial, NCT03165734: Momelotinib vs. Danazol). Furthermore, it has been found that there is need for more attention to be geared on the momelotinib related peripheral neurotoxicity. A previous study has found that the incidence of peripheral neurotoxicity with momelotinib is between 10% and 40%.^20^ However, although the neurotoxicity of jaktinib is currently low, the overall incidence of all grades is less than 10%, but there is still need for longer follow‐up studies to verify its safety.

The current study had some limitations despite having reported a large data set on the efficacy and safety of jaktinib in JAK‐naïve patients with MF in China. There is need for further studies to verify the conclusion on the potential of jaktinib to improve anemia. This is because our study included few patients with severe anemia and blood transfusion dependence. Furthermore, the definition of transfusion‐ dependence is not fully accepted. It should be noted that the small population of patients with transfusion dependence is partly because of the difficulty of transfusion of red blood cells. In some areas, patients have the opportunity to receive transfusions of red blood cells only when their hemoglobin is below 55 g/L. This study included the results of karyotype, using DIPSS‐plus as a prognostic grouping tool, which may result in significantly fewer high‐risk patients in this study compared with using DIPSS or IPSS as prognostic grouping tool. Moreover, next generation sequencing studies were not conducted to identify high risk mutations and follow‐up data on bone marrow biopsies were not performed to investigate for the changes in fibrosis. The advantage of jaktinib with regard to long‐term survival could not be analyzed in the current study because of the short follow‐up duration. A phase III study on the comparison of jaktinib with hydroxyurea treatment in JAK‐naïve MF (NCT04617028) and phase II studies of jaktinib in patients with ruxolitinib intolerance (NCT04217993) as well as failure (NCT04851535) are currently underway. This is to improve the understanding on the role of jaktinib in patients with MF.

In conclusion, it was evident that jaktinib is safe and well‐tolerated in patients with MF in China. Furthermore, it was found that the 100 mg BID is significantly more effective in terms of splenic response as compared with that of the 200 mg QD. Therefore, the outstanding efficacy and mild side effects of jaktinib make it suitable for most patients with MF.

## AUTHOR CONTRIBUTIONS

Jie Jin and Liqing Wu conceived and designed the study, all authors were involved in data collection and assembly. Yi Zhang, Hu Zhou, Zhongxing Jiang, Dengshu Wu, and Liqing Wu were involved in data analysis and interpretation. Yi Zhang and Jie Jin contributed to manuscript writing, and all the authors read as well as approved the final version of this manuscript for publication. All the authors had access to all study data and agreed to be accountable for the accuracy and integrity of the data. The corresponding author had the final responsibility to submit the manuscript for publication.

## CONFLICT OF INTEREST

Liqing Wu is a staff employee of Suzhou Zelgen Biopharmaceuticals Co, Ltd, and reported the stock and other ownership of Suzhou Zelgen Biopharmaceuticals Co, Ltd. No other potential conflicts of interest were reported.

## Supporting information


**Appendix S1.** Supporting Information.

**Appendix S2.** Supporting Information.

## Data Availability

No data sharing plan was designed at the start of this study. Specific requests for non‐identifiable data for valid academic reasons as judged by the trial management group will be granted, with appropriate data sharing agreement, and should be addressed to the chief investigator (AKF).
