# Supplementary material for: Safety and efficacy of jaktinib in the treatment of Janus kinase inhibitor‐naïve patients with myelofibrosis: Results of a phase II trial
Source: Am J Hematol. 2022 Oct 4;97(12):1510–9. doi: 10.1002/ajh.26709 (PMC10092883; doi:10.1002/ajh.26709)
Supplement: Supplementary file 1 — Appendix S1. Supporting Information. [file AJH-97-1510-s002.docx]

|  | |
| --- | --- |
|  | |
| **Clinical Study Protocol** | |
|  |  |
| Name of Clinical Trial Protocol | A multi-center, phase II clinical trial evaluating the safety and efficacy of Jaktinib hydrochloride tablets in the treatment of patients with intermediate or high risk myelofibrosis |
| Clinical Trial Protocol Number | ZGJAK002 |
| Clinical Trial Phase | II |
| NMPA Approval Number | 2016L10574, 2016L10575 |
| Study Site and  Principal Investigator | Professor Jin Jie  The First Affiliated Hospital, Zhejiang University  No. 79, Qingchun Road, Hangzhou, Zhejiang 310058 |
| Sponsor | Suzhou Zelgen Biopharmaceuticals Co., Ltd.  No. 209, Chenfeng Road, KunShan, Suzhou, Jiangsu |
| Telephone/Fax | 0512-57309965/0512-57309965 |
| Clinical Trial Protocol Version | Mar. 22, 2020/Version 7. 0 |
| Alternative Version | Oct. 8, 2019/Version 6. 0 |
|  |  |
| The information contained herein is proprietary to Suzhou Zelgen Biopharmaceuticals Co., Ltd. This document is for restricted use. It must not be delivered, reproduced, published, or used (all or in part) without the explicit consent of Suzhou Zelgen Biopharmaceuticals Co., Ltd. | |

**Ethics Statement**

1. Documents

The design of this study protocol should follow the documents below:

1. Declaration of Helsinki
2. The Drug Administration Law of the People's Republic of China
3. Drug Registration Regulations
4. Good Clinical Practice
5. Guidelines for Laboratory Management of Biological Sample Analysis in Clinical Drug Trials (Trial Version)

2. Institutional Review Board and Ethics Committee

The investigator is required to submit the study protocol, blank CRF, informed consent form, regulatory approvals, certificate of analysis, and any subject recruitment advertisements to the institutional review board, ethics committee, or any organization with equivalent authority for approval. Written approval should be submitted by the investigator to Suzhou Zelgen Biopharmaceuticals Co., Ltd.

3. Informed Consent Form

The informed consent form should be submitted to the relevant ethics committee for approval. Each informed consent form must include relevant information specified in GCP.

The risks and benefits of the trial must be clearly explained to each potential subject before he/she participates in the trial. After explaining all the basic information related to the trial, and confirming that each potential subject has fully understood the purpose, risks and benefits of the trial, the investigator must require each participant to sign and date the informed consent form (in duplicate). Subjects must obtain one informed consent form.

**Investigator Signature Page - Lead Unit**

I have read this study protocol, confirmed that the protocol contains all the necessary contents about the implementation of the study, and fully understood the investigator's responsibilities. As the principal investigator, I will provide a copy of this protocol and other relevant information to all participating research personnel. I will discuss these information with all participating research personnel to ensure that they fully understand the investigational drug and how to conduct the trial. I agree to fulfill all my responsibilities in strict accordance with laws and regulations of China, Declaration of Helsinki, Good Clinical Practice, as well as the trial protocol.

Lead Unit: The First Affiliated Hospital, Zhejiang University

Principal Investigator Signature/Date

DD/MM/YYYY

**Investigator Signature Page - Participating Research Units**

I have read this study protocol, confirmed that the protocol contains all the necessary contents about the implementation of the study, and fully understood the investigator's responsibilities. As the principal investigator, I will provide a copy of this protocol and other relevant data to all participating research personnel. I will discuss these information with all participating research personnel to ensure that they fully understand the investigational drug and how to conduct the trial. I agree to fulfill all my responsibilities in strict accordance with laws and regulations of China, Declaration of Helsinki, Good Clinical Practice, as well as the trial protocol.

Hospital Name:

Principal Investigator Signature/Date

DD/MM/YYYY

**Sponsor Signature Page**

I have read this study protocol, confirmed that the protocol contains all the necessary contents about the implementation of the study, and fully understood the responsibilities related to the trial. I will provide qualified drugs and relevant information as required by the trial protocol. I agree to fulfill all my responsibilities in strict accordance with laws and regulations of China, Declaration of Helsinki, Good Clinical Practice, as well as the trial protocol.

Sponsor: Suzhou Zelgen Biopharmaceuticals Co., Ltd.

Sponsor Representative Signature/Date

DD/MM/YYYY

TABLE OF CONTENTS

[Ethics Statement 2](#_Toc90559144)

[Investigator Signature Page - Lead Unit 3](#_Toc90559145)

[Investigator Signature Page - Participating Research Units 4](#_Toc90559146)

[Sponsor Signature Page 5](#_Toc90559147)

[TABLE OF CONTENTS 6](#_Toc90559148)

[Abstract 10](#_Toc90559149)

[Schedule of Activities 18](#_Toc90559150)

[Flow Chart of the Study 25](#_Toc90559151)

[Abbreviations 26](#_Toc90559152)

[1. RESEARCH BACKGROUND AND RATIONALE 28](#_Toc90559153)

[1.1 Myelofibrosis and JAK Inhibitors 28](#_Toc90559154)

[1.2 Clinical Data on Momelotinib 30](#_Toc90559155)

[1.3 Clinical Data on Momelotinib B.I.D. 33](#_Toc90559156)

[1.4 Jaktinib Dose Escalation Study 35](#_Toc90559157)

[1.5 Introduction to the Investigational Drug 38](#_Toc90559158)

[1.5.1 Drug name and strength 38](#_Toc90559159)

[1.5.2 Type and mechanism of pharmacological action 39](#_Toc90559160)

[1.5.3 Theoretical basis of the study 39](#_Toc90559161)

[2. TRIAL OBJECTIVES 40](#_Toc90559162)

[3. TRIAL DESIGN 40](#_Toc90559163)

[3.1 Overall Design 40](#_Toc90559164)

[3.2 Dose Setting Basis 47](#_Toc90559165)

[3.3 Randomization and Blinding Method 48](#_Toc90559166)

[4. TRIAL POPULATION 49](#_Toc90559167)

[4.1 Inclusion Criteria 49](#_Toc90559168)

[4.2 Exclusion Criteria 49](#_Toc90559169)

[4.3 Treatment Discontinuation Criteria and Trial Termination Criteria 51](#_Toc90559170)

[4.3.1 Individual subjects discontinued 51](#_Toc90559171)

[4.3.2 Treatment discontinuationcriteria 51](#_Toc90559172)

[4.3.3 Trial termination 52](#_Toc90559173)

[5. INVESTIGATIONAL DRUG 53](#_Toc90559174)

[5.1 About the Investigational Drug 53](#_Toc90559175)

[5.2 Treatment Protocol 53](#_Toc90559176)

[5.3 Drug Packaging and Labeling 54](#_Toc90559177)

[5.4 Storage, Management, and Dispensing of Drugs 55](#_Toc90559178)

[5.5 Medication Record and Return and Treatment Compliance 55](#_Toc90559179)

[5.6 Responsibility on Drug Management 55](#_Toc90559180)

[5.7 Provisions of Concomitant Medications 55](#_Toc90559181)

[5.7.1 Restricted/permitted concomitant therapy 56](#_Toc90559182)

[5.7.2 Prohibited concomitant medications 56](#_Toc90559183)

[5.8 Compliance 57](#_Toc90559184)

[6. PROCEDURES 57](#_Toc90559185)

[6.1 Description 57](#_Toc90559186)

[7. TRIAL PROCEDURES 61](#_Toc90559187)

[7.1 Screening Period (D-28 to D-1) 61](#_Toc90559188)

[7.2 Baseline Period 62](#_Toc90559189)

[7.3 Treatment Period 63](#_Toc90559190)

[7.4 End-of-Treatment (EOT) Follow-Up 69](#_Toc90559191)

[7.5 Survival Follow-up 71](#_Toc90559192)

[8. ASSESSMENTS 71](#_Toc90559193)

[8.1 Safety Evaluations 71](#_Toc90559194)

[8.2 Efficacy Evaluation 71](#_Toc90559195)

[8.2.1 Primary efficacy endpoint 71](#_Toc90559196)

[8.2.2 Secondary Efficacy Endpoints 71](#_Toc90559197)

[8.2.3 Scanning and calculation of liver and spleen volumes 73](#_Toc90559198)

[8.3 Pharmacokinetics Study 73](#_Toc90559199)

[9. ADVERSE EVENTS AND SERIOUS ADVERSE EVENTS 74](#_Toc90559200)

[9.1 Adverse Events 74](#_Toc90559201)

[9.1.1 Definition of adverse event 74](#_Toc90559202)

[9.1.2 Collection and recording of adverse events 74](#_Toc90559203)

[9.1.3 Criteria for relationship between adverse events and the drug 75](#_Toc90559204)

[9.1.4 Severity grading scale for adverse events 76](#_Toc90559205)

[9.2 Serious Adverse Events 76](#_Toc90559206)

[9.2.1 Definition of serious adverse event 76](#_Toc90559207)

[9.2.2 Reporting of serious adverse events 77](#_Toc90559208)

[9.2.3 Responsibilities of various parties for serious adverse events 77](#_Toc90559209)

[9.2.4 Follow-up of serious adverse events 78](#_Toc90559210)

[9.3 Pregnancy 79](#_Toc90559211)

[10. DATA MANAGEMENT 79](#_Toc90559212)

[10.1 Electronic Case Report Form 79](#_Toc90559213)

[10.2 Establishment and Testing of Database, and Data Input and Modification 79](#_Toc90559214)

[10.3 Locking of Database 80](#_Toc90559215)

[11. STATISTICAL ANALYSIS 80](#_Toc90559216)

[11.1 Sample Size 80](#_Toc90559217)

[11.2 Study Population 81](#_Toc90559218)

[11.3 Baseline and Demographic Characteristics 82](#_Toc90559219)

[11.4 Efficacy Analysis 82](#_Toc90559220)

[11.4.1 Primary efficacy parameter 82](#_Toc90559221)

[11.4.2 Secondary efficacy parameters 82](#_Toc90559222)

[11.5 Safety Analysis 83](#_Toc90559223)

[11.6 PK Analysis 84](#_Toc90559224)

[11.7 Interim Analysis 84](#_Toc90559225)

[12. ETHICS 84](#_Toc90559226)

[12.1 Ethics Committee 84](#_Toc90559227)

[12.2 Informed Consent 85](#_Toc90559228)

[12.3 Subject Confidentiality 85](#_Toc90559229)

[13. TRIAL MANAGEMENT 85](#_Toc90559230)

[13.1 Independent Data Monitoring Committee 85](#_Toc90559231)

[13.2 Protocol Revision 86](#_Toc90559232)

[13.3 Training 86](#_Toc90559233)

[13.4 Standard Operation 86](#_Toc90559234)

[13.5 Monitoring 86](#_Toc90559235)

[13.6 Quality Control and Quality Assurance 87](#_Toc90559236)

[13.7 Audit and Inspection 88](#_Toc90559237)

[13.8 Trial Data Archiving 88](#_Toc90559238)

[14. EXPECTED PROGRESS AND COMPLETION DATE OF THE
CLINICAL TRIAL 88](#_Toc90559239)

[15. FOLLOW-UP AFTER THE COMPLETION OF THE TRIAL AND
MEDICAL MEASURES 88](#_Toc90559240)

[16. RESPONSIBILITIES OF EACH PARTY 89](#_Toc90559241)

[16.1 Responsibilities of the Investigator 89](#_Toc90559242)

[16.2 Responsibilities of the Sponsor 90](#_Toc90559243)

[17. CONFIDENTIALITY AND PUBLICATION OF TRIAL RESULTS 91](#_Toc90559244)

[18. REGULATORY AGENCY 91](#_Toc90559245)

[18.1 Sponsor 91](#_Toc90559246)

[18.2 Trial Site 91](#_Toc90559247)

[18.2.1 Lead unit 91](#_Toc90559248)

[18.2.2 Participating sites 91](#_Toc90559249)

[19. REFERENCES 92](#_Toc90559250)

[Appendix I. European Consensus on Grading of
Myelofibrosis (MF) 93](#_Toc90559251)

[Appendix II. Diagnostic Criteria for PMF (WHO 2016) 94](#_Toc90559252)

[Appendix III. Diagnostic Criteria for Post-PV-MR and
Post-ET-MF (IWG-MRT) 95](#_Toc90559253)

[Appendix IV. Prognostic Scoring Systems for MF 96](#_Toc90559254)

[Appendix V. MPN-SAF TSS Assessment 97](#_Toc90559255)

[Appendix VI. Response Criteria for Myelofibrosis (IWG-MRT) 98](#_Toc90559256)

[Appendix VII. Strong CYP3A4 Inducers/Inhibitors/CYP 2B6
Substrates 99](#_Toc90559257)

[Appendix VIII. QTcB Formula 99](#_Toc90559258)

[Appendix IX. Glucocorticoids Dose Equivalence Conversion 99](#_Toc90559259)

Abstract

| Sponsor | Suzhou Zelgen Biopharmaceuticals Co., Ltd. |
| --- | --- |
| Study Title | A multi-center, phase II clinical trial evaluating the safety and efficacy of Jaktinib hydrochloride tablets in the treatment of patients with intermediate or high risk myelofibrosis |
| Version/Date | Version 7.0/Mar. 22, 2020 |
| Trial Phase | Phase II, registered clinical trial |
| Investigational Drug Name | Jaktinib hydrochloride tablets |
| Indication | For the treatment of intermediate or high risk myelofibrosis, including primary myelofibrosis (PMF), post polycythemia vera myelofibrosis (post-PV MF), or post essential thrombocythemia myelofibrosis (post-ET MF). |
| Study Design | The objective of this trial is to evaluate the safety and efficacy of Jaktinib hydrochloride tablets in the treatment of patients with intermediate or high risk myelofibrosis, and to analyze the pharmacokinetic (PK) and pharmacodynamic properties in the study population.  This is a single-arm, open-label, multi-center, randomized, two-stage study. Around 100 subjects are expected to be enrolled at about 25 study sites during Stage 1 and an additional 36 subjects are expected to be enrolled into the expansion cohort during Stage 2, i.e. approximately 136 subjects in total. |
|  | Two dose groups are set up for Stage 1, i.e., 100 mg b.i.d. and 200 mg q.d. The subjects are randomized in a 1:1 ratio, with 50 subjects per group for a total of 100 subjects. In addition to the 100 subjects already randomized, an additional 36 subjects will be enrolled into the expansion cohort during Stage 2.  Based on the interim analysis results of the Jaktinib ZGJAK002 trial, the risks and benefits of 100 mg b.i.d. and 200 mg q.d. groups are evaluated. The principal investigator and the sponsor have decided that all of the 36 subjects in the expansion group will be given 100 mg b.i.d.  All enrolled subjects will be treated for at least 4 cycles, with 6 weeks per cycle or 24 weeks in total. Subjects who do not meet the treatment discontinuation criteria after 24 weeks of treatment may continue using the investigational drug until any of the drug discontinuation criteria specified in the protocol is met. Safety evaluation will be carried out every 1–2 weeks in the first treatment cycle, every 3 weeks in Cycles 2–4, and every 6 weeks after Week 24. Evaluation will be carried out once every 12 weeks after 48 weeks of treatment.  In this study, 12 subjects/group (12 pairs, 24 subjects in total) will be selected for PK analysis. Subjects participating in the PK analysis are required to be hospitalized during the PK blood sampling period, from 1 day before dose administration (D-1) to D7 of dose administration. Subjects will be discharged on D7.  In this study, around 7 subjects/group (7 pairs, 14 subjects in total) will be selected for phosphorylated STAT3 (pSTAT3) analysis as a preliminary pharmacodynamics study. Subjects participating in the pSTAT3 analysis are required to be hospitalized during the blood sampling period, from 1 day before Week 24 or 30 (D168 or D210 after randomization and enrollment) of treatment to D169 or D211 after randomization and enrollment. |
|  | A steering committee may be appointed if necessary, which consists of at least the PI, physicians and statisticians with relevant clinical expertise. The steering committee will evaluate and provide the following recommendations: Modify the dose and dosing frequency based on safety considerations, or terminate the phase II trial. |
| Dosing regimen | - The medication schemes of each dose group are shown as follows:  \| Dose group \| Dosing regimen \| Medication duration \| \| --- \| --- \| --- \| \| Jaktinib hydrochloride tablets, 100 mg b.i.d. \| Orally twice per day, 100 mg per dose, once every 12 hours, on an empty stomach \| Treatment will continue until any of the discontinuation criteria specified by the protocol is met. \| \| Jaktinib hydrochloride tablets, 200 mg q.d. \| Orally once per day, 200 mg per dose, once daily, on an empty stomach \| \| * Fasting conditions: 0.5-1 hour before the meal or 2 hours after the meal. **Do not take the drug after a meal**. \| \| \|  - Dose modification plan: The dose of Jaktinib hydrochloride tablets may be adjusted during treatment according to platelet count and neutrophil count (CTCAE 4.03) (including dose reduction or interruption due to adverse reactions; as well as dose resumption or dose increase upon resolution of the adverse reaction). The specific protocol is shown as follows:  \| Platelet count \| ▼Jaktinib hydrochloride tablets groups▼ \| \| \| --- \| --- \| --- \| \| 100 mg (2 tablets) b.i.d. \| 200 mg (4 tablets) q.d. \| \| ▼Dose adjustments for Jaktinib hydrochloride tablets▼ \| \| \| ≥ 100 × 10^9^/L \| Original dose or increased dose \| Original dose or increased dose \| \| 75 - <100×10^9^/L \| 150 mg (3 tablets) q.d. \| 150 mg (3 tablets) q.d. \| \| 50 - < 75 × 10^9^/L \| 100 mg (2 tablets) q.d. \| 100 mg (2 tablets) q.d. \| \| < 50 × 10^9^/L \| Temporary discontinuation \| Temporary discontinuation \| \| Absolute neutrophil count \| ▼Jaktinib hydrochloride tablets groups▼ \| \| \| 100 mg (2 tablets) b.i.d. \| 200 mg (4 tablets) q.d. \| \| ▼Dose adjustments for Jaktinib hydrochloride tablets▼ \| \| \| ≥ 1.5 × 10^9^/L \| Original dose \| Original dose \| \| 1.0- <1.5×10^9^/L \| 150 mg (3 tablets) q.d. \| 150 mg (3 tablets) q.d. \| \| 0.5- <1.0×10^9^/L \| 100 mg (2 tablets) q.d. \| 100 mg (2 tablets) q.d. \| \| <0.5×10^9^/L \| Temporary discontinuation \| Temporary discontinuation \| \| - The investigator may increase the dose whenever clinically indicated if PLT is persistently ≥ 100 × 10^9^/L and the drug is well-tolerated by the subject. Each dose increase should generally not exceed 50 mg/d and the maximum dose should not exceed 250 mg/d. Routine blood tests should be performed during unscheduled visits 2 and 4 weeks after the dose increase. Unscheduled visits may be skipped if they fall within the time window of other study visits. - During the study, treatment may be interrupted up to 3 weeks per cycle (6 weeks) due to hematological toxicities. A subject should withdraw from the study if treatment is interrupted for > 3 weeks. \| \| \| |
| Total Number of Subjects | Approximately 100 subjects will be enrolled during Stage 1, with 50 subjects per group. In accordance with the results of clinical trials published by Momelotinib and the results of the COMFORT-I trial studying Ruxolitinib in the treatment of MF, assuming Jaktinib has a 48% response rate (efficacy indicator: reduction in spleen volume at Week 24 ≥ 35%), and the dropout rate is no more than 10%, then 50 subjects can ensure that the width of 95% confidence interval of the response rate does not exceed ± 15%.  The 100 mg b.i.d. group will be expanded during Stage 2. Assuming the efficacy of Jaktinib for the treatment of MF (efficacy endpoint: ≥ 35% reduction in spleen volume at 24 weeks) is 48% (no less than 23%), with 80% power, 95% confidence interval (two-sided), and a drop-out rate no more than 10%, then the sample size for Stage 2 is 36 subjects.  A total of 136 subjects will be enrolled for this study. |
| Number of Study Sites | Around 25 study sites across the country |
| Study Period | Approximately 36 months |
| Selection Criteria | Inclusion Criteria |
|  | 1. Age ≥ 18 years, either male or female; 2. Patients diagnosed with PMF according to WHO criteria (2016 Edition), or patients diagnosed with Post-PV-MF or Post-ET-MF according to IWG-MRT diagnostic criteria, with or without JAK2 mutations; 3. Patients with intermediate-2 or high risk myelofibrosis according to the DIPSS-plus scoring system; (Note: Patients with intermediate-1, intermediate-2 or high risk myelofibrosis are included in Study Protocol V1.0-6.0; Patients with intermediate-2/high risk myelofibrosis are included in Study Protocol V7.0.) 4. Subjects with no recent plans for stem cell transplantation; 5. Expected survival period is greater than 24 weeks; 6. ECOG PS 0-2; 7. Splenomegaly: greater than or equal to at least 5 cm below left costal margin by palpation; 8. Peripheral blood blasts ≤ 10%; 9. Patients who have not received prior treatment with JAK inhibitors; (Note: Patient who received treatment with JAK inhibitors for no more than 10 days may be enrolled) 10. Platelet count ≥ 75 × 10^9^/L and ANC ≥ 1000/μL without the aid of colony-stimulating factors, growth factors, thrombopoietin, or platelet transfusion. Subjects who have not received growth factors, colony-stimulating factors, thrombopoietin, or platelet infusion within 2 weeks prior to screening examinations; 11. Normal organ function within 7 days prior to randomization and enrollment: ALT and AST ≤ 2.5×ULN; DBIL and TBIL ≤ 2.0×ULN; Serum creatinine ≤ 1.5×ULN;  CrCI ≥ 50 mL/min; 12. Subjects who meet the requirements of ethics committee, and voluntarily sign the informed consent form; 13. Ability to comply with the trial and follow-up procedures. |
|  | Exclusion Criteria   1. Any significant clinical and laboratory abnormality which, in the opinion of the investigator, may affect the safety evaluation:   a. Uncontrolled diabetes (> 250 mg/dL or > 13.9 mmol/L), b. Hypertension that cannot return to the following range (systolic blood pressure < 160 mmHg, diastolic blood pressure < 100 mmHg) despite treatment with one or two antihypertensive drugs, c. Peripheral neuropathy (NCI-CTC AE v4.03 Grade ≥ 2);   1. Patients with a history of congestive heart failure, uncontrolled or unstable angina or myocardial infarction, cerebrovascular accident, or pulmonary embolism within 6 months prior to screening; 2. Patients who have not fully recovered from surgical operation within 4 weeks prior to screening; 3. Patients with arrhythmia requiring treatment, or QTcB > 480 ms during screening; 4. Subjects with clinical symptoms of bacterial, viral, parasitic or fungal infections requiring treatment during screening; 5. Patients with a history of congenital or acquired bleeding disorders; 6. Patients who had undergone splenectomy, or received radiotherapy to the spleen within 12 months before screening; 7. Patients with HIV positive, HBV positive (HBsAG positive, HBV-DNA positive or ≥ 1000 copies/mL), anti-HCV positive or HCV-RNA positive results during screening; 8. Patients with epilepsy or treated with psychotropic drugs or sedatives (Note: except for estazolam tablets) at the time of screening; 9. Female patients who are planning pregnancy, already pregnant or breastfeeding, as well as patients who are unable to adopt effective contraceptive measures during the entire course of the trial; Male patients who do not use condoms during treatment and for 2 days (around 5 half-lives) after the last dose; 10. Patients who had malignant tumors within the past 5 years (except for basal cell carcinomas and cervical carcinoma in situ that have been cured); 11. Subjects with other serious concurrent diseases which, in the opinion of the investigator, may affect patient safety or compliance; 12. Subjects with suspected allergies to Jaktinib or other similar drugs; 13. Patients who have participated in another clinical trial involving a new drug or medical device and used the investigator drug or medical device within 3 months prior screening; 14. Patients who have received any medical treatment for MF (such as hydroxyurea), any immunomodulator (such as thalidoamide), any immunosuppressants, prednisone ≥ 10 mg/day or equivalent glucocorticoids, or growth factors (such as EPO) within 2 weeks prior to the randomization and enrollment, or within 6 half-lives of the drug; |
| Pharmacokinetics  Assessment | All subjects participating in the PK study must undergo blood sample collection according to trial requirements.  PK sampling points are as follows:   \| Study time (day) \| PK blood sampling points \| \| \| \| \| \| \| \| \| --- \| --- \| --- \| --- \| --- \| --- \| --- \| --- \| --- \| \| Before administration \| After administration \| \| \| \| \| \| \| \| 1.0h \| 2.0h \| 3.0h \| 4.0h \| 6.0h \| 8.0h \| 12h \| \| D1 \| X \| X \| X \| X \| X \| X \| X \| X \| \| D2 \| X \|  \|  \| X \|  \|  \|  \|  \| \| D3 \| X \|  \|  \| X \|  \|  \|  \|  \| \| D4 \| X \|  \|  \| X \|  \|  \|  \|  \| \| D5 \| X \|  \|  \| X \|  \|  \|  \|  \| \| D6 \| X \|  \|  \| X \|  \|  \|  \|  \| \| D7 \| X \|  \|  \|  \|  \|  \|  \|  \| \| Blood sampling at the median cubital vein, 4 mL each time;  Before administration: within 10 min before administration;  1-4 hours after administration: within ± 5 minutes;  6-12 hours after administration: within ± 10 minutes;  12 hours after administration: Must be collected prior to the evening dose.  Subjects participating in the PK analysis are required to be hospitalized during the PK blood sampling period, from 1 day before dose administration (D-1) to D7 of dose administration. Subjects will be discharged on D7. \| \| \| \| \| \| \| \| \|   During the trial, blood samples for pharmacokinetic analysis are collected at scheduled time points according to the protocol. All samples should be pre-frozen within 50 minutes from the beginning of collection.  The plasma concentration is determined using HPLC-MS/MS. Main pharmacokinetic parameters include: AUC_last,_ AUC_inf_, C_max_, T_max_, CL/F, V_d_/F, and t_1/2_.  Subjects participating in the pSTAT3 analysis during Week 24 or 30 of the treatment period will be required to undergo blood sampling according to the following schedule:   \| Study time (day) \| Blood sampling time points \| \| \| \| \| \| \| \| --- \| --- \| --- \| --- \| --- \| --- \| --- \| --- \| \| Before administration \| After administration \| \| \| \| \| \| \| 1.0h \| 2.0h \| 4.0h \| 6.0h \| 8.0h \| 12h \| \| D168 or D210 \| X \| X \| X \| X \| X \| X \| X \| \| D169 or D211 \| X \|  \|  \|  \|  \|  \|  \| \| Blood sampling at the median cubital vein, 4 mL each time;  Before administration: within 10 min before administration;  1-4 hours after administration: within ± 5 minutes;  6-12 hours after administration: within ± 10 minutes;  12 hours after administration: Must be collected prior to the evening dose.  Subjects participating in the pSTAT3 analysis are required to be hospitalized during the blood sampling period, which is  from 1 day before Week 24 or 30 (D167 or D209 after randomization) of treatment to D169 or D211 after randomization. \| \| \| \| \| \| \| \| |
| Efficacy Assessment | Primary efficacy endpoint   - Response rate: the proportion of subjects with spleen volume reduction ≥ 35% at Week 24;   Secondary efficacy endpoints   - Objective response rate (CR + PR): IWG-MRT response criteria - Spleen Response: - Best response rate: the proportion of subjects with at least one spleen volume reduction ≥ 35% against the baseline; - Time to response: the time from the date of randomization and enrollment to the first date of ≥ 35% reduction in spleen volume from baseline; - DoMSR: the time between the first occurrence of spleen volume reduction of ≥ 35% from baseline to an increase in spleen volume so that the reduction is less than 35% from baseline; - Anemia Response: - Proportion of transfusion-dependent patients at baseline turned into transfusion-independent patients (transfusion-independent patients: no transfusion for at least 12 consecutive weeks and HGB ≥ 85 g/L) - Proportion of HGB elevation ≥ 20 g/L in transfusion-independent patients  (HGB ≤ 100 g/L) at baseline; - Reduced RBC-transfusion-dependence: 50% decrease in the number of RBC transfusions.   Patients who are RBC-transfusion-dependent at baseline are defined as patients who received ≥ 2U of RBC transfusion within 30 days prior to treatment with the investigational drug   - MF-related symptoms: proportion of patients with MPN-SAF TSS decreasing by ≥ 50%   The decrease in MPN-SAF TSS from baseline   - PFS: The time from the date of randomization and enrollment to the date of any of the following events: 1) ≥ 25% increase in spleen volume from nadir (including baseline);  2) death from any cause. - LFS: The time from the date of randomization and enrollment to the date of any of the following events: 1) first bone marrow blasts ≥ 20%; 2) first peripheral blood blasts  ≥ 20% and absolute blast count ≥ 1 × 10^9^/L lasting for at least 2 weeks; 3) death from any cause. - OS: The time from the date of randomization and enrollment to the date of death from any cause; |
| Safety Evaluations | Safety endpoint: Severity and incidence of adverse events and adverse reactions that occur from randomization and enrollment to 28 days after treatment discontinuation, including overall safety assessments and laboratory tests.  Safety Assessment Contents:   - Overall safety assessments: vital signs, physical examination, and ECOG PS score; - Laboratory safety tests: include routine blood, routine urinalysis, blood biochemistry, liver and kidney function tests, and coagulation test for each safety follow-up; - 12-Lead ECG, color Doppler electrocardiography (performed if 12-Lead ECG shows abnormal results with clinical significance); - Severity and incidence of adverse events and adverse reactions. - Incidence of thrombotic events. - Arterial thrombosis: 1) coronary atherosclerosis heart disease; 2) cerebral artery thrombosis; 3) peripheral arterial occlusive disease: such as mesenteric arterial thrombosis and arterial thrombosis of extremities. - Venous thrombosis: 1) thrombophlebitis; 2) deep vein thrombosis; 3) pulmonary embolism. - Microcirculatory thrombosis: 1) thrombotic thrombocytopenic purpura; 2) hemolytic uremic syndrome; 3) thrombosis in extracorporeal circulation; 4) other conditions: such as purpura fulminans and disseminated intravascular coagulation |
| Statistical Analysis | **Statistical Analysis Sets**  The analysis population of this trial include Full Analysis Set (FAS), Per Protocol Set (PPS), Safety Set (SS), and PK set.  **Full Analysis Set (FAS):**  FAS is used for dropout analysis, equilibrium analysis of basic parameters, and primary efficacy endpoint analysis.  According to the Intention-to-Treat (ITT) principle, the FAS includes all randomized and enrolled subjects which have received at least 1 dose of Jaktinib per protocol. Subjects with the any of the following will be excluded from FAS: 1) missing baseline radiographic assessment; or 2) baseline radiographic assessment not reviewed by IRC. For cases with no PFS and LFS data obtained, the PFS or LFS shall be considered censored, and the censoring time shall be the latest time of confirmed non-PD. In the efficacy analysis, subjects who do not receive the allocated study treatment will be still summarized by the allocated treatment group.  **Per Protocol Set (PPS):**  The PPS is a subset of the FAS, which includes all randomized and enrolled subjects who have received at least 12 weeks of Jaktinib treatment and completed at least 1 efficacy assessment per protocol. Subjects who haven't completed 12 weeks of treatment but have clear medical evidence and disease progression after starting treatment are also included in the PPS. For cases with no PFS and LFS data obtained, the PFS or LFS shall be considered censored, and the censoring time shall be the latest time of confirmed non-PD.  Efficacy is analyzed in the FAS and PPS, with PPS as the primary analysis results. Detailed methods of missing data processing and sensitivity analysis are described in the Statistical Analysis Plan (SAP).  **Safety Set (SS):**  All subjects who signed the informed consent form, and received at least one dose of Jaktinib. In the safety analysis, subjects who do not receive the allocated study treatment will be summarized by the actual treatment. All safety analysis are performed in the SS.  **PK Analysis Set (PKAS):**  PKAS includes all subjects who received at least one dose of Jaktinib, and had at least one drug concentration sample, collected according to the scheduled PK time points, with no major protocol violations that may significantly affect the PK assessment. The PKAS is used for PK analysis.  Before locking the database, the major protocol violation shall be defined and various sets for analysis shall be finalized by the principal investigator, statisticians and the sponsor together. |
|  | **Statistical Analysis**  SAS 9.4 software is used for the statistical analysis of the efficacy data. The data sets are determined based on the intention-to-treat (ITT) principle. Two-sided tests were performed for all statistical analyses. |
|  | **Safety Analysis:** The number and percentage of adverse reactions in each treatment group will be described. |
|  | **Compliance analysis**: The scheduled and actual administered dose in each treatment group will be described using mean, standard deviation, median, maximum, and minimum. |
|  | **Efficacy Analysis:** |
|  | Primary efficacy measures: Response rates of Jaktinib at various doses and their 95% confidence intervals. |
|  | Secondary efficacy measures: The progression rate and mortality rate are estimated by the Kaplan-Meier estimator, and the median PFS, LFS, OS and 95% confidence intervals are calculated. The HR of each covariate and the corresponding 95% confidence interval will be estimated using Cox regression. Covariates in COX regression model include treatment group (different Jaktinib groups), cause (primary vs. secondary), DIPSS-plus categorization (intermediate vs. high risk), age (18 to 60 years vs. over 60 years), and JAK2 V617F (***mut*** vs. ***wt***). A *p*-value of less than 0.05 is considered statistically significant. |
|  | **Pharmacokinetic Analysis:**  Appropriate descriptive statistics are listed to summarize the plasma concentrations of Jaktinib hydrochloride and its metabolites in each dose group. If necessary, the plasma concentration of Jaktinib hydrochloride and its metabolites may be used in pharmacokinetics or combined pharmacokinetics/pharmacodynamics analysis to further explore Jaktinib hydrochloride’s pharmacokinetics and the relationships between exposure and safety/efficacy. Based on the analysis, a separate analysis plan shall be prepared and the results will be separately reported (not provided in the clinical study report).  **Interim Analysis:**  An interim analysis is planned for this study to evaluate the safety, efficacy data, and pharmacokinetic properties. The same statistical methods will be used for the interim analysis and the final analysis.  Refer to the Statistical Analysis Plan for details. |

**Schedule of Activities**

| **Item** | **Screening period^1^** | **Baseline^1^** | **Treatment period** | | | | | | | | | | | | **End-of- treatment^2^** | **Safety follow-up^2^** | **Survival follow-up** | |
| --- | --- | --- | --- | --- | --- | --- | --- | --- | --- | --- | --- | --- | --- | --- | --- | --- | --- | --- |
| **Visit** | **SV** | **V0** | **V1** | **V2** | **V3** | **V4** | **V5** | **V6** | **V7** | **V8** | **V9** | **V10** | **V11, V12, V13...** | | **EOT** | **Within 1 month after EOT** | **Within 6 months**  **after EOT** | **Within 6 months**  **Over 6 months after EOT** |
| **Time from the  baseline (V0)** | **D-28 to D-1** | **d1** | **1w** | **2W** | **4W** | **6W** | **9W** | **12W** | **15W** | **18W** | **21W** | **24W** | **W24-48: every 6 weeks** | **From W48 onwards: every 12 weeks** | **Last dose** | **D28 after the last dose** | **Every 3 months** | **Every 6 months** |
| **Time window** |  |  | **±1d** | **±1d** | **±1d** | **±3d** | **±3d** | **±3d** | **±3d** | **±3d** | **±3d** | **±3d** | **±3d** | **±3d** | **+7d** | **±7d** | **±7d** | **±7d** |
| **Signing of Informed Consent Form**^3^ | × |  |  |  |  |  |  |  |  |  |  |  |  |  |  |  |  |  |
| **Inclusion/Exclusion Criteria** | × | × |  |  |  |  |  |  |  |  |  |  |  |  |  |  |  |  |
| **Baseline Demographics/Previous Medication/Medical History** | × |  |  |  |  |  |  |  |  |  |  |  |  |  |  |  |  |  |
| **Randomization** |  | × |  |  |  |  |  |  |  |  |  |  |  |  |  |  |  |  |
| **Safety Evaluation** |  |  |  |  |  |  |  |  |  |  |  |  |  |  |  |  |  |  |
| Physical Examination | × | × | × | × | × | × | × | × | × | × | × | × | × | × | × | × |  |  |
| Vital Signs | × | × | × | × | × | × | × | × | × | × | × | × | × | × | × | × |  |  |
| ECOG Score | × | ×^15^ |  |  |  | × |  | × |  | × |  | × | × | × | × | × |  |  |
| Routine Blood Test^4^ | × | ×^15^ | × | × | × | × | × | × | × | × | × | × | × | × | × | × |  |  |
| Routine Urinalysis^4^ | × | ×^15^ |  |  |  | × |  | × |  | × |  | × | × | × | × | × |  |  |
| Blood Biochemistry (Including Liver and Kidney Function Tests)^4^ | × | ×^15^ |  |  |  | × |  | × |  | × |  | × | × | × | × | × |  |  |
| Coagulation Test^4^ | × | ×^15^ |  |  |  | × |  | × |  | × |  | × | × | × | × | × |  |  |
| Serum EPO | × |  |  |  |  | × |  | × |  | × |  | × | × | × | × |  |  |  |
| pSTAT3^16^ |  |  |  |  |  |  |  |  |  |  |  | ×^16^ | ×^16^ |  |  |  |  |  |
| Blood Pregnancy Test^5^ | × | ×^15^ |  |  |  |  |  |  |  |  |  |  |  |  |  | × |  |  |
| Infection Test^6^ | × |  |  |  |  |  |  |  |  |  |  |  |  |  |  |  |  |  |
| Peripheral Blood Smear^7^ | × |  |  |  |  | × |  | × |  | × |  | × | × | × | × |  |  |  |
| Bone Marrow Smear | × |  |  |  |  | ×^13^ |  | ×^13^ |  | ×^13^ |  | ×^13^ | ×^13^ | ×^13^ | ×^13^ |  |  |  |
| Mutation Test^8^ | × |  |  |  |  |  |  |  |  |  |  |  |  |  | ×^14^ |  |  |  |
| Bone Marrow Aspiration and Biopsy^9^ | × |  |  |  |  | ×^13^ |  | ×^13^ |  | ×^13^ |  | ×^13^ | ×^13^ | ×^13^ | ×^13^ |  |  |  |
| Electrocardiography | × | ×^15^ |  |  |  | × |  | × |  | × |  | × | × | × | × | × |  |  |
| Color Doppler Echocardiography^10^ | × |  |  |  |  | ×^10^ |  | ×^10^ |  | ×^10^ |  | ×^10^ | ×^10^ | ×^10^ | ×^10^ | × |  |  |
| **Efficacy Assessment** |  |  |  |  |  |  |  |  |  |  |  |  |  |  |  |  |  |  |
| MRI/CT (Spleen Response Assessment)^11^ | × |  |  |  |  |  |  | × |  |  |  | × | × (every 12W) | × | × |  |  |  |
| MF Efficacy Assessment**^17^** |  |  |  |  |  |  |  | × |  |  |  | × | × (every 12W) | × | × |  |  |  |
| Anemia Response Assessment | × |  |  |  |  | × |  | × |  | × |  | × | × | × | × |  |  |  |
| MPN-SAF TSS Assessment | × |  |  |  |  | × |  | × |  | × |  | × | × | × | × |  |  |  |
| **PK Sampling^12^** |  | ×**^12^** | ×**^12^** |  |  |  |  |  |  |  |  |  |  |  |  |  |  |  |
| **Drug Dispensing/Retrieval** |  | × | × | × | × | × | × | × | × | × | × | × | × | × | × |  |  |  |
| **Subject Diary Card Dispensing/Retrieval** |  | × | × | × | × | × | × | × | × | × | × | × | × | × | × |  |  |  |
| **Concomitant Medication/Therapy** | × | × | × | × | × | × | × | × | × | × | × | × | × | × | × | × | × | × |
| **Adverse Event Record** | × | × | × | × | × | × | × | × | × | × | × | × | × | × | × | × |  |  |
| **Survival Status Record** |  |  |  |  |  |  |  |  |  |  |  |  |  |  |  | | × | × |

1. Results of radiographic assessments, bone marrow aspiration and biopsy, bone marrow smear, and mutation test obtained within 4 weeks prior to randomization and enrollment may be used as screening results with the investigator’s approval, under which circumstances reexaminations are not required during screening. Radiographic assessments must be confirmed by the IRC.
2. All subjects (including those who withdraw from the study early) must complete all examinations required for the EOT visit upon treatment discontinuation and start of new anti-MF treatment.
3. A signed informed consent form is required during the screening.
4. The content for various investigations includes:

- For routine blood test: Red blood cell (RBC), hemoglobin (HGB), platelet (PLT) and white blood cell (WBC) and their percentages, differential counts and percentages of WBC (basophil BASO#, eosinophil EO#, lymphocyte LYMPH#, monocyte MONO#, and neutrophil NEUT#);
- For routine urinalysis: pH, protein (U-PRO), glucose (U-GLU), white blood cell (U-WBC), urinary red blood cell (U-RBC), etc.;
- Blood biochemistry (including liver and kidney function): ALT, AST, ALP, γ-GT, TBIL, DBIL, total protein, albumin, LDH, UREA/BUN, sCr, electrolytes (K+, Na^+^, Cl^-^, Ca^2+^, Mg^2+^, and P), and glucose;
- For coagulation test: PT, TT, APTT, and INR.

1. For women of child-bearing age only. During the trial, pregnancy test should be performed for any suspected pregnancy or patients with probability of pregnancy judged by the investigator.
2. Serologic tests: Hepatitis b (Hep B panel + HBV-DNA) and hepatitis c (HCV antibodies + HCV-RNA) tests, syphilis antibodies, and HIV antibodies.
3. Peripheral blood smear: including cell morphology, counts and percentages.
4. Mutation test: Including JAK2 V617F (quantitative and qualitative), CALR (qualitative), and MPL W515L/K (qualitative), and BCR-ABL genetic tests. Genetic testing is carried out by the central laboratory.
5. Bone marrow aspiration and biopsy: cytological analysis, reticular fiber (silver) staining, chromosome karyotyping (peripheral blood specimens may be used in case of ‘dry tap’; may be tested at the central laboratory if unavailable at the study site), and pathological biopsy.
6. Performed when 12-lead ECG shows a clinically significant abnormality. Only document left ventricular ejection fraction.
7. Screening MRI/CT results must be reviewed by the IRC, and spleen volume must be measurable for the subject to be enrolled. Assess once every 12 weeks thereafter. Perform a tumor assessment once every 12 weeks during follow-up for subjects who discontinue the study treatment for reasons other than PD, until PD, withdrawal of consent, or start of new anti-MF treatment. During the trial, the investigator may perform unplanned MRI/CT test if a subject is suspected of PD. (Refer to the SOP of the IRC for details)
8. For subjects in PK subset only. PK sampling points are as follows:

| Study time (day) | PK blood sampling points | | | | | | | |
| --- | --- | --- | --- | --- | --- | --- | --- | --- |
|  | Before administration | After administration | | | | | | |
|  |  | 1.0h | 2.0h | 3.0h | 4.0h | 6.0h | 8.0h | 12h |
| D1 | X | X | X | X | X | X | X | X |
| D2 | X |  |  | X |  |  |  |  |
| D3 | X |  |  | X |  |  |  |  |
| D4 | X |  |  | X |  |  |  |  |
| D5 | X |  |  | X |  |  |  |  |
| D6 | X |  |  | X |  |  |  |  |
| D7 | X |  |  |  |  |  |  |  |
| Blood sampling at the median cubital vein, 4 mL each time;  Before administration: within 10 min before administration;  1-4 hours after administration: within ± 5 minutes;  6-12 hours after administration: within ± 10 minutes;  12 hours after administration: Must be collected prior to the evening dose.  Subjects participating in the PK analysis are required to be hospitalized during the PK blood sampling period, from 1 day before dose administration (D-1) to D7 of dose administration. Subjects will be discharged on D7. | | | | | | | | |

1. Perform a bone marrow smear only if the peripheral blood blasts are ≥ 20%; perform bone marrow biopsy only if considered necessary by the investigator. Perform a peripheral blood smear or bone marrow smear (only if the peripheral blood blasts are ≥ 20%) once every 12 weeks during follow-up for subjects who discontinue the study treatment for reasons other than PD, until PD, withdrawal of consent, or start of new anti-MF treatment. The investigator may perform unscheduled peripheral blood smear or bone marrow smear (only if the peripheral blood blasts are ≥ 20%) at any time during the study if a subject is suspected of progression.
2. Perform a JAK2 V617F *mut* test (quantitative and qualitative) only in subjects who test positive for JAK2 V617F *mut* at baseline (qualitative).
3. Tests performed within 7 days prior to randomization and enrollment do not need to be repeated before the first dose.
4. Subjects participating in the pSTAT3 analysis during Week 24 or 30 of the treatment period will be required to undergo blood sampling according to the following schedule (pSTAT3 is tested at the central laboratory):

| Study time (day) | Blood sampling time points | | | | | | |
| --- | --- | --- | --- | --- | --- | --- | --- |
|  | Before administration | After administration | | | | | |
|  |  | 1.0h | 2.0h | 4.0h | 6.0h | 8.0h | 12h |
| D168 or D210 | X | X | X | X | X | X | X |
| D169 or D211 | X |  |  |  |  |  |  |
| Blood sampling at the median cubital vein, 4 mL each time;  Before administration: within 10 min before administration;  1-4 hours after administration: within ± 5 minutes;  6-12 hours after administration: within ± 10 minutes;  12 hours after administration: Must be collected prior to the evening dose.  Subjects participating in the pSTAT3 analysis are required to be hospitalized during the blood sampling period, which is  from 1 day before Week 24 or 30 (D167 or D209 after randomization) of treatment to D169 or D211 after randomization. | | | | | | | |

1. Radiographic (MRI/CT) confirmation is required if spleen response is used to assess treatment efficacy. The investigator may perform unscheduled MRI/CT.

Flow Chart of the Study

**200mg qd, 24w**

**(N=50)**

**100mg bid, 24w**

**(N=50)**

**R**

R: Randomization E: Evaluation w: weeks

a: Stage 1: Approximately 100 subjects will be randomized to the two dose groups in a 1:1 ratio

b: Stage 2: An additional 36 subjects added to the 100 mg b.i.d. group

**Subjects may continue treatment with the investigational drug beyond 24 weeks until any of the discontinuation criteria specified by the protocol is met.**

- **PMF/Post-PV-MF /Post-ET-MF**
- **Intermediate-2/High Risk**
- **ECOG ≤ 2**
- **Spleen at least 5 cm below left costal margin by palpation**
- **Peripheral blood blasts ≤ 0.1**
- **JAK inhibitor-naive**

**E**

Abbreviations

| **Abbreviation** | **English Full Name** |
| --- | --- |
| allo-HSCT | Allogeneic-hematopoietic stem cell transplantation |
| ANC | Absolute neutrophil count |
| AL | Acute leukemia |
| AUC | Area under the curve |
| a GVHD | Acute graft versus host disease |
| BAT | Best available therapy |
| c GVHD | Chronic graft versus host disease |
| CI | Clinical improvement |
| CT | Computed tomography |
| CR | Complete remission |
| CML | Chronic myelogenous leukemia |
| DLI | Donor lymphocyte infusion |
| DoMSR | Duration of Maintenance of at Least 35% Reduction in Spleen Volume |
| ELN | European LeukemiaNet |
| EMH | Extramedullary hematopoiesis |
| EPO | Erythropoietin |
| ESAs | Erythropoiesis stimulating agents |
| FTL3 | Fms-like tyrosine kinase 3 |
| GFR | Glomerular filtration rate test |
| HB | Hemoglobin |
| HMR | High molecular risk |
| HCT | Hematopoietic cell transplant |
| IFN | Interferon |
| INT | Intermediate |
| IPSS | International Prognostic Scoring System |
| IRs | Incidence rates |
| IWG-MRT | International Working Group for Myeloproliferative Neoplasms Research and Treatment |
| LFS | Leukemia-free survival |
| LCM | left costal margin |
| LMR | Low molecular risk |
| LV | Liver volume |
| MRI | Magnetic resonance imaging |
| MPN | Myeloproliferative neoplasm |
| MDS | Myeloysplastic dyndrome |
| MPL | Myeloproliferative leukemia virus oncogene |
| NRM | Non-relapse mortality |
| OS | Overall survival |
| PFS | Progression free survival |
| PLT | Platelet |
| PMF | Primary myelofirosis |
| PKC | Protein kinase C |
| PCR | Polymerase chain reaction |
| PV | Polycythemia vera |
| RIC | Reduced intensity conditioning |
| SAP | Statistical analysis plan |
| SV | Spleen volume |
| TRM | Transplant related mortality |
| TPO | Thrombopoietin |
| WBC | White blood cell count |
| WHO | World health organization |

# RESEARCH BACKGROUND AND RATIONALE

## Myelofibrosis and JAK Inhibitors

Myeloproliferative neoplasms (MPNs) are composed of a group of malignant tumors which mainly include primary myelofibrosis (PMF), chronic myeloid leukemia (CML), essential thrombocythemia (ET), polycythemia vera (PV), chronic neutrophilic leukemia (CNL), chronic eosinophilic leukemia (CEL), NOS (CEL-NOS) and systemic mastocytosis (SM). The diagnosis of chronic myeloid leukemia requires the presence of the Philadelphia chromosome t (9:22) or the formation of the fusion gene BCR-ABL. The pathogenesis of primary myelofibrosis (PMF), essential thrombocythemia (ET) and polycythemia vera (PV) is related to the mutation of the Janus kinase (referred to as JAK).

Myelofibrosis (MF) is one of MPNs. When MF occurs on its own, it is known as primary myelofibrosis (PMF). It can also develop in individuals who have had other MPNs (known as secondary MF), including post polycythaemia vera MF (Post-PV-MF) and post essential thrombocythemia MF (Post-ET-MF).

Primary myelofibrosis (PMF), also known as chronic idiopathic myelofibrosis, is a BCR-ABL negative myeloproliferative disease. PMF is characterized by marrow fibrosis and extramedullary hematopoiesis, but the degree of MF is not related to the range of extramedullary hematopoiesis. The age of onset is mostly between 50-70 years old. The onset of the disease is slow. In the early stage, the patients have atypical clinical manifestation, and most of them even have no symptom. The middle stage symptom of the disease may include fatigue, weight loss or feeling of abdominal distension caused by splenomegaly. Almost all patients have splenomegaly. The laboratory test results indicate that most patients have different degrees of anemia (generally normocytic normochromic anemias) upon visit, and teardrop-shaped erythrocytes and polychromatic erythrocytes can be seen on mature red blood cells. Reticulocytes may increase slightly between 2%-5%. About 70% of patients present with myelocytes and erythroblasts, as some of characteristic features of the disease. The diagnostic basis of this disease is that marrow cannot be aspirated, and lots of reticular fibers can be observed in the biopsy sample. The chromosome examination shows that half of patients have chromosome abnormality, but the Philadelphia chromosome is not involved. MF has the poorest survival time and quality of life among MPNs, with the median survival time of about 5 years. The survival time of high-risk patients is merely about 1-2 years. MF eventually progresses to bone marrow failure or transforms into acute leukemia (AL). The leading causes of death are bleeding, serious infections, and heart failure.

Since the role of the JAK2 gene in the onset of BCR-ABL-negative MPNs was first reported in 2005, JAK2 V617F mutation and MPNs have become a hot research topic. The JAK2 V617F mutation refers to a G to T missense mutation at nucleotide 1849 of exon 12, leading to substitution of phenylalanine for valine at codon 617 at the JAK pseudokinase domain (JH2). The WHO has now included the presence of JAK2 V617F mutation in the main diagnostic criteria for PMF.

The current treatments for MF include conventional medication, radiotherapy, investigational drugs, and hematopoietic stem cell transplantation (HSCT). The therapeutic strategy can be selected based on prognosis grouping. The only means that may cure PMF is allogeneic HSCT (allo-HSCT). However, the failure rate of allo-HSCT is high, and the incidence rate of postoperative complications (chronic graft versus host disease, infection, etc.) and the death rate are high as well. In addition, there is no conclusive evidence confirming that allo-HSCT is related to prolonged survival.

NCB018424 (Jakafi, Ruxolitinib) is a JAK1 and JAK2 inhibitor with high selectivity and oral bioavailability. It has been approved in the USA and the EU for the treatment of adult patients with MF including primary myelofibrosis, post-polycythemia vera myelofibrosis and post-essential thrombocythemia myelofibrosis, and for the treatment of patients with polycythemia vera (PV) who have had an inadequate response to or are intolerant of hydroxyurea.

The efficacy and safety of Ruxolitinib have been confirmed by Phase I/II clinical trials and two key Phase III clinical trials (COMFORT-I and COMFORT-II). In the Phase I/II trial, 153 MF patients (including PMF, post-PV MF and post-ET MF patients) were treated with ruxolitinib, and 65.4% of the patients were high-risk. Within 3 months of treatment, 44% of patients had a
> 50% reduction in spleen volume, and there was no significant difference in efficacy between patients with and without JAK2 mutations. In 2011, Incyte published results from the COMFORT-I and COMFORT-II trials which evaluated the safety and efficacy of ruxolitinib in the treatment of 528 patients with MF (including PMF, post-PV MF, and post-ET MF). Results: In COMFORT-I, 41.9% of patients treated with ruxolitinib had a ≥ 35% reduction in spleen volume, v.s. only 0.7% of patients in the placebo group (*p* < 0.001). In COMFORT-II, 28% of patients in the ruxolitinib group had a ≥ 35% reduction in spleen volume at week 48 v.s. 0% of patients in the control (BAT) group. Almost all patients treated with ruxolitinib achieved various degrees of reduction in SV, while almost all patients treated with placebo and more than 50% patients treated with BAT had an increase in SV. The patients’ medical records indicated that ruxolitinib can effectively reduce SV regardless of JAK2 mutation, sex, age, IPSS risk category or baseline SV. Compared with the placebo group, most patients treated with ruxolitinib experienced a > 50% reduction in MF-related symptoms (including abdominal discomfort, night sweats, pruritus, and muscle and bone pain) and an improved quality of life. A total of 32.4% of patients treated with ruxolitinib felt their condition had improved greatly, while most patients in the placebo group felt their condition was either unchanged or worsened. In COMFORT-I, ruxolitinib significantly reduced mortality rate when compared with placebo. It has been proven that ruxolitinib can reduce systemic symptoms and splenomegaly caused by MF. Ruxolitinib was approved by the FDA for the treatment of MF in November 2011, and was approved for marketing in Canada in July 2012 and in Europe in August 2012.

In addition to studies on myelofibrosis, an international, multi-center, randomized, open-label, phase III clinical trial was conducted comparing ruxolitinib with standard therapy in patients with polycythemia vera (the RESPONSE trial). The objective of the trial was to evaluate the efficacy of ruxolitinib in preventing thrombotic events, reducing the spleen volume, and minimizing the risk of transformation to post-polycythemia vera myelofibrosis or acute myeloid leukemia (AML). A total of 232 patients meeting the inclusion criteria were randomly divided to the ruxolitinib treatment group (110 cases) and the standard treatment group (112 cases). The primary endpoint was the percentage of patients achieving hematocrit control and decrease in spleen volume of ≥ 35% after 32 weeks of treatment.

Results from the RESPONSE trial showed that the primary end point was achieved in 21% of the patients in the ruxolitinib group versus 1% of those in the standard-therapy group (*p* < 0.001). The percentages of patients achieving hematocrit control in the ruxolitinib group and the standard treatment group were 60% and 20%, respectively; and 38% and 1% of patients achieved the ≥ 35% decrease in spleen volume in the above two groups. A complete hematologic remission was achieved in 24% of patients in the ruxolitinib group and 9% of those in the standard-therapy group (*p* = 0.003); 49% versus 5% had at least a 50% reduction in the total symptom score at week 32. The main side effects observed in the ruxolitinib group were that 2% of patients presented with anemia (Grade 3 or 4) and 5% presented with thrombocytopenia (Grade 3 or 4), while the percentages in the control group were 0% and 4%, respectively. Herpes zoster infection was reported in 6% of patients in the ruxolitinib group and 0% of those in the standard-therapy group (Grade 1 or 2 in all cases). Thrombus occurred in 1 patient of the ruxolitinib group and 6 patients of the standard treatment group.

Based on the RESPONSE trial, the FDA approved ruxolitinib for the treatment of patients with polycythemia vera who have had an inadequate response to or are intolerant of hydroxyurea in December 2014, making ruxolitinib the first drug approved for the treatment of polycythemia vera.

## Clinical Data on Momelotinib

Momelotinib (CYT387) is a potent JAK1/JAK2 inhibitor developed by Gilead Sciences. The drug is currently undergoing phase III clinical trials. Jaktinib is a novel deuterated compound derived from the chemical structure of momelotinib. The first human trial of momelotinib was carried out in patients with myelofibrosis. The trial included a dose escalation phase, a dose expansion phase, and a maintenance treatment phase. The maximum dosage of a single dose of momelotinib was 800 mg q.d.; and the maximum dosage of repeated doses of momelotinib was 250 mg b.i.d. for at least 6 cycles. Results from a phase 1/2 clinical trial involving 166 patients showed that the DLT was reached at 400 mg q.d. and the MTD was 300 mg q.d. Table 1 summarizes the safety data of momelotinib observed in the phase 1/2 clinical trial.

**Table 1. Treatment-related AEs after administration of different doses of momelotinib to MF patients for 9 months**

| **Adverse events (AE) (N = 166)** | **Grade 1** | **Grade 2** | **Grade 3** | **Grade 4** |
| --- | --- | --- | --- | --- |
| Circulatory System-Related AE |  |  |  |  |
| Anemia | 2% | 2% | 2% | 0% |
| Leukopenia | 2% | <1% | 0% | <1% |
| Neutropenia | <1% | 2% | 1% | 2% |
| Thrombocytopenia  Baseline Platelet > 150 × 10^9^/L (N = 97) | 14%  18% | 8%  1% | 17%  9% | 7%  1% |
| Non-Circulatory AE (≥ 10%) |  |  |  |  |
| Diarrhea | 18% | 4% | 0% | 0% |
| Vertigo | 22% | 1% | 0% | 0% |
| Headache | 14% | 0% | 1% | 0% |
| Nausea | 20% | <1% | 0% | 0% |
| Peripheral Neuropathy | 25% | 2% | 0% | 0% |
| Non-Hematologic Lab Abnormality (≥ 5%) |  |  |  |  |
| ALP increased | 5% | 1% | 1% | 0% |
| ALT increased | 9% | 3% | 2% | 0% |
| Amylase increased | 7% | 2% | 0% | 0% |
| AST increased | 9% | 2% | 1% | 0% |
| Bilirubin increased | 7% | 2% | 0% | 0% |
| Creatinine increased | 5% | 3% | 0% | 0% |
| Lipase increased | 8% | <1% | 4% | 0% |

Note: includes different dosages (100, 150, 200, 300 and 400 mg) and dosing frequency (q.d. and b.i.d.).

Momelotinib showed good preliminary efficacy in patients with myelofibrosis. Table 2 shows the responses to momelotinib in spleen. Some disease symptoms were significantly controlled, including fever (100%), night sweats (57%), pruritus (52%), bone pain (33%), and cough (29%).

**Table 2. Changes in spleen response and volume in patients treated with momelotinib in the phase 1/2 clinical trial**

| **Dosage** | **150mg qd**  **(n=52)** | **300mg qd**  **(n=60)** | **150mg bid**  **(n=42)** | **Total**  **(n=166)** |
| --- | --- | --- | --- | --- |
| Evaluable Spleen | 47 | 51 | 37 | 145 |
| Spleen Response (IWG-MRT) | 32% | 39% | 38% | 37% |
| Average Reduction in Spleen Volume at Month 6 | -36% | -38% | -46% | -38% |

Among the JAK inhibitors currently under development, momelotinib has been known for its ability to improve anemia. 59% of patients experienced improvement of anemia, and 70% of blood transfusion-dependent patients (N = 33) were blood transfusion-independent for at least 12 weeks (Table 3).

**Table 3. Blood transfusion independence in patients treated with momelotinib in the phase 1/2 clinical trial**

| **Dosage** | **150mg qd**  **(n=52)** | **300mg qd**  **(n=60)** | **150mg bid**  **(n=42)** | **Total**  **(n=166)** |
| --- | --- | --- | --- | --- |
| Evaluable Transfusion-Dependent Baseline | 24 | 28 | 14 | 68 |
| Transfusion Independence Rate (at W12) | 63% | 75% | 57% | 68% |
| Hemoglobin Elevation of 2 g/dL or Above (at W8) | 11% | 8% | 14% | 13% |
| IWG-MRT Anemia Response Rate | 48% | 55% | 36% | 48% |

In the extension study of momelotinib, 58 (39%) patients achieved a spleen response meeting IWG-MRT criteria, with the median duration of response being 785 days. 59 patients (53 %) achieved anemia response with a median maintenance duration of 1,042 days. 30% of patients experienced grade 3 or 4 thrombocytopenia and “first-dose response" of transient dizziness (16 cases, 10%) and hypotension (8 cases, 5%).

In a paper published by RA Abdelrahman et al. from Mayo Clinic, the treatment efficacy of ruxolitinib in 51 MF patients enrolled in a pivotal phase 3 clinical trial at the Mayo Clinic was compared with momelotinib in 60 patients enrolled in the phase 1/2 study (Table 3). The study results showed that neither treatment group achieved complete response (CR); one patient in the momelotinib group achieved partial response (PR), but none in the ruxolitinib group did so. Around 57% of patients in the momelotinib group versus 18% of patients in the ruxolitinib group achieved symptom improvement as per the 2013 IWG-MRT-ELN response criteria. The spleen response rate was 42% in the momelotinib group versus 15% in the ruxolitinib group; The anemia response rate was 45% in the momelotinib group versus 11% in the roxuolitinib group. The comparative results of efficacy between ruxolitinib and momelotinib are listed in Table 4 as follows.

One thing in particular is that although all patient data in this study were from Mayo Clinic, the comparative study was a retrospective analysis rather than a randomized controlled trial of ruxolitinib and momelotinib. Thus the study only suggested that momelotinib may possibly have better efficacy and a greater symptom improvement rate. Based on results of this study and other phase 1/2 clinical studies, momelotinib is currently undergoing two phase 3 clinical trials. One is a randomized, open-label trial comparing momelotinib versus best available therapy, and the other is a double-blind, double dummy, head-to-head comparative study of momelotinib and ruxolitinib.

**Table 4. Efficacy comparison of momelotinib and ruxolitinib
(as per 2013 IWG-MRT criteria)**

| Efficacy Endpoints | Momelotinib Group (N = 60) | Ruxolitinib Group (N = 51) |
| --- | --- | --- |
| CR, n (%) | 0 (0%) | 0 (0%) |
| PR, n (%) | 1 (1.7%) | 0 (0%) |
| Clinical Improvement (CI), ^a^ n (%) | 34 (57%) | 9 (18%) |
| Anemia response, n evaluable (n response; %) | 42 (19; 45%) | 19 (2; 11%) |
| Anemic Response in Patients With Blood Transfusion Dependence,  n evaluable (n response; %) | 32 (17; 53%) | 12 (2; 17%) |
| Spleen response, n evaluable (n response; %) | 57 (24; 42%) | 46 (7; 15%) |
| Median Duration of Treatment, Month (Range) | 26 (3-47+) | 12 (1-68) |

## Clinical Data on Momelotinib B.I.D.

The efficacy and safety of momelotinib b.i.d. was evaluated in a multi-center, open-label, phase 1/2 clinical trial. A total of 61 patients with moderate to high risk myelofibrosis were enrolled. The first phase of the trial explored the DLT and MTD of momelotinib b.i.d. regimen, with a starting dose of 200 mg b.i.d. and a treatment duration of 28 days. The study escalated to the next dose group once DLT was observed in ≤ 2 out of 6 subjects. The second phase of the trial was the dose expansion phase. Subjects were given the MTD or one dosage-level lower, twice daily, with 28 days per treatment cycle for a total of 6 cycles.

In the first phase of the trial, 6 and 7 subjects will be enrolled into the 200 mg b.i.d. group and the 250 mg b.i.d. group, respectively. Although no protocol-defined DLT was observed, treatment interruption occurred in 6 out of 7 subjects in the 250 mg b.i.d. group, resulting in dose reduction in 5 subjects and treatment discontinuation in 1 subject. Thus, the Safety Review Committee chose 200 mg b.i.d. as the dosage for dose expansion phase of the trial. A total of 48 new subjects were enrolled in the second phase of the trial. A total of 45 (73.8%) subjects completed 6 treatment cycles of the b.i.d. regimen. Twenty-two cases were treated for at least 2 years, after which they were enrolled in a open-label maintenance study. The median duration of exposure to the investigational drug was 382 days (2–995 days).

All 61 subjects experienced at least 1 adverse event, including 58 subjects with TRAEs. 33 subjects had a serious adverse event at least once, including 14 subjects who reported drug-related serious adverse events. The most common adverse events reported during treatment were non-hematologic, including: diarrhea (28 cases), peripheral neuropathy (27 cases), dizziness (22 cases), and hypotension (15 cases). The majority of these were Grade 1-2 adverse events. Figure 1a summarizes the adverse events experienced during treatment. The most common hematological toxicity (Figure 1b) was thrombocytopenia, which occurred in 24 subjects. Grade ≥ 3 toxicities occurred in 18 subjects and were more common in the 250 mg b.i.d. group (5 out of 7 subjects). Although no subject discontinued the study due to thrombocytopenia, there were 3 SAEs of Grade 4 related to momelotinib, all of which were resolved after drug interruption.


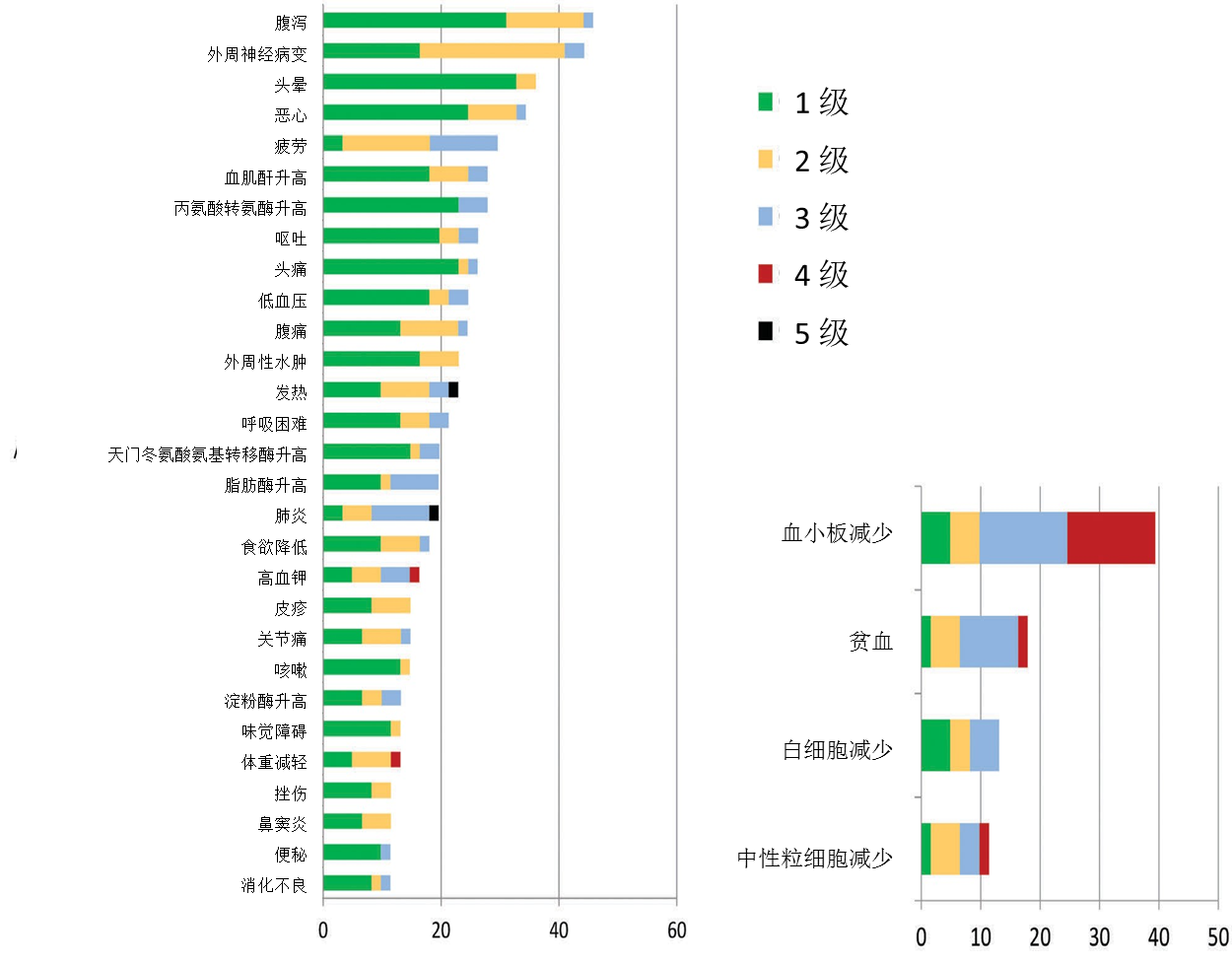


Thrombocytopenia

Anemia

Leukopenia

Neutropenia

Diarrhea

Peripheral Neuropathy

Dizziness

Nausea

Fatigue

Blood creatinine increased

Alanine aminotransferase increased

Vomiting

Headache

Hypotension

Abdominal pain

Peripheral edema

Pyrexia

Dyspnea

Aspartate aminotransferase increased

Lipase increased

Pneumonitis

Appetite decreased

Hyperkalemia

Rash

Arthralgia

Cough

Amylase increased

Dysgeusia

Weight decreased

Contusion

Sinusitis

Constipation

Dyspepsia

Grade 1

Grade 2

Grade 3

Grade 4

Grade 5

| **Figure 1a. Treatment-emergent adverse events** | **Figure 1b. Hematological toxicities** |
| --- | --- |

The modified intention to treat (mITT) population consists of 60 subjects, including 53 in the 200 mg b.i.d. group and 7 in the 250 mg b.i.d. group. Refer to the following table (Table 5) for spleen response summary of momelotinib b.i.d. regimen.

**Table 5. Spleen responses from momelotinib b.i.d. regimen (200 mg and 250 mg)**

| **Treatment Response** | **Result** |
| --- | --- |
| Received at least one dose of momelotinib and have at least one efficacy assessment | 60 (100%) |
| **Spleen Response** |  |
| **Spleen Response by Physical Examination** |  |
| Baseline palpable spleen > 5 cm | 50 (83.3%) |
| ≥ 50% reduction in palpable spleen lasting ≥ 8 weeks for baseline palpable spleen ≥ 10 cm: A (N = 41) | 28 (68.3%) |
| Resolution of palpable splenomegaly that lasts ≥8 weeks for baseline splenomegaly > 5 and < 10 cm  weeks for baseline splenomegaly: B (n=9) | 8 (88.9%) |
| Spleen Response: A + B | 36 (72.0%) |
| Median time to onset of spleen response (minimum, maximum) (N = 36) | 49.5 days (13,512) |
| Median duration of spleen response (minimum, maximum) (N = 36) | 249.5 days (71,976) |
| **Spleen Response by MRI** |  |
| Baseline MRI | 59 |
| ≥ 35% reduction in spleen volume measured by MRI at W24 | 27 (45.8%) |
| Baseline MRI of patients with palpable splenomegaly at baseline (> 5 cm) | 50 |
| ≥ 35% reduction in spleen volume measured by MRI at W24 | 27 (56.2%) |

The objective of this trial was to evaluate the safety and tolerability of momelotinib b.i.d. regimen, and to obtain preliminary efficacy results of momelotinib b.i.d. regimen in the treatment of patients with myelofibrosis. The b.i.d. regimen allowed a higher total daily dose of momelotinib compared with the q.d. regimen. No additional safety signals were observed in the high-dose b.i.d. regimen, and most subjects (73.8%) completed the first 6 treatment cycles of momelotinib b.i.d. regimen.

## Jaktinib Dose Escalation Study

The study on Jaktinib hydrochloride tablets in healthy subjects has been completed. This is a single-center, randomized, double-blind, placebo-controlled, single- and multiple-dose, dose-escalation study evaluating the effects of food on pharmacokinetics and metabolic transformation of Jaktinib. The objective is to evaluate the tolerability, pharmacokinetic characteristics, metabolic transformation, and effects of food on the pharmacokinetics of single- and multiple-dose Jaktinib in healthy subjects. The trial is divided into 3 phases: single ascending dose, multiple ascending dose, and effects of food on pharmacokinetics and metabolic transformation.

**Single Ascending Dose (SAD)**

This study included 8 dose groups: 25 mg, 50 mg, 100 mg, 150 mg, 200 mg, 250 mg, 300 mg, and 400 mg. Each group enrolled eight subjects, including six who received the investigational drug and two who received placebo. The male to female ratio was close to 1:1. Subject of each group received a single dose on an empty stomach, and underwent a tolerability assessment on D3. A dosage group underwent testing only if the previous dosage was well tolerated.

Safety results:

The number and percentage of subjects in each dose group who reported treatment-emergent adverse events (TEAE) were: 1 (16.7%) subject in the 25 group, none in the 50 mg group, 1 (16.7%) subject in the 100 mg group, 4 (66.7%) subjects in the 150 mg group, 2 (33.3%) subjects in the 200 mg group, 1 (16.7%) subject in the 250 mg group, 5 (83.3%) subjects in the 300 mg group, and 1 (16.7%) subject in the 400 mg group. TEAE was reported in 3 (18.8%) subjects in the placebo group. All reported AEs were related to the investigational drug. No SAE was reported. There was no TEAE resulting in study withdrawal. The incidence of adverse events did not show obvious dose dependence.

The most common TEAE in the SAD study was headache, which was reported by 5 subjects (1 (16.7%) subject in 250 mg group and 4 (66.7%) subjects in 300 mg group). Headache was not reported in the placebo group. No more than 10% of subjects reported any other TEAE besides headache. The most common TEAE in the placebo group was supraventricular arrhythmia (2 subjects, 12.5%), which was not reported by subjects who received the investigational drug.

No CTCAE Grade ≥ 4 TEAE occurred during the treatment period. Only 1 out of the 48 subjects who received the investigational drug in the SAD study reported a CTCAE grade ≥ 3 TEAE. The subject was in the 250 mg dose group (16.7%). The adverse event was chicken pox, which was possibly related to the investigational drug in the opinion of the investigator. The AE occurred on Day 5 of administration after the subject (S080) completed the tolerance assessment on Day 3 and withdrew from the study. All other TEAEs were Grade 1 events.

**Multiple Ascending Dose (MAD)**

This study included 5 dose groups: 100 mg q24h, 150 mg q24h, 100 mg q12h, 200 mg q24h, and 150 mg q12h (groups 9-13). Each group enrolled ten subjects, including eight who received the investigational drug and two who received placebo. The male to female ratio was close to 1:1. Subjects of each group received 10 or 19 doses on an empty stomach, and underwent tolerability assessments on D4, D7, and D12. A dosage group underwent testing only if the previous dosage was well tolerated.

Safety results:

The number and percentage of subjects in each dose group who reported TEAEs were: 7 (87.5%) subjects in the 100 q24h group, 7 (87.5%) subjects in the 150 q24h group, 7 (87.5%) subjects in the 100 q12h group, 7 (87.5%) subjects in the 200 q24h group, and 5 (62.5%) subjects in the 150 mg q12h group. TEAE was reported in 5 (50.0%) subjects in the placebo group. Most AEs reported in the MAD study were related to the investigational drug. No SAE was reported. Five subjects experienced TEAE leading to study withdrawal (1 in the 100 mg q12h group and 4 in the 150 mg q12h group). The incidence of adverse events did not show obvious dose dependence.

The most common TEAE in the MAD study was neutrophil count decreased, which was reported by 11 subjects (5 (62.5%) in the 100 mg q12h group, 1 (12.5%) in the 200 mg q24h group, and 5 (62.5%) in the 150 mg q12h group). Other TEAEs with an incidence of ≥ 10% (by PT) were:

• Diarrhea (9 subjects: 5 (62.5%) in the 150 mg q24h group, 3 (37.5%) in the 100 mg q12h group, and 1 (12.5%) in the 200 mg q24h group)

• Vertigo (7 subjects: 1 (12.5%) in 100 mg q24h group and 6 (75.0%) in the 100 mg q12h group); White blood cell count decreased (5 subjects: 1 (12.5%) in the 100 mg q12h group, 1 (12.5%) in the 200 mg q24h group, and 3 (37.5%) in the 150 mg q12h group)

• Sinus bradycardia (4 subjects: 3 (37.5%) in the 100 mg q24h group and 1 (12.5%) in the 150 mg q24h group).

The most common TEAEs (PT ≥ 10%) in the placebo group were vertigo (2 subjects, 20.0%), urinary tract infection (2 subjects, 20.0%), and anemia (2 subjects, 20.0%).

No CTCAE Grade ≥ 4 TEAE occurred during the treatment period. CTCAE Grade ≥ 3 TEAEs occurred in 6 out of the 40 subjects who received the investigational drug in the MAD study, including 1 (12.5%) in the 100 mg q12h group and 5 (62.5%) in the 150 mg q12h group. All these TEAEs were neutrophil count decreased. All CTCAE grade ≥ 3 TEAEs were determined by the investigator to be related to the investigational drug. With the exception of CTCAE grade ≥3 AEs, most AEs experienced by subjects who were given the investigational drug were grade 1 and a few were grade 2 (7 subjects experienced grade 2 AEs and 31 subjects experienced grade 1 AEs). Overall, in the MAD trial, the frequencies of more severe TEAEs were slightly higher in subjects receiving q12h regimen than those receiving q24h regimen.

**Study on effects of food on pharmacokinetics and metabolic transformation**

There were 12 subjects in total, with a male to female ratio close to 1:1, who were randomly allocated to Group A and Group B, with 6 subjects in each group. Subjects received 200 mg Jaktinib or MTD observed during the MAD study. Group A received Jaktinib on an empty stomach in Cycle 1, and after a washout period, they crossed over to receive Jaktinib after meals in Cycle 2. Group B received Jaktinib after meals in Cycle 1, and after a washout period, they crossed over to receive Jaktinib on an empty stomach in Cycle 2. The washout period between the two cycles was around 5 days. PK urine and fecal samples were collected in Group A during Cycle 1 under the fasting conditions. Subjects withdrew from the study after completing the tolerability assessment on D8.

Safety results:

In the food effect study, TEAEs occurred in 3 subjects (25.0%) post-administration on an empty stomach and 5 subjects (41.7%) post-administration under fed conditions. All reported TEAEs were related to the investigational drug. No SAE was reported. There was no TEAE resulting in study withdrawal.

The most common TEAEs (PT ≥ 10%) in the food effect study were diarrhea (1 subject each under fasting and fed conditions), headache (2 subjects, both under fed conditions), and anemia (1 subject each under fasting and fed conditions).

No CTCAE Grade ≥ 4 TEAE occurred during the treatment period. In the food effect study, no CTCAE grade ≥ 3 TEAE occurred and only 1 (8.3%) subject experienced CTCAE grade 2 TEAE (anemia) and other reported TEAEs were all grade 1.

## Introduction to the Investigational Drug

Jaktinib hydrochloride tablets, with Jaktinib as the active ingredient, is an oral small molecule JAK kinase inhibitor, a class 1 new drug developed by Suzhou Zelgen Biopharmaceuticals Co., Ltd./Shanghai Zelgen Pharmaceutical Technology Co., Ltd. It is a novel compound derived from the chemical structure of momelotinib - a compound undergoing Phase III clinical trial by GILEAD - by replacing hydrogen atoms at specific positions with deuterium, and then screened through pharmacokinetic and pharmacodynamic studies.

### 1.5.1 Drug name and strength

Structural Formula:

Molecular Formula: C_23_H_22_D_4_Cl_2_N_6_O_3_

Molecular Weight: 509.42

Chinese Name: 杰克替尼二盐酸盐一水合物 (Tentative)

English Name: Jaktinib Dihydrochloride Monohydrate (Tentative)

*Chinese Chemical Name:* N-(氰基甲基)-4-(2-(4-(2’,2’,6’,6’*-d*_4_-吗啡啉基)苯基氨基)嘧啶-4-基)苯甲酰胺二盐酸盐一水合物

English Chemical Name: *N*-(cyanomethyl)-4-(2-(4-(2’,2’,6’,6’*-d*_4_-morpholinophenyl)amino)

pyrimidin-4-yl) benzamide Dihydrochloride Monohydrate

Compound Code: ZG0128

Active Component: ZG0163, Jaktinib

CAS No.: 1619927-66-0 (Jaktinib, ZG0163)

Jaktinib hydrochloride tablets: 50 mg/tablet.

Jaktinib hydrochloride tablet is an orange-red film-coated tablet, yellow without the coating. The tablet is composed of the active pharmaceutical ingredient ZG0128 and inactive ingredients including microcrystalline cellulose (101), croscarmellose, hypromellose (E15), magnesium stearate, and film coating premix (295F630005 orange).

### 1.5.2 Type and mechanism of pharmacological action

Jaktinib is a broad-spectrum kinase inhibitor that inhibits JAK1, JAK2 and JAK3 in Janus kinase family of non-receptor tyrosine, as well as receptor tyrosine kinase III (RTK III), FMS-like tyrosine kinase 3 (FLT3), and c-Kit. JAK1, 2, and 3 mediate several cytokines and growth factor signaling pathways which are important to hematopoietic, inflammatory, and immune functions. JAK signaling pathways are involved in recruiting and activating STAT (Signal Transducers and Activators of Transcription) factors into cytokine receptors. The activated STAT factors will be located in nucleus to regulate the expression of downstream genes.

Myelofibrosis is a type of myeloproliferative neoplasms known to be associated with dysregulation of JAK1 and JAK2 signaling. In JAK2V617F bone marrow transplant mouse and transgenic mouse models, oral administration of Jaktinib hydrochloride effectively improved the pathological characteristics of bone marrow hyperplasia, including a reduction in spleen volume, inhibition of abnormal cell proliferation, induction of mutant cell apoptosis, and prevention of myelofibrosis progression.

### 1.5.3 Theoretical basis of the study

Jaktinib hydrochloride has demonstrated good pharmacokinetic properties and pharmacological effects in preclinical pharmacokinetics and pharmacodynamics studies. Pre-clinical toxicology studies indicated that apart from the toxic and side effects associated with pharmacological effects, Jaktinib hydrochloride has clear target organs of toxicity and controllable safety.

Current data support the clinical development of Jaktinib hydrochloride to determine the pharmacokinetics, clinical efficacy, and safety of Jaktinib hydrochloride as a novel drug for the treatment of myeloproliferative neoplasms, especially patients with intermediate or high risk myelofibrosis.

This is a registered clinical trial. Pharmacokinetics and dose exploration studies will be carried out in patients with myelofibrosis including PMF, Post-PV-MF and Post-ET-MF, in order to evaluate the efficacy and safety of Jaktinib hydrochloride tablets in the treatment of myelofibrosis.

This trial was conducted in accordance with relevant principles found in the "Declaration of Helsinki", "The Drug Administration Law of the People's Republic of China", "Drug Registration Regulations", "Good Clinical Practice", and "Guidelines for Laboratory Management of Biological Sample Analysis in Clinical Drug Trials (Trial Version)".

The clinical trial objectives, methodology, organization, data analysis, and trial management are described below.

# TRIAL OBJECTIVES

- To evaluate the pharmacokinetic (PK) properties of Jaktinib hydrochloride tablets in patients with myelofibrosis;
- To evaluate the pharmacodynamic properties of Jaktinib hydrochloride tablets in patients with myelofibrosis;
- To evaluate the safety of Jaktinib hydrochloride tablets in the treatment of myelofibrosis;
- To evaluate the preliminary efficacy of Jaktinib hydrochloride tablets in the treatment of myelofibrosis.

# TRIAL DESIGN

## Overall Design

The objective of this trial is to evaluate the safety and efficacy of Jaktinib hydrochloride tablets in the treatment of patients with intermediate or high risk myelofibrosis, and to analyze the pharmacokinetic and pharmacodynamic properties in the study population.

This trial is a single-arm, open-label, multi-center, randomized, two-stage study. Around 100 subjects are expected to be enrolled at 25 study sites during Stage 1 and an additional 36 subjects are expected to be enrolled into the expansion cohort during Stage 2, i.e.approximately 136 subjects in total.

Two dose groups are set up for Stage 1, i.e., 100 mg b.i.d. and 200 mg q.d. The subjects are randomized in a 1:1 ratio, with 50 subjects per group for a total of 100 subjects. In addition to the 100 subjects randomized during Stage 1, an additional 36 subjects will be enrolled into the 100 mg b.i.d. expansion cohort during Stage 2 following a comprehensive assessment by the principal investigator and the sponsor.

Based on the interim analysis of ZGJAK002, Jaktinib 100 mg b.i.d. was slightly superior to 200 mg q.d. in spleen response (Table 6), symptom improvement (Table 7 and Figure 2), and anemia improvement (Table 8 and Figure 3). There was also no significant difference in tolerability between the two regimens (Tables 9 and 10). Moreover, daily exposure (AUC_0-24_) of the parent drug and metabolites of Jaktinib were comparable between the 200 mg q.d. and 100 mg b.i.d. regimens after the first dose. Exposure of the metabolite ZG0244 was comparable to that of the parent drug, while exposure of the metabolite ZG0245 was low (Table 11). In summary, after a comprehensive assessment of risks and benefits, the additional 36 subjects will all be enrolled into the 100 mg b.i.d. group.

**Table 6. Spleen response at week 24 in ZGJAK002 trial of Jaktinib hydrochloride tablets**

| **Data Set** | **Dose group** | **Spleen Response** | **Spleen Non-Responder** |
| --- | --- | --- | --- |
| ITT | 100mg bid(N=23) | 12(52.17%) | 11(47.83%) |
|  |  | [26.02%,73.18%] | |
|  | 200mg qd(N=20) | 7(35.00%) | 13(65.00) |
|  |  | [15.39%,59.22%] | |
|  | Total (N = 43) | 19(44.19%) | 24(55.81%) |
|  |  | [29.08%,60.12%] | |

**Table 7. Changes in MPN-MF-TSS in ZGJAK002 trial of Jaktinib hydrochloride tablets (ITT)**

| Dose group | Baseline  (N = 104) | Week 6  (N = 86) | Week 12  (N = 66) | Week 18  (N = 54) | Week 24  (N = 23) |
| --- | --- | --- | --- | --- | --- |
| 100mg bid | 30.83±20.63 | 16.27±16.17 | 13.62±14.8 | 11.41±13.55 | 10.71±14.94 |
| 200mg qd | 21.31±12.89 | 14.2±14.13 | 12.52±10.30 | 11.6±10.60 | 8.22±5.97 |


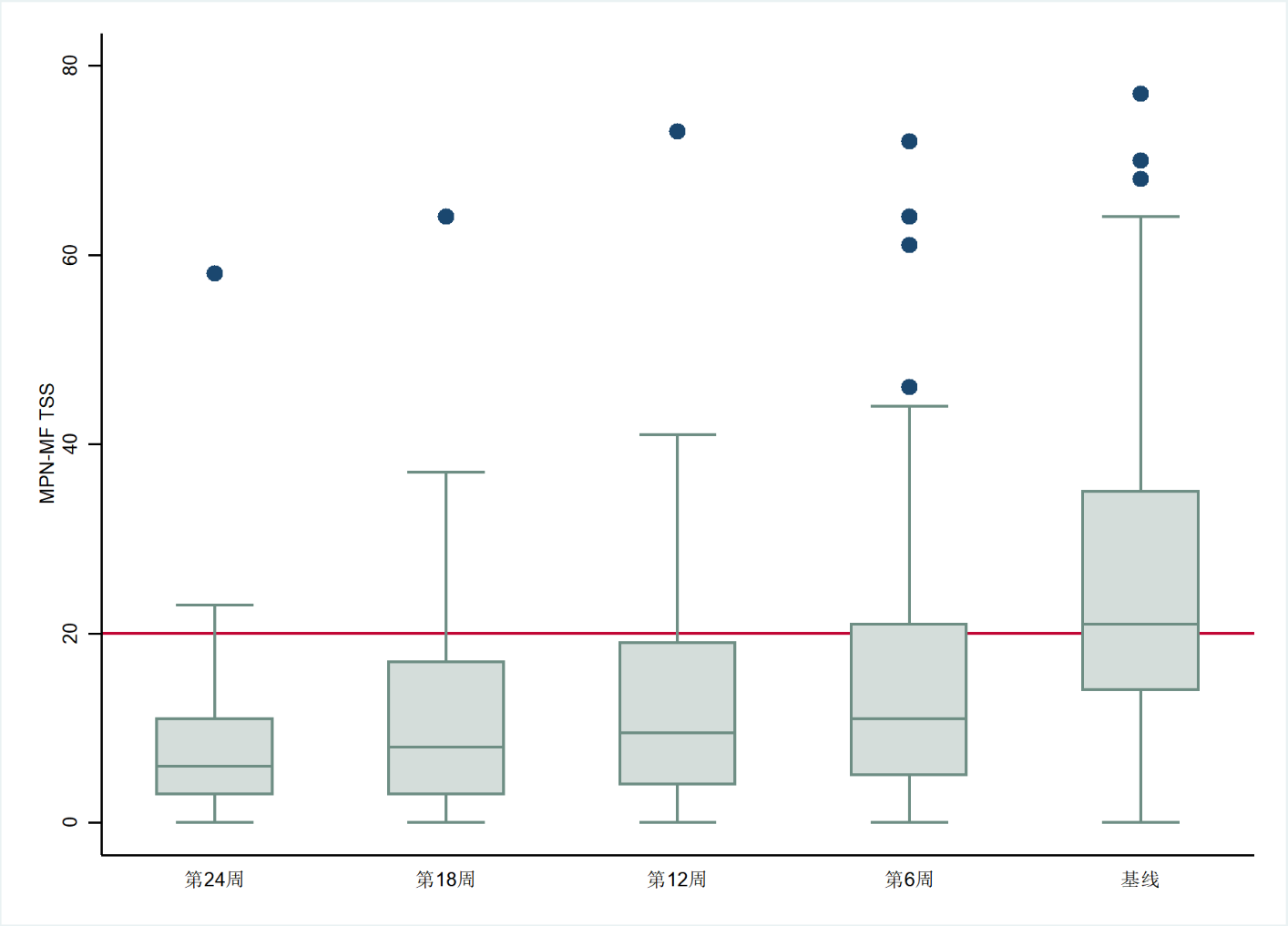


Baseline

Week 24

Week 18

Week 12

Week 6

**Figure 2. Changes in MPN-MF-TSS in ZGJAK002 trial of Jaktinib hydrochloride tablets (ITT)**

**Table 8. Changes in hemoglobin in ZGJAK002 trial of Jaktinib hydrochloride tablets (ITT)**

| Dose group | Baseline | Week 6 | Week 12 | Week 18 | Week 24 |
| --- | --- | --- | --- | --- | --- |
|  | n=104 | n=90 | n=67 | n=54 | n=37 |
| 100mg bid(g/L) | 112.38±30.35 | 114.81±27.43 | 111.91±26.28 | 114.14±27.13 | 118.20±25.96 |
| 200mg qd(g/L) | 101.96±26.88 | 102.59±27.93 | 104.94±22.39 | 111.58±23.58 | 110.82±25.57 |
| Total | 107.17±29.00 | 108.29±28.22 | 108.58±24.57 | 112.95±25.35 | 114.81±25.69 |


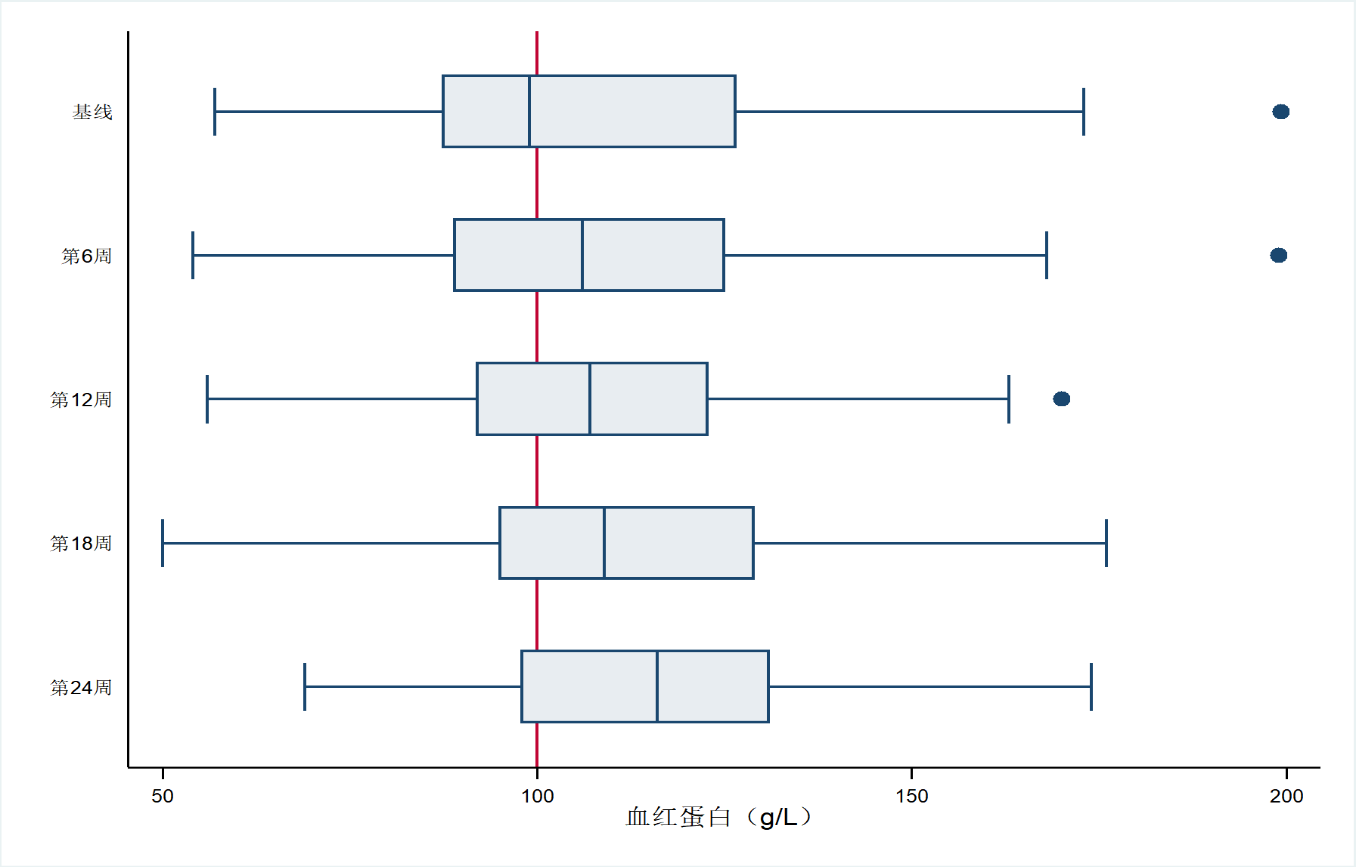


Baseline

Week 6

Week 12

Week 18

Week 24

Hemoglobin (g/L)

**Figure 3. Changes in hemoglobin in ZGJAK002 trial of Jaktinib hydrochloride tablets**

**Table 9. Overall adverse reactions in ZGJAK002 trial of Jaktinib hydrochloride tablets**

| **Adverse reaction** | **100mg bid (n=49)** | | | | | | | | | | **200mg qd (n=50)** | | | | | | | | | | **Total (n = 99)** | | | | | | | | | |
| --- | --- | --- | --- | --- | --- | --- | --- | --- | --- | --- | --- | --- | --- | --- | --- | --- | --- | --- | --- | --- | --- | --- | --- | --- | --- | --- | --- | --- | --- | --- |
|  | **Grade 1** | | **Grade 2** | | **Grade 3** | | **Grade 4** | | **Total** | | **Grade 1** | | **Grade 2** | | **Grade 3** | | **Grade 4** | | **Total** | | **Grade 1** | | **Grade 2** | | **Grade 3** | | **Grade 4** | | **Total** | |
|  | n | % | n | % | n | % | n | % | n | % | n | % | n | % | n | % | n | % | n | % | n | % | n | % | n | % | n | % | n | % |
| Overall treatment-emergent adverse reactions | 13 | 26.5 | 8 | 16.3 | 10 | 20.4 | 3 | 6.1 | 34 | 69.4 | 14 | 28.0 | 12 | 24.0 | 8 | 16.0 | 3 | 6.0 | 37 | 74.0 | 27 | 27.3 | 20 | 20.2 | 18 | 18.2 | 6 | 6.1 | 71 | 71.7 |
| Platelet count decreased | 5 | 10.2 | 6 | 12.2 | 4 | 8.2 | 2 | 4.1 | 17 | 34.7 | 4 | 8.0 | 3 | 6.0 | 4 | 8.0 | 1 | 2.0 | 12 | 24.0 | 9 | 9.1 | 9 | 9.1 | 8 | 8.1 | 3 | 3.0 | 29 | 29.3 |
| Anemia | 3 | 6.1 | 3 | 6.1 | 3 | 6.1 | 0 | 0.0 | 9 | 18.4 | 4 | 8.0 | 4 | 8.0 | 7 | 14.0 | 0 | 0.0 | 15 | 30.0 | 7 | 7.1 | 7 | 7.1 | 10 | 10.1 | 0 | 0.0 | 24 | 24.2 |
| Neutrophil count decreased | 1 | 2.0 | 1 | 2.0 | 2 | 4.1 | 0 | 0.0 | 4 | 8.2 | 2 | 4.0 | 2 | 4.0 | 2 | 4.0 | 1 | 2.0 | 7 | 14.0 | 3 | 3.0 | 3 | 3.0 | 4 | 4.0 | 1 | 1.0 | 11 | 11.1 |
| White blood cell count decreased | 0 | 0.0 | 3 | 6.1 | 0 | 0.0 | 0 | 0.0 | 3 | 6.1 | 0 | 0.0 | 5 | 10.0 | 1 | 2.0 | 1 | 2.0 | 7 | 14.0 | 0 | 0.0 | 8 | 8.1 | 1 | 1.0 | 1 | 1.0 | 10 | 10.1 |
| Alanine aminotransferase increased | 4 | 8.2 | 0 | 0.0 | 0 | 0.0 | 0 | 0.0 | 4 | 8.2 | 4 | 8.0 | 0 | 0.0 | 0 | 0.0 | 1 | 2.0 | 5 | 10.0 | 8 | 8.1 | 0 | 0.0 | 0 | 0.0 | 1 | 1.0 | 9 | 9.1 |
| Gamma-glutamyltransferase increased | 2 | 4.1 | 0 | 0.0 | 0 | 0.0 | 0 | 0.0 | 2 | 4.1 | 4 | 8.0 | 0 | 0.0 | 0 | 0.0 | 1 | 2.0 | 5 | 10.0 | 6 | 6.1 | 0 | 0.0 | 0 | 0.0 | 1 | 1.0 | 7 | 7.1 |
| Blood bilirubin increased | 2 | 4.1 | 1 | 2.0 | 0 | 0.0 | 0 | 0.0 | 3 | 6.1 | 3 | 6.0 | 0 | 0.0 | 0 | 0.0 | 0 | 0.0 | 3 | 6.0 | 5 | 5.1 | 1 | 1.0 | 0 | 0.0 | 0 | 0.0 | 6 | 6.1 |
| Blood creatinine increased | 0 | 0.0 | 0 | 0.0 | 0 | 0.0 | 0 | 0.0 | 0 | 0.0 | 6 | 12.0 | 0 | 0.0 | 0 | 0.0 | 0 | 0.0 | 6 | 12.0 | 6 | 6.1 | 0 | 0.0 | 0 | 0.0 | 0 | 0.0 | 6 | 6.1 |
| Nausea | 2 | 4.1 | 1 | 2.0 | 0 | 0.0 | 0 | 0.0 | 3 | 6.1 | 2 | 4.0 | 1 | 2.0 | 0 | 0.0 | 0 | 0.0 | 3 | 6.0 | 4 | 4.0 | 2 | 2.0 | 0 | 0.0 | 0 | 0.0 | 6 | 6.1 |
| Dizziness | 2 | 4.1 | 1 | 2.0 | 0 | 0.0 | 0 | 0.0 | 3 | 6.1 | 3 | 6.0 | 0 | 0.0 | 0 | 0.0 | 0 | 0.0 | 3 | 6.0 | 5 | 5.1 | 1 | 1.0 | 0 | 0.0 | 0 | 0.0 | 6 | 6.1 |
| Aspartate aminotransferase increased | 1 | 2.0 | 0 | 0.0 | 0 | 0.0 | 0 | 0.0 | 1 | 2.0 | 4 | 8.0 | 0 | 0.0 | 0 | 0.0 | 0 | 0.0 | 4 | 8.0 | 5 | 5.1 | 0 | 0.0 | 0 | 0.0 | 0 | 0.0 | 5 | 5.1 |
| Diarrhea | 2 | 4.1 | 1 | 2.0 | 0 | 0.0 | 0 | 0.0 | 3 | 6.1 | 2 | 4.0 | 0 | 0.0 | 0 | 0.0 | 0 | 0.0 | 2 | 4.0 | 4 | 4.0 | 1 | 1.0 | 0 | 0.0 | 0 | 0.0 | 5 | 5.1 |
| Asthenia | 3 | 6.1 | 0 | 0.0 | 0 | 0.0 | 0 | 0.0 | 3 | 6.1 | 2 | 4.0 | 0 | 0.0 | 0 | 0.0 | 0 | 0.0 | 2 | 4.0 | 5 | 5.1 | 0 | 0.0 | 0 | 0.0 | 0 | 0.0 | 5 | 5.1 |

**Table 10. Adverse reactions (at week 24) in ZGJAK002 trial of Jaktinib hydrochloride tablets**

| Adverse reactions at week 24 | **100mg bid (n=23)** | | | | | | | | | | **200mg qd (n=20)** | | | | | | | | | | **Total (n = 43)** | | | | | | | | | |
| --- | --- | --- | --- | --- | --- | --- | --- | --- | --- | --- | --- | --- | --- | --- | --- | --- | --- | --- | --- | --- | --- | --- | --- | --- | --- | --- | --- | --- | --- | --- |
|  | **Grade 1** | | **Grade 2** | | **Grade 3** | | **Grade 4** | | **Total** | | **Grade 1** | | **Grade 2** | | **Grade 3** | | **Grade 4** | | **Total** | | **Grade 1** | | **Grade 2** | | **Grade 3** | | **Grade 4** | | **Total** | |
|  | n | % | n | % | n | % | n | % | n | % | n | % | n | % | n | % | n | % | n | % | n | % | n | % | n | % | n | % | n | % |
| Treatment-emergent adverse reactions at week 24 | 6 | 26.1 | 5 | 21.7 | 5 | 21.7 | 1 | 4.3 | 17 | 73.9 | 7 | 35.0 | 5 | 25.0 | 2 | 10.0 | 2 | 10.0 | 16 | 80.0 | 13 | 30.2 | 10 | 23.3 | 7 | 16.3 | 3 | 7.0 | 33 | 76.7 |
| Platelet count decreased | 2 | 8.7 | 3 | 13.0 | 2 | 8.7 | 1 | 4.3 | 8 | 34.8 | 3 | 15.0 | 1 | 5.0 | 1 | 5.0 | 0 | 0.0 | 5 | 25.0 | 5 | 11.6 | 4 | 9.3 | 3 | 7.0 | 1 | 2.3 | 13 | 30.2 |
| Anemia | 1 | 4.3 | 2 | 8.7 | 1 | 4.3 | 0 | 0.0 | 4 | 17.4 | 4 | 20.0 | 3.0 | 15.0 | 2 | 10.0 | 0 | 0.0 | 9 | 45.0 | 5 | 11.6 | 5 | 11.6 | 3 | 7.0 | 0 | 0.0 | 13 | 30.2 |
| Alanine aminotransferase increased | 4 | 17.4 | 0 | 0.0 | 0 | 0.0 | 0 | 0.0 | 4 | 17.4 | 3 | 15.0 | 0 | 0.0 | 0 | 0.0 | 0 | 0.0 | 3 | 15.0 | 7 | 16.3 | 0 | 0.0 | 0 | 0.0 | 0 | 0.0 | 7 | 16.3 |
| Neutrophil count decreased | 1 | 4.3 | 0 | 0.0 | 2 | 8.7 | 0 | 0.0 | 3 | 13.0 | 1 | 5.0 | 1.0 | 5.0 | 1 | 5.0 | 0 | 0.0 | 3 | 15.0 | 2 | 4.7 | 1 | 2.3 | 3 | 7.0 | 0 | 0.0 | 6 | 14.0 |
| White blood cell count decreased | 0 | 0.0 | 2 | 8.7 | 0 | 0.0 | 0 | 0.0 | 2 | 8.7 | 0 | 0.0 | 3.0 | 15.0 | 0 | 0.0 | 0 | 0.0 | 3 | 15.0 | 0 | 0.0 | 5 | 11.6 | 0 | 0.0 | 0 | 0.0 | 5 | 11.6 |
| Dizziness | 2 | 8.7 | 1 | 4.3 | 0 | 0.0 | 0 | 0.0 | 3 | 13.0 | 2 | 10.0 | 0.0 | 0.0 | 0 | 0.0 | 0 | 0.0 | 2 | 10.0 | 4 | 9.3 | 1 | 2.3 | 0 | 0.0 | 0 | 0.0 | 5 | 11.6 |
| Nausea | 1 | 4.3 | 0 | 0.0 | 0 | 0.0 | 0 | 0.0 | 1 | 4.3 | 2 | 10.0 | 1.0 | 5.0 | 0 | 0.0 | 0 | 0.0 | 3 | 15.0 | 3 | 7.0 | 1 | 2.3 | 0 | 0.0 | 0 | 0.0 | 4 | 9.3 |
| Asthenia | 3 | 13.0 | 0 | 0.0 | 0 | 13.0 | 0 | 0.0 | 3 | 13.0 | 1 | 5.0 | 0 | 0.0 | 0 | 0.0 | 0 | 0.0 | 1 | 5.0 | 4 | 9.3 | 0 | 0.0 | 0 | 0.0 | 0 | 0.0 | 4 | 9.3 |
| Aspartate aminotransferase increased | 1 | 4.3 | 0 | 0.0 | 0 | 0.0 | 0 | 0.0 | 1 | 4.3 | 2 | 10.0 | 0.0 | 0.0 | 0 | 0.0 | 0 | 0.0 | 2 | 10.0 | 3 | 7.0 | 0 | 0.0 | 0 | 0.0 | 0 | 0.0 | 3 | 7.0 |
| Blood creatinine increased | 0 | 0.0 | 0 | 0.0 | 0 | 0.0 | 0 | 0.0 | 0 | 0.0 | 3 | 15.0 | 0.0 | 0.0 | 0 | 0.0 | 0 | 0.0 | 3 | 15.0 | 3 | 7.0 | 0 | 0.0 | 0 | 0.0 | 0 | 0.0 | 3 | 7.0 |
| Elevated platelet count | 1 | 4.3 | 0 | 0.0 | 0 | 0.0 | 0 | 0.0 | 1 | 4.3 | 0 | 0.0 | 2.0 | 10.0 | 0 | 0.0 | 0 | 0.0 | 2 | 10.0 | 1 | 2.3 | 2 | 4.7 | 0 | 0.0 | 0 | 0.0 | 3 | 7.0 |
| Abdominal discomfort | 2 | 8.7 | 0 | 0.0 | 0 | 0.0 | 0 | 0.0 | 2 | 8.7 | 1 | 5.0 | 0.0 | 0.0 | 0 | 0.0 | 0 | 0.0 | 1 | 5.0 | 3 | 7.0 | 0 | 0.0 | 0 | 0.0 | 0 | 0.0 | 3 | 7.0 |
| Diarrhea | 2 | 8.7 | 0 | 0.0 | 0 | 0.0 | 0 | 0.0 | 2 | 8.7 | 1 | 5.0 | 0.0 | 0.0 | 0 | 0.0 | 0 | 0.0 | 1 | 5.0 | 3 | 7.0 | 0 | 0.0 | 0 | 0.0 | 0 | 0.0 | 3 | 7.0 |
| Headache | 1 | 4.3 | 0 | 0.0 | 0 | 0.0 | 0 | 0.0 | 1 | 4.3 | 2 | 10.0 | 0 | 0.0 | 0 | 0.0 | 0 | 0.0 | 2 | 10.0 | 3 | 7.0 | 0 | 0.0 | 0 | 0.0 | 0 | 0.0 | 3 | 7.0 |
| Vertigo | 1 | 4.3 | 0 | 0.0 | 0 | 0.0 | 0 | 0.0 | 1 | 4.3 | 2 | 10.0 | 0 | 0.0 | 0 | 0.0 | 0 | 0.0 | 2 | 10.0 | 3 | 7.0 | 0 | 0.0 | 0 | 0.0 | 0 | 0.0 | 3 | 7.0 |
| Abnormal hepatic function | 2 | 8.7 | 0 | 0.0 | 0 | 0.0 | 0 | 0.0 | 2 | 8.7 | 1 | 5.0 | 0 | 0.0 | 0 | 0.0 | 0 | 0.0 | 1 | 5.0 | 3 | 7.0 | 0 | 0.0 | 0 | 0.0 | 0 | 0.0 | 3 | 7.0 |

**Table 11. Pharmacokinetic parameters on Day 1 of administration**

| Analyte | Dosing regimen | C_max_(ng/mL) | T_max_(h) | AUC_0-24_(h*ng/mL) | t_1/2_ (h) |
| --- | --- | --- | --- | --- | --- |
| ZG0163 | 100 mg b.i.d. (N = 8) | 333 (47) | 1.00 (1.00-3.00) | 2820 (33) | 3.38 (34) |
|  | 200 mg q.d. (N = 11) | 412 (31) | 2.00 (1.00-6.00) | 2260 (39) | 3.33 (36) |
| ZG0244 | 100 mg b.i.d. (N = 8) | 213 (32) | 3.00 (1.00-4.00) | 2980 (42) | 3.01 (29) |
|  | 200 mg q.d. (N = 11) | 434 (28) | 3.05 (1.97-8.00) | 3570 (32) | 3.88 (29) |
| ZG0245 | 100 mg b.i.d. (N = 8) | 31.4 (30) | 3.52 (1.00-11.9) | 468 (32) | 3.90 |
|  | 200 mg q.d. (N = 11) | 36.4 (49) | 4.00 (2.07-8.00) | 386 (34) | 6.76(24) |

Note: ZG0163 is the parent drug; ZG0244 and ZG0245 are the metabolites.

All enrolled subjects will be treated for at least 4 cycles, with 6 weeks per cycle or 24 weeks in total. Subjects who do not meet the discontinuation criteria at Week 24 may continue treatment with the investigational drug until the discontinuation criteria specified by the protocol are met. A safety evaluation will be carried out every 1-2 weeks in Cycle 1, every 3 weeks in Cycles 2-4, every 6 weeks after W24, and every 12 weeks after W48.

In this study, 12 subjects/group (12 pairs, 24 subjects in total) will be selected for PK analysis. Subjects participating in the PK analysis are required to be hospitalized during the PK blood sampling period, from 1 day before dose administration (D-1) to D7 of dose administration. Subjects will be discharged on D7.

In this study, 7 subjects/group (7 pairs, 14 subjects in total) will be selected for phosphorylated STAT3 (pSTAT3) analysis as a preliminary pharmacodynamics study. Subjects participating in the pSTAT3 analysis are required to be hospitalized during the blood sampling period, from 1 day before Week 24 or 30 (D167 or D209 after randomization) of treatment to D169 or D211 after randomization.

A steering committee may be appointed if necessary, which consists of at least the PI, physicians and statisticians with relevant clinical expertise. The steering committee will evaluate and provide the following recommendations: Modify the dose and dosing frequency based on safety considerations, or terminate the phase II trial.

A tumor assessment as well as a peripheral blood smear or bone marrow smear (only if the peripheral blood blasts are ≥ 20%) will be performed once every 12 weeks for subjects who discontinue the study for reasons other than PD, until PD, withdrawal of consent, or start of new anti-MF treatment.

During the trial, the investigator may perform unplanned MRI/CT test if a subject is suspected of PD.

## Dose Setting Basis

Safety data from a phase I dose escalation trial in healthy subjects showed that Jaktinib hydrochloride is well-tolerated. The multiple ascending dose study consisted of five dose groups: 100 mg q.d., 150 mg q.d., 100 mg b.i.d, 200 mg q.d., and 150 mg b.i.d. CTCAE Grade 3 neutrophil count decreased occurred in 5 healthy subjects in the highest dose group, i.e. 150 mg b.i.d. Although the MTD specified in the protocol was not reached, Jaktinib was not well-tolerated at 150 mg b.i.d. The pharmacokinetic analysis showed that at 100 mg and 200 mg, the exposure of Jaktinib was comparable to that of momelotinib but the half-life of Jaktinib was significant prolonged compared with momelotinib. Therefore, dose expansion at 100 mg b.i.d. and 200 mg q.d. was recommended.

In addition, the starting dose of momelotinib was 200 mg b.i.d., and 6 and 7 subjects were enrolled into the 200 mg b.i.d. and 250 mg b.i.d. group, respectively. Although no protocol-defined DLT was observed, treatment interruption occurred in 6 out of 7 subjects in the 250 mg b.i.d. group, resulting in dose reduction in 5 subjects and treatment discontinuation in 1 subject. Therefore, the Safety Review Committee decided to take 200 mg b.i.d. as the dosage for the expansion trial.

Based on the interim analysis of ZGJAK002, Jaktinib 100 mg b.i.d. was slightly superior to 200 mg q.d. in spleen response, symptom improvement, and anemia improvement. There was also no significant difference in tolerability between the 100 mg b.i.d. regimen and the 200 mg q.d. regimen. Moreover, daily exposure (AUC_0-24_) of the parent drug and metabolites of Jaktinib were comparable between the 200 mg q.d. and 100 mg b.i.d. regimens after the first dose. Exposure of the metabolite ZG0244 was comparable to that of the parent drug, while exposure of the metabolite ZG0245 was low. In summary, after a comprehensive assessment of risks and benefits, the additional 36 subjects will all be enrolled into the 100 mg b.i.d. group.

Dose expansions in the 100 mg b.i.d. and 200 mg q.d. groups will be carried out in this trial to preliminarily explore the safety and efficacy of Jaktinib hydrochloride tablets in the treatment of patients with myelofibrosis. Based on the human PK study, the half-life of Jaktinib is 5-8 hours. Serum drug concentrations were only slightly higher at 100 mg b.i.d. when compared with 100 mg q.d., and a safe and effective serum drug concentration was maintained. Therefore, 100 mg b.i.d. is a reasonable dose for the dose expansion study. To ensure subject safety, a detailed dose modification plan has been designed for this study. The dose of Jaktinib hydrochloride tablets will be modified throughout the course of treatment based on side effects.

## Randomization and Blinding Method

Approximately 100 subjects enrolled in Stage 1 of this study will be randomized in a 1:1 ratio into a Jaktinib hydrochloride group using the IWRS system. When the investigator logs into the central randomization system, the system will generate the randomization information for the subject after verifying subject eligibility. The investigator will provide the drug accordingly and record the information. For the specific procedure of randomization by IWRS, refer to the IWRS procedure section in the EDC guidelines. In Stage 2 of the study, an additional 36 subjects will all be added to the 100 mg b.i.d. group.

The investigator should be careful with the randomization and enrollment process, ensuring that the treatment start date (date of drug administration) is no more than 3 days apart from the date of randomization and enrollment. The system will submit the randomization and enrollment information of all subjects at the end of the study.

The study is an open-label trial rather than a blind trial.

# TRIAL POPULATION

Patients diagnosed with PMF according to WHO criteria (2016 Edition), or patients diagnosed with Post-PV-MF or Post-ET-MF according to IWG-MRT diagnostic criteria.

## Inclusion Criteria

1. Age ≥ 18 years, either male or female;
2. Patients diagnosed with PMF according to WHO criteria (2016 Edition), or patients diagnosed with Post-PV-MF or Post-ET-MF according IWG-MRT diagnostic criteria, with or without JAK2 mutations;
3. Patients with intermediate-2 or high risk myelofibrosis according to the DIPSS-plus scoring system; (Note: Patients with intermediate-1, intermediate-2 or high risk myelofibrosis are included in Study Protocol V1.0-6.0; Patients with intermediate-2/high risk myelofibrosis are included in Study Protocol V7.0.)
4. Subjects with no recent plans for stem cell transplantation;
5. Expected survival period is greater than 24 weeks;
6. ECOG PS 0-2;
7. Splenomegaly: greater than or equal to at least 5 cm below left costal margin by palpation;
8. Peripheral blood blasts ≤ 10%;
9. Patients who have not received prior treatment with JAK inhibitors; (Note: Patient who received treatment with JAK inhibitors for no more than 10 days may be enrolled)
10. Platelet count ≥ 75 × 10^9^/L and ANC ≥ 1000/μL without the aid of colony-stimulating factors, growth factors, thrombopoietin, or platelet transfusion. Subjects who have not received growth factors, colony-stimulating factors, thrombopoietin, or platelet infusion within 2 weeks prior to screening examinations;
11. Normal organ function within 7 days prior to randomization and enrollment: ALT and AST ≤ 2.5×ULN; DBIL and TBIL ≤ 2.0×ULN; Serum creatinine ≤ 1.5×ULN; CrCl ≥ 50 mL/min;
12. Subjects who meet the requirements of ethics committee, and voluntarily sign the informed consent form;
13. Ability to comply with the trial and follow-up procedures.

## Exclusion Criteria

1. Any significant clinical and laboratory abnormality which, in the opinion of the investigator, may affect the safety evaluation: a. Uncontrolled diabetes (> 250 mg/dL or > 13.9 mmol/L), b. Hypertension that cannot return to the following range (systolic blood pressure < 160 mmHg, diastolic blood pressure < 100 mmHg) despite treatment with one or two antihypertensive drugs, c. Peripheral neuropathy (NCI-CTC AE v4.03 Grade ≥ 2);
2. Patients with a history of congestive heart failure, uncontrolled or unstable angina or myocardial infarction, cerebrovascular accident, or pulmonary embolism within 6 months prior to screening;
3. Patients who have not fully recovered from surgical operation within 4 weeks prior to screening;
4. Patients with arrhythmia requiring treatment, or QTcB > 480 ms during screening;
5. Subjects with clinical symptoms of bacterial, viral, parasitic or fungal infections requiring treatment during screening;
6. Patients with a history of congenital or acquired bleeding disorders;
7. Patients who had undergone splenectomy or received radiotherapy to the spleen within 12 months before screening;
8. Patients with Anti-HIV positive, HBV positive (HBsAg positive, HBV-DNA positive or ≥ 1,000 copies/mL), anti-HCV positive or HCV-RNA positive results during screening;
9. Patients with epilepsy or treated with psychotropic drugs or sedatives (Note: except for estazolam tablets) at the time of screening;
10. Female patients who are planning pregnancy, already pregnant or breastfeeding, as well as patients who are unable to adopt effective contraceptive measures during the entire course of the trial; Male patients who do not use condoms during treatment and for 2 days (around 5 half-lives) after the last dose;
11. Patients who had malignant tumors within the past 5 years (except for basal cell carcinomas and cervical carcinoma in situ that have been cured);
12. Subjects with other serious concurrent diseases which, in the opinion of the investigator, may affect patient safety or compliance;
13. Subjects with suspected allergies to Jaktinib or other similar drugs;
14. Patients who have participated in another clinical trial involving a new drug or medical device and used the investigator drug or medical device within 3 months prior screening;
15. Patients who have received any medical treatment for MF (such as hydroxyurea), any immunomodulator (such as thalidoamide), any immunosuppressants, prednisone ≥ 10 mg/day or equivalent glucocorticoids, or growth factors (such as EPO) within 2 weeks prior to the randomization and enrollment, or within 6 half-lives of the drug.

## Treatment Discontinuation Criteria and Trial Termination Criteria

### 4.3.1 Individual subjects discontinued

**Subject dropouts**

The subject may choose to withdraw from the trial at any time without affecting further treatment. The subject shall be deemed as dropout cases in the following conditions during the trial, and the reason and date of dropout shall be recorded in the electronic Case Report Form (eCRF).

- The subject shall be deemed as a dropout case if any of the following occurs while there is no clear evidence of progressive disease:
- The subject refuses to continue participation in the trial;
- The subject decides to withdraw from the trial due to adverse events and there is no clear evidence of progressive disease;
- The subject is lost of follow up due to any reason;
- The subject fails to complete the last visit for other reasons.

### 4.3.2 Treatment discontinuationcriteria

It may be necessary for the investigator to discontinue treatment in a subject (without affecting further treatment) when any of the following occurs:

1. Grade 4 clinical adverse events (not laboratory measurements) that threaten the safety of the patient or prevent the subject from continuing the clinical trial, excluding fatigue, insomnia, obesity, and systemic symptoms (loss of function but not life threatening);
2. Grade 4 treatment-related laboratory abnormalities, considered clinically significant by the investigator, that threaten the safety of the patient or prevent the subject from continuing the clinical trial;
3. The patient cannot tolerate the lowest permitted dose (100 mg q.d.) in terms of hematological safety: a) Platelets cannot be maintained at ≥ 50 × 10^9^ /L; b) Absolute neutrophil count cannot be maintained at ≥ 500/μL; or hemoglobin cannot be maintained at ≥ 6.5 g/dL despite blood transfusion;
4. The subject becomes pregnant;
5. Evaluated as disease progression (defined as: a. ≥ 25% increase in spleen volume from nadir (including baseline); or b. bone marrow blasts ≥ 20%, as evidenced by the smear test; or c. peripheral blood blasts ≥ 20% as evidenced by the smear test and absolute blast count ≥ 1 × 10^9^/L lasting for at least 2 weeks, and in the opinion of the investigator, the subject is no longer suitable for continuing to participate in the clinical trial;
6. Other reasons that warrant treatment discontinuation according to the investigator;
7. Treatment should be discontinued if a new anti-MF treatment is initiated. Anti-MF treatments include: a. chemotherapy: such as cladribine, melphalan, busulfan, and hydroxyurea; b. radiotherapy to the spleen; c. splenectomy; d. allogeneic hematopoietic stem cell transplantation; e. other JAK inhibitors.

Appropriate examinations and observations should be performed upon treatment discontinuation. Also, the discontinuation date, cause, as well as examination and observation results should be documented in the eCRF. If necessary, the subject can be appropriately treated and followed up until the symptoms (examination values) return to the state before the trial or to the extent that no longer requires treatment, and the results shall be recorded in the eCRF. Subjects who discontinue the investigational drug must be followed up for disease progression. Subject who discontinue the investigational drug will not be replaced. Once the subject withdraws from the trial, the subject is not allowed to participate in the trial again.

A tumor assessment as well as a peripheral blood smear or bone marrow smear (only if the peripheral blood blasts are ≥ 20%) will be performed once every 12 weeks for subjects who discontinue the investigational drug for reasons other than PD, until PD, withdrawal of consent, or start of new anti-MF treatment. The investigator may perform unscheduled MRI/CT as well as peripheral blood smear or bone marrow smear (only if the peripheral blood blasts are ≥ 20%) at any time during the study if a subject is suspected of progression.

### 4.3.3 Trial termination

**Trial Termination Required by the Investigator**

The investigator may not be able to continue the trial for some reasons. According to the Good Clinical Practice (GCP), the investigator must inform the subjects, sponsor, Ethics Committee and CFDA of the trial termination and explain the reasons.

**Trial Termination Required by the Sponsor**

The sponsor may require all study sites or certain study site(s) to terminate the trial due to following reasons (including but not limited to):

1. Budget constraints;
2. Management reasons;
3. The enrolled number meets the requirement;
4. The investigator failed to follow the protocol, GCP, etc.;
5. The investigator failed to enroll sufficient subjects;
6. Safety concerns.

According to the GCP, the sponsor must inform the investigator(s), Ethics Committee and CFDA in writing before termination of the trial and explain the reasons.

**Trial Termination Required by the Regulatory Agency**

According to Drug Registration Regulations, CFDA may require the sponsor to revise the protocol, pause or terminate the trial due to one of the following circumstances during the trial:

1. The Ethics Committee failed to fulfill its responsibilities;
2. The safety of subjects cannot be ensured;
3. Failed to report serious adverse events within the specified time frame;
4. The drugs used in the trial are proved invalid;
5. Any quality issue of the investigational drug is found;
6. Falsification of data;
7. Other violations of GCP.

# INVESTIGATIONAL DRUG

## About the Investigational Drug

| Name: | Jaktinib hydrochloride tablets |
| --- | --- |
| Specification: | 50 mg/tablet |
| Dosage Form: | Tablet |
| Lot number / Expiration date: | TBD |
| Route of Administration: | per os |
| Storage Conditions: | Sealed, under 25 ℃, away from light |
| Supplier: | Suzhou Zelgen Biopharmaceuticals Co., Ltd. |

## Treatment Protocol

- The medication schemes of each dose group are shown as follows:

| **Dose group** | **Dosing regimen** | **Medication duration** |
| --- | --- | --- |
| Jaktinib hydrochloride tablets, 100 mg b.i.d. | Orally twice per day, 100 mg per dose, once every 12 hours, on an empty stomach | Subjects will continue treatment until any of the discontinuation criteria specified by the protocol is met. |
| Jaktinib hydrochloride tablets, 200 mg q.d. | Orally once per day, 200 mg per dose, once daily, on an empty stomach |  |
| * Fasting conditions: 0.5-1 hour before the meal or 2 hours after the meal. **Do not take the drug after a meal**. | | |

- For subjects of the PK study, the length of hospital stay shall be one day prior to drug administration (i.e., D-1) to D7. Subjects will be discharged on D7.
- Dose modification plan: The dose of Jaktinib hydrochloride tablets may be adjusted during treatment according to platelet count and neutrophil count (including dose reduction or interruption due to adverse reactions; as well as dose resumption or dose increase upon resolution of the adverse reaction). Refer to the table below for details:

| Platelet count | ▼Jaktinib hydrochloride tablets groups▼ | |
| --- | --- | --- |
|  | 100 mg (2 tablets) b.i.d. | 200 mg (4 tablets) q.d. |
|  | ▼Dose adjustments for Jaktinib hydrochloride tablets▼ | |
| ≥ 100 × 10^9^/L | Original dose or increased dose | Original dose or increased dose |
| 75 - <100×10^9^/L | 150 mg (3 tablets) q.d. | 150 mg (3 tablets) q.d. |
| 50 - < 75 × 10^9^/L | 100 mg (2 tablets) q.d. | 100 mg (2 tablets) q.d. |
| < 50 × 10^9^/L | Temporary discontinuation | Temporary discontinuation |
| Absolute neutrophil count | ▼Jaktinib hydrochloride tablets groups▼ | |
|  | 100 mg (2 tablets) b.i.d. | 200 mg (4 tablets) q.d. |
|  | ▼Dose adjustments for Jaktinib hydrochloride tablets▼ | |
| ≥ 1.5 × 10^9^/L | Original dose | Original dose |
| 1.0- <1.5×10^9^/L | 150 mg (3 tablets) q.d. | 150 mg (3 tablets) q.d. |
| 0.5- <1.0×10^9^/L | 100 mg (2 tablets) q.d. | 100 mg (2 tablets) q.d. |
| <0.5×10^9^/L | Temporary discontinuation | Temporary discontinuation |
| - The investigator may increase the dose whenever clinically indicated if PLT is persistently ≥ 100 × 10^9^/L and the drug is well-tolerated by the subject. Each dose increase should generally no exceed 50 mg/d and the maximum dose should not exceed 250 mg/d. Routine blood tests should be performed during unscheduled visits 2 and 4 weeks after the dose increase. Unscheduled visits may be skipped if they fall within the time window of other study visits. - During the study, treatment may be interrupted up to 3 weeks per cycle (6 weeks) due to hematological toxicities. A subject should withdraw from the study if the cumulative duration of treatment interruption exceeds > 3 weeks. | | |

In terms of non-hematologic safety, if Grade 3 or above non-hematologic toxicity associated with the investigational product occurs, drug administration must be discontinued. Before resuming the previous dose, any non-hematologic toxicities associated with the investigational product must reduce to ≤ Grade 1 (without time limit of discontinuation). The investigators are encouraged to consult the sponsor about resuming or increasing the dose after non-hematologic toxicities recover.

## Drug Packaging and Labeling

The label contains product name, serial number, clinical study approval number, specification, usage and dosage, batch number, expiration date, storage, supplier, precautions, etc., and should be noted with "Keep out of reach of children" and "For clinical research only".

## Storage, Management, and Dispensing of Drugs

For strict management and use of the investigational drug, all study sites shall establish strict drug management systems. Specific management staff should be designated for storage and dispensing of the investigational drug, which shall be well recorded.

Each study site must sign the drug receipt form upon receipt of the investigational drugs, and the drugs shall be kept in a locked specific cabinet. Study sites are required to designate staff for the management and distribution of the drugs, and the dispensing and retrieval shall be recorded in the specific record sheet in a timely manner.

The drug management personnel at the study sites will distribute the drug to the study nurse based on the randomization result and the corresponding medication number for each subject. The study nurse will then dispense the investigational drug to each subject. The dispensing process shall also be recorded accordingly.

## Medication Record and Return and Treatment Compliance

In order to evaluate the drug usage of subjects, the investigators must check the usage during each follow-up, and the unused drugs must be retrieved and checked. Each missing dose and the reasons must be well recorded.

Refer to the Statistical Analysis Plan (SAP) for treatment compliance calculation.

## Responsibility on Drug Management

The investigators shall receive, store, dispense and retrieve the drugs in accordance with relevant laws and regulations, and make corresponding records. The investigators must return unused drugs to the sponsor. The study sites are responsible for keeping the list of drug and cooperating with inspectors, auditors and relevant authorities for supervision, audit and inspection of the drug.

## Provisions of Concomitant Medications

Unless there is definite medical necessity, the subjects are prohibited from using drugs not mentioned in the trial protocol by principle.

If the investigators consider it necessary to use drugs not mentioned in the protocol, records in detail should be made in the concomitant medication/therapy sheet of eCRF, with the reasons, the method and the start and end time for the medication/therapy also documented.

### 5.7.1 Restricted/permitted concomitant therapy

1. Unconventional medicines and vitamin/mineral supplements, provided that the investigators consider they do not interfere with the endpoints of the study;
2. Supportive treatment: nonsteroid anti-inflammatory drugs for pain treatment; nutritional therapy and supportive care; blood transfusion when necessary; granulocyte colony stimulating factors and other hematopoietic growth factors for the treatment of acute, severe neutropenia when necessary;
3. Low-dose Warfarin (1 mg/day) for INR ≤ 2.0. During the trial, some subjects receiving Warfarin may experience rare bleeding or INR increase, so PT, INR or bleeding events shall be monitored regularly;
4. Prophylactic dose of Heparin (≤ 85 IU/kg);
5. Patients complicated by moderate to severe portal hypertension may receive local treatment before being enrolled in the study;
6. Patients complicated by pulmonary arterial hypertension can receive corresponding symptomatic treatment;
7. Antiplatelet therapy (small doses of Aspirin ≤ 100 mg/day);
8. Corticosteroids (prednisone < 10 mg/day or equivalent glucocorticoids);
9. Strong CYP3A4 inducers and inhibitors are not recommended. CYP2B6 substrates should be used with caution when combined with Jaktinib hydrochloride tablets.

### 5.7.2 Prohibited concomitant medications

1. Other JAK inhibitors;
2. Drugs or therapies for anemia correction and MF treatment: androgen, recombinant human erythropoietin, thalidomide, lenalidomide and prednisone ≥ 10 mg/day or equivalent glucocorticoids;
3. Any cytotoxic drugs and biological products for the treatment of myeloproliferative neoplasms, such as cladribine, melphalan, busulfan, fludarabine, cyclophosphamide, interferon, etc.;
4. Any other drugs used to treat splenomegaly, including Chinese traditional medicines and Chinese proprietary medicines;
5. Radiotherapy to the spleen;
6. Splenectomy;
7. Anti-epilepsy drugs or psychotropic drugs and sedatives. (Note: except for estazolam tablets)

The above prohibited drugs and therapies are not permitted during the entire clinical trial in principle, but in emergency situations, the investigators may use any therapies including drugs and non-drug therapies to ensure the safety of subjects.

## Compliance

The dosage and administration of Jaktinib hydrochloride tablets for each subject will be recorded in the eCRF in each course of treatment. The reasons for interruption and reduced or missing doses must be recorded in the eCRF.

The adherence will be evaluated with this information and the counting information of Jaktinib hydrochloride tablets in each course of treatment.

The compliance must be closely monitored, and any deviations must be reported to the sponsor.

The subjects' compliance with the treatment and trial protocol include the willingness to comply with all requirements in the trial protocol and to accept blood sampling for all safety assessments. According to the decision of the principal investigator or sponsor, the subjects may be removed from the study due to poor compliance with follow-up visits or investigational product.

# PROCEDURES

## Description

**(1) Informed consent**

The investigators shall explain all trial procedures to the subjects before screening, and shall obtain the Informed Consent Forms voluntarily signed by the subjects themselves.

**(2) Demographics**

Demographics of the subjects, including birth date, sex, ethnicity and other information, should be collected during the screening period. Childbearing potential of females should also be recorded. For female subjects with childbearing potential, recognized contraceptive measures should be taken throughout the trial. Pregnant and lactating women are not allowed to participate in this study.

**(3) Blood transfusion history, alcohol history, smoking history, allergies, medical history and concurrent diseases**

Complete medical history, surgical history, drug allergies, as well as alcohol or drug dependence history within 1 year before the start of the trial should be collected during screening. The investigators shall verify the inclusion/exclusion eligibilities of the subjects based on their medical history.

**Focus on complete medical history related to myelofibrosis**, including constitutional symptoms (weight loss, night sweats, and fever), history of "white blood cell > 25 × 10^9^/L", detailed history of "HGB < 100g/L" (whether there was a history of RBC transfusion, e.g., blood transfusion required for MF-related anemia; whether there was at least two consecutive tests of HGB < 100 g/L, that is, MF-related anemia), detailed history of "platelet < 100 × 10^9^/L” (whether there was a history of platelet transfusion, e.g., blood transfusion required for MF-related platelet decrease; whether there was at least two consecutive tests of PLT < 100 × 10^9^/L, i.e. MF-related platelet decrease), history of “peripheral blood blasts ≥ 1%”, history of “unfavorable karyotype”, and treatment history including medications, surgery, and blood transfusion history (with a complete description of blood transfusion time, type of blood transfusion, cause of blood transfusion, and unit amount (U), especially the time and unit amount (U) of RBC transfusion within 30 days prior to administration of the investigational drug).

**(4) Medication history and concomitant medication**

Drugs used by the subjects within 1 month before the start of the trial during the screening period, including prescription drugs, over-the-counter drugs, Chinese herbal medicines and other investigational products. The investigators shall verify the inclusion/exclusion criteria of the subjects based on their medical history.

**(5) Vital signs and physical examinations**

Perform vital signs and physical examinations during screening (D-28 to D-1), visit 0 (D1), treatment (Week 1, 2, 4, 6, 9, 12, 15, 18, 21, and 24, every 6 weeks during Weeks 24-48, and every 12 weeks after Week 48), and follow-up (EOT visit and safety follow-up).

Vital signs include blood pressure, respiration, body temperature, and pulse. Blood pressure, pulse, and respiration shall be measured at least 5 minutes after the subjects sit quietly.

Perform a complete physical examination of each system during screening, the treatment period, and the follow-up period. Document the examination results, including height, weight, and each organ system (including measurement of palpable spleen). Height measurements are not required during the treatment period and the follow-up period. Document the measurement of palpable spleen in cm, and measure the distance from the costal margin to the farthest splenic edge by palpation and a ruler.

**(6) Laboratory tests**

Perform routine blood tests during screening (D-28 to D-1), visit 0 (D1; not required if results within 7 days prior to randomization and enrollment are available), treatment (Week 1, 2, 4, 6, 9, 12, 15, 18, 21, and 24, every 6 weeks during Weeks 24-48, and every 12 weeks after Week 48), and follow-up (EOT visit and safety follow-up).

Perform blood biochemistry (including hepatic and renal function), serum EPO (except for visit 0, carried out by the central laboratory), coagulation function, and routine urinalysis during screening (D-28 to D-1), visit 0 (D1; not required if results within 7 days prior to randomization are available), treatment (Week 6, 12, 18, and 24, every 6 weeks during Weeks 24-48, and every 12 weeks after Week 48), and follow-up (EOT visit and safety follow-up). Perform serologic tests during screening only.

Routine blood test includes: red blood cell (RBC), hemoglobin (HGB), platelet (PLT) and white blood cell (WBC) and their percentages, differential counts and percentages of WBC (basophil BASO#, eosinophil EO#, lymphocyte LYMPH#, monocyte MONO#, and neutrophil NEUT#). The collection methods of blood samples should be consistent for all blood routine.

Blood biochemistry (including hepatic and renal function): aspartate aminotransferase (AST), alanine aminotransferase (ALT), alkaline phosphatase (ALP), gamma-glutamyl transpeptidase (γ-GT), lactate dehydrogenase (LDH), total bilirubin (TBIL), direct bilirubin (DBIL), total protein (TP), albumin (ALB), urea/blood urea nitrogen (BUN), creatinine (Cr), phosphorus (P), sodium (Na^+^), potassium (K^+^), chloride (Cl^-^), calcium (Ca^2+^), magnesium (Mg^2+^), and glucose (GLU). The collection methods of blood samples should be consistent for all blood biochemistry tests and performed under fasting conditions.

Coagulation test: prothrombin time (PT), thrombin time (TT), activated partial thromboplastin time (APTT), and international normalized ratio (INR). The collection methods of blood samples should be consistent for all coagulation function tests.

Routine urinalysis includes: pH, protein (U-PRO), glucose (U-GLU), white blood cell (U-WBC), urinary red blood cell (U-RBC), etc.

**(7) Bone marrow examination**

Peripheral blood smear: including cell morphology, counts and percentages. Perform the examination during screening (D-28 to D-1), treatment (Week 6, 12, 18, and 24, every 6 weeks during Weeks 24-48, and every 12 weeks after Week 48), and the EOT visit.

Bone marrow smear: This examination shall be carried out during the screening period, and carried out when the peripheral blood smear shows a "-blast" cells percentage ≥ 20%.

Bone marrow aspiration and biopsy: Perform bone marrow aspiration and pathological biopsy during screening, including cytological analysis, reticular fiber (silver) staining, chromosome karyotyping (peripheral blood specimens may be used in case of ‘dry tap’; may be tested at the central laboratory if unavailable at the study site) and pathological biopsy to confirm MF diagnosis. The investigator may also perform a bone marrow biopsy during treatment and at the EOT visit if clinically indicated. In addition, perform a peripheral blood smear or bone marrow smear (only if the peripheral blood blasts are ≥ 20%) once every 12 weeks during follow-up for subjects who discontinue the study for reasons other than PD, until PD, withdrawal of consent, or start of new anti-MF treatment.

**(8) Mutation tests**

Mutation test: Perform JAK2 V617F (quantitative and qualitative), CALR (qualitative), MPL W515L/K (qualitative), and BCR-ABL genetic tests during screening. Genetic testing is carried out by the central laboratory. Perform a JAK2 V617F mut test (quantitative and qualitative) at the EOT visit only in subjects who test positive for JAK2 V617F mut at baseline (qualitative).

(Results of bone marrow aspiration and biopsy, bone marrow smear, and mutation test obtained within 4 weeks prior to randomization and enrollment may be used as screening results with the investigator’s approval, under which circumstances reexaminations are not required during screening.)

**(9) 12-Lead electrocardiogram**

Perform 12-lead ECG during screening (D-28 to D-1), visit 0 (D1; not required if results within 7 days prior to randomization and enrollment are available), treatment period (Week 6, 12, 18, and 24, every 6 weeks during Weeks 24-48, and every 12 weeks after Week 48), and during follow-up (EOT visit and safety follow-up).

The subjects shall rest quietly for at least 5 min before the 12-lead ECG.

**(10) Color Doppler echocardiography**

Except during screening and safety follow-up, perform an echocardiography only when ECG results are abnormal and clinically significant, and only document left ventricular ejection fraction.

**(11) Blood pregnancy test**

Female subjects of childbearing age must undergo pregnancy test during the screening ((D-28 to D-1), visit 0 (D1; not required if results within 7 days prior to randomization and enrollment are available), and during safety follow-up. Subjects must test negative during screening to be eligible for enrollment.

**(12) ECOG PS scoring**

Perform ECOG PS scoring during screening (D-28 to D-1), visit 0 (D1; not required if results within 7 days prior to randomization and enrollment are available), treatment (Week 6, 12, 18, and 24, every 6 weeks during Weeks 24-48, and every 12 weeks after Week 48), and follow-up (EOT visit and safety follow-up).

ECOG PS scores are evaluated by the investigators.

**(13) MRI/CT**

Perform abdominal MRI/CT during screening. The result must be reviewed by the IRC. The spleen volume must be measurable so that the subject can be enrolled.

Perform MRI/CT during screening, treatment (Week 12 and 24, and every 12 weeks after Week 24), and at the EOT visit. Radiographic (MRI/CT) confirmation is required if spleen response is used to assess treatment efficacy. The investigator may perform unscheduled MRI/CT. In addition, perform once every 12 weeks during follow-up for subjects who discontinue the study for reasons other than PD, until PD, withdrawal of consent, or start of new anti-MF treatment. During the trial, the investigator may perform unplanned MRI/CT test if a subject is suspected of PD.

MRI/CT data of the spleen must be confirmed by the IRC. Independent readers shall depict the circumferences of spleen and liver, and the volumes of spleen and liver should be calculated using formula and volumetric images.

(Results of radiographic assessments obtained within 4 weeks prior to randomization may be used as screening results with the investigator’s approval and confirmation by the IRC, under which circumstances reexaminations are not required during screening)

(Refer to the SOP of the IRC for details)

**(14) Collection of adverse events**

The adverse events of subjects shall be closely monitored throughout the entire trial until the investigators confirm that the adverse events have recovered or have no clinical significance. AE collection period: from the signing of informed consent form to safety follow-up (D28 after the last dose of the investigational drug). See Section "9. Adverse Events and Serious Adverse Events" for details.

# TRIAL PROCEDURES

## Screening Period (D-28 to D-1)

- Signing of informed consent form;
- Collecting baseline demographics/past medication/medical history;
- Vital signs examination;
- Physical examination;
- MRI/CT scan: Results of radiographic assessments obtained within 4 weeks prior to randomization may be used as screening results with the investigator’s approval and confirmation by the IRC, under which circumstances reexaminations are not required during screening;
- ECOG PS scoring;
- MPN-SAF TSS scoring;
- Anemia response assessment;
- Peripheral blood smear;
- Bone marrow smear: Results of bone marrow smear obtained within 4 weeks prior to randomization and enrollment may be used as screening results with the investigator’s approval, under which circumstances reexaminations are not required during screening;
- Bone marrow aspiration and biopsy: Results of bone marrow aspiration and biopsy obtained within 4 weeks prior to randomization and enrollment may be used as screening results with the investigator’s approval, under which circumstances reexaminations are not required during screening;
- Mutation test: Results of mutation tests obtained within 4 weeks prior to randomization and enrollment may be used as screening results with the investigator’s approval, under which circumstances reexaminations are not required during screening;
- Laboratory tests: routine blood test, routine urinalysis, blood biochemistry (including liver and kidney function tests) and coagulation test;
- Serum EPO;
- Infection test;
- Blood pregnancy test;
- 12-lead ECG;
- Color Doppler echocardiography;
- Confirmation of inclusion/exclusion criteria;
- Recording of adverse events;
- Recording of concomitant medication and therapy;

## Baseline Period

Visit 0 (D1, day of randomization)

- Vital signs examination;
- Physical examination;
- Review of inclusion/exclusion criteria;
- ECOG PS score: Reexaminations before the first dose are not required if results within 7 days prior to randomization and enrollment are available.
- Laboratory tests: routine blood test, routine urinalysis, blood biochemistry (including hepatic and renal function), and coagulation function. Reexaminations before the first dose are not required if results within 7 days prior to randomization and enrollment are available.
- Blood pregnancy test: Reexaminations before the first dose are not required if results within 7 days prior to randomization and enrollment are available;
- 12-Lead ECG: Reexaminations before the first dose are not required if results within 7 days prior to randomization and enrollment are available;
- Randomization and enrollment;
- Dispensing of investigational drug;
- Dispensing of subject dairy card;
- Recording of concomitant therapy;
- Recording of adverse events;
- Collection of PK blood samples: for subjects participating in PK study only. See Section 8.3 for detailed requirements;
- Appointment for the next visit.

Note: If the 28th day after the signing of the informed consent is a holiday or weekend, a delay of up to 3 days is permitted for randomization and enrollment without being considered a protocol deviation (i.e. in this case, the subject must be randomized within 31 days after signing the ICF).

## Treatment Period

**Visit 1 (Week 1, Day 7 ± 1)**

- Vital signs examination;
- Physical examination;
- Laboratory tests: Routine blood test.
- Dispensing/retrieval of investigational drug;
- Dispensing/retrieval of subject dairy card;
- Recording of adverse events;
- Recording of concomitant therapy;
- PK blood sampling: For subjects participating in the PK study only;
- Appointment for the next visit.

**Visit 2 (Week 2, Day 14 ± 1)**

- Vital signs examination;
- Physical examination;
- Laboratory tests: Routine blood test.
- Dispensing/retrieval of investigational drug;
- Dispensing/retrieval of subject dairy card;
- Recording of adverse events;
- Recording of concomitant therapy;
- Appointment for the next visit.

**Visit 3 (Week 4, Day 28 ± 1)**

- Vital signs examination;
- Physical examination;
- Laboratory tests: Routine blood test.
- Dispensing/retrieval of investigational drug;
- Dispensing/retrieval of subject dairy card;
- Recording of adverse events;
- Recording of concomitant therapy;
- Appointment for the next visit.

**Visit 4 (Week 6, Day 42 ± 3)**

- Vital signs examination;
- Physical examination;
- Anemia response assessment;
- ECOG PS scoring;
- MPN-SAF TSS scoring;
- Peripheral blood smear;
- Bone marrow smear: Perform a bone marrow smear only if the peripheral blood blasts are ≥ 20%; perform bone marrow biopsy only if considered necessary by the investigator;
- Serum EPO test;
- Laboratory tests: routine blood test, routine urinalysis, blood biochemistry (including liver and kidney function tests) and coagulation test;
- 12-lead ECG;
- Color Doppler echocardiography: Perform only when 12-lead ECG results are abnormal and clinically significant, and only document left ventricular ejection fraction;
- Dispensing/retrieval of investigational drug;
- Dispensing/retrieval of subject dairy card;
- Recording of adverse events;
- Recording of concomitant therapy;
- Appointment for the next visit.

**Visit 5 (Week 9, Day 63 ± 3)**

- Vital signs examination;
- Physical examination;
- Laboratory tests: Routine blood test.
- Dispensing/retrieval of investigational drug;
- Dispensing/retrieval of subject dairy card;
- Recording of adverse events;
- Recording of concomitant therapy;
- Appointment for the next visit.

**Visit 6 (Week 12, Day 84 ± 3)**

- Vital signs examination;
- Physical examination;
- MRI/CT scan (spleen response assessment);
- Anemia response assessment;
- ECOG PS scoring;
- MPN-SAF TSS scoring;
- MF response assessment: See Appendix VI;
- Peripheral blood smear;
- Bone marrow smear: Perform a bone marrow smear only if the peripheral blood blasts are ≥ 20%; perform bone marrow biopsy only if considered necessary by the investigator;
- Serum EPO test;
- Laboratory tests: routine blood test, routine urinalysis, blood biochemistry (including liver and kidney function tests) and coagulation test;
- 12-lead ECG;
- Color Doppler echocardiography: Perform only when ECG results are abnormal and clinically significant, and only document left ventricular ejection fraction;
- Dispensing/retrieval of investigational drug;
- Dispensing/retrieval of subject dairy card;
- Recording of adverse events;
- Recording of concomitant therapy;
- Appointment for the next visit.

**Visit 7 (Week 15, Day 105 ± 3)**

- Vital signs examination;
- Physical examination;
- Laboratory tests: Routine blood test.
- Dispensing/retrieval of investigational drug;
- Dispensing/retrieval of subject dairy card;
- Recording of adverse events;
- Recording of concomitant therapy;
- Appointment for the next visit.

**Visit 8 (Week 18, Day 126 ± 3)**

- Vital signs;
- Physical examination;
- Anemia response assessment;
- ECOG PS scoring;
- MPN-SAF TSS scoring;
- Peripheral blood smear;
- Bone marrow smear: Perform a bone marrow smear only if the peripheral blood blasts are
  ≥ 20%; perform bone marrow biopsy only if considered necessary by the investigator;
- Serum EPO test;
- Laboratory tests: routine blood test, routine urinalysis, blood biochemistry (including liver and kidney function tests) and coagulation test;
- 12-lead ECG;
- Color Doppler echocardiography: Perform only when ECG results are abnormal and clinically significant, and only document left ventricular ejection fraction;
- Dispensing/retrieval of investigational drug;
- Dispensing/retrieval of subject dairy card;
- Recording of adverse events;
- Recording of concomitant therapy;
- Appointment for the next visit.

**Visit 9 (Week 21, Day 147 ± 3)**

- Vital signs examination;
- Physical examination;
- Laboratory tests: Routine blood test.
- Dispensing/retrieval of investigational drug;
- Dispensing/retrieval of subject dairy card;
- Recording of adverse events;
- Recording of concomitant therapy;
- Appointment for the next visit.

**Visit 10 (Week 24, Day 168 ± 3)**

- Vital signs examination;
- Physical examination;
- MRI/CT scan (spleen response assessment);
- Anemia response assessment;
- ECOG PS scoring;
- MPN-SAF TSS scoring;
- MF response assessment: See Appendix VI;
- Peripheral blood smear;
- Bone marrow smear: Perform a bone marrow smear only if the peripheral blood blasts are ≥ 20%; perform bone marrow biopsy only if considered necessary by the investigator;
- Serum EPO test;
- Laboratory tests: routine blood test, routine urinalysis, blood biochemistry (including liver and kidney function tests) and coagulation test;
- 12-lead ECG;
- Color Doppler echocardiography: Perform only when 12-lead ECG results are abnormal and clinically significant, and only document left ventricular ejection fraction;
- Dispensing/retrieval of investigational drug;
- Dispensing/retrieval of subject dairy card;
- Recording of adverse events;
- Recording of concomitant therapy;
- pSTAT3 blood sampling: For subjects participating in the PD study only (during Week 30 of the treatment period if not performed during this visit);
- Appointment for the next visit.

**Visits 11, 12, 13...**

Subjects who do not meet the discontinuation criteria specified by the protocol after 24 weeks may continue treatment with the investigational drug until any of the discontinuation criteria specified by the protocol is met. Perform follow-up once every 6 weeks during Weeks 24-48. Perform follow-up once every 12 weeks after Week 48. Perform the following assessments during each follow-up:

- Vital signs examination;
- Physical examination;
- Laboratory tests: routine blood test, routine urinalysis, blood biochemistry (including liver and kidney function tests) and coagulation test;
- Anemia response assessment;
- ECOG PS scoring;
- MRI/CT scan (Perform once every 12 weeks until PD, withdrawal of consent, or start of new anti-MF treatment. Tumor assessment is a must prior to starting a new anti-MF treatment).
- MPN-SAF TSS scoring;
- MF response assessment: See Appendix VI (once every 12 weeks);
- Peripheral blood smear/bone marrow smear (every 6 weeks during Weeks 24-48, and every 12 weeks after Week 48; perform a bone marrow smear only if the peripheral blood blasts are ≥ 20%); perform bone marrow biopsy only if considered necessary by the investigator;
- Serum EPO test;
- 12-lead ECG;
- Color Doppler echocardiography: Perform only when 12-lead ECG results are abnormal and clinically significant, and only document left ventricular ejection fraction;
- Dispensing/retrieval of investigational drug;
- Dispensing/retrieval of subject dairy card;
- Recording of adverse events;
- Recording of survival status;
- Recording of concomitant therapy;

## End-of-Treatment (EOT) Follow-Up

Complete the following assessments at the EOT visit (+ 7 days after the last dose of the investigational drug):

- Vital signs examination;
- Physical examination;
- CT/MRI scan;
- Peripheral blood smear;
- Bone marrow smear: Perform a bone marrow smear only if the peripheral blood blasts are ≥ 20%; perform bone marrow biopsy only if considered necessary by the investigator;
- Qualitative and quantitative test of JAK2 V617F mutation (for subjects with positive baseline results only);
- Serum EPO;
- ECOG PS scoring;
- MPN-SAF TSS scoring;
- MF response assessment: See Appendix VI;
- Anemia response assessment;
- Laboratory tests: routine blood test, routine urinalysis, blood biochemistry (including liver and kidney function tests) and coagulation test;
- 12-lead ECG;
- Color Doppler echocardiography: Perform only when 12-lead ECG results are abnormal and clinically significant, and only document left ventricular ejection fraction;
- Retrieval of investigational drug;
- Retrieval of subject dairy card;
- Recording of concomitant medication and therapy;
- Recording of adverse events.

Complete the following during the safety follow-up (28 ± 7 day after the last dose of the investigational drug):

- Vital signs examination;
- Physical examination;
- ECOG PS scoring;
- Laboratory tests: routine blood test, routine urinalysis, blood biochemistry (including liver and kidney function tests) and coagulation test;
- Blood pregnancy test;
- 12-lead ECG;
- Color Doppler echocardiography;
- Recording of concomitant medication and therapy;
- Recording of adverse events.

## Survival Follow-up

Within 6 months after the EOT visit, subjects should be followed up by telephone calls every 3 months (± 7 days). Six months after the EOT visit, the follow-up frequency will be once every 6 months (± 7 days). Each follow-up shall include:

- Recording of survival status;
- Recording of other concomitant anti-MF therapies (collecting names of drugs or surgeries or other treatments only).

# ASSESSMENTS

## Safety Evaluations

- Overall safety assessments: vital signs, physical examination, and ECOG PS score;
- Laboratory safety tests: include routine blood, routine urinalysis, blood biochemistry, liver and kidney function tests, and coagulation test for each safety follow-up;
- 12-Lead ECG, color Doppler electrocardiography (only when 12-lead ECG results are abnormal and clinically significant);
- The severity and incidence of adverse events and adverse reactions (NCI-CTCAE version 4.03);
- Incidence of thrombotic events.
- Arterial thrombosis: 1) coronary atherosclerosis heart disease; 2) cerebral artery thrombosis; 3) peripheral arterial occlusive disease: such as mesenteric arterial thrombosis and arterial thrombosis of extremities.
- Venous thrombosis: 1) thrombophlebitis; 2) deep vein thrombosis; 3) pulmonary embolism.
- Microcirculatory thrombosis: 1) thrombotic thrombocytopenic purpura; 2) hemolytic uremic syndrome; 3) thrombosis in extracorporeal circulation; 4) other conditions: such as purpura fulminans and disseminated intravascular coagulation.

## Efficacy Evaluation

### 8.2.1 Primary efficacy endpoint

- Response rate: the proportion of subjects with a ≥35% reduction in spleen volume at week 24;

### 8.2.2 Secondary Efficacy Endpoints

- Objective response rate (CR + PR): IWG-MRT response criteria
- Spleen Response:
- Best response rate: the proportion of subjects with at least one spleen volume reduction ≥ 35% against the baseline;
- Time to response: the time from the date of randomization and enrollment to the first date of ≥ 35% reduction in spleen volume from baseline;
- DoMSR: the time between the first occurrence of spleen volume reduction of ≥ 35% from baseline to an increase in spleen volume so that the reduction is less than 35% from baseline;
- Anemia Response:
- Proportion of transfusion-dependent patients at baseline turned into transfusion-independent patients (transfusion-independent patients: no transfusion for at least 12 consecutive weeks and HGB ≥ 85 g/L)
- Proportion of patients with a ≥ 20 g/L increase in HGB who are transfusion-independent (HGB ≤ 100 g/L) at baseline;
- Reduced RBC-transfusion-dependence: 50% decrease in the number of RBC transfusions.

Patients who are RBC-transfusion-dependent at baseline are defined as patients who received ≥ 2U of RBC transfusion within 30 days prior to treatment with the investigational drug

- Response rate of MF-related symptoms
- Proportion of patients with a ≥ 50% reduction in MPN-SAF TSS;
- The decrease in MPN-SAF TSS from baseline
- PFS: The time from the date of randomization and enrollment to the date of any of the following events: 1) ≥ 25% increase in spleen volume from nadir (including baseline); 2) death from any cause.
- LFS: The time from the date of randomization and enrollment to the date of any of the following events: 1) first bone marrow blasts ≥ 20%; 2) first peripheral blood blasts ≥ 20% and absolute blast count ≥ 1 × 109/L lasting for at least 2 weeks; 3) death from any cause.
- OS: the time from the date of randomization and enrollment to the date of death from any cause;

### 8.2.3 Scanning and calculation of liver and spleen volumes

Abdominal MRI/CT scan and 3D image of the spleen/liver:

1. Spleen volume (SV): Measure the largest width (W, cm), the largest thickness (T, cm) and the largest length (L, cm) of the spleen, and calculate the volume of the spleen using the following formula: SV = 30 + 0.58 × (L × W × T);
2. Liver volume (LV): Measure the largest cranio-caudal dimension (CC, cm), the largest latero-lateral dimension (LL, cm), and the largest antero-posterior dimension (AP, cm) of the liver, and calculate the liver volume using the following formula: LV = (CC × LL × AP) × 0.31.

All MRI/CT data of the spleen/liver must be confirmed by the IRC. Independent readers shall depict the three-dimensional circumferences of spleen and liver, and the volumes of spleen and liver should be calculated using formula and volumetric images as specified in the protocol.

(Refer to the SOP of the IRC for details)

## Pharmacokinetics Study

All subjects participating in the PK study must undergo blood sample collection according to trial requirements.

PK sampling points are as follows:

| **Study time (day)** | **PK blood sampling points** | | | | | | | |
| --- | --- | --- | --- | --- | --- | --- | --- | --- |
|  | **Before administration** | **After administration** | | | | | | |
|  |  | **1.0 h** | **2.0 h** | **3.0 h** | **4.0 h** | **6.0 h** | **8.0 h** | **12 h** |
| D1 | X | X | X | X | X | X | X | X |
| D2 | X |  |  | X |  |  |  |  |
| D3 | X |  |  | X |  |  |  |  |
| D4 | X |  |  | X |  |  |  |  |
| D5 | X |  |  | X |  |  |  |  |
| D6 | X |  |  | X |  |  |  |  |
| D7 | X |  |  |  |  |  |  |  |
| Blood sampling at the median cubital vein, 4 mL each time;  Before administration: within 10 min before administration;  1-4 hours after administration: within ± 5 minutes;  6-12 hours after administration: within ± 10 minutes;  12 hours after administration: Must be collected prior to the evening dose.  Subjects participating in the PK analysis are required to be hospitalized during the PK blood sampling period, from 1 day before dose administration (D-1) to D7 of dose administration. Subjects will be discharged on D7. | | | | | | | | |

During the trial, blood samples for PK analysis are collected at scheduled time points according to the protocol. Each blood sample is identified by protocol number, subject number, date, sampling time and sample matrix. All blood samples shall be pre-frozen within 50 min from the beginning of collection. The samples shall be delivered to the sponsor's contract bioanalysis laboratory to determine the plasma concentration of Jaktinib and its metabolites using a verified method. Procedures for collection, processing, storage and transportation of samples (if required) shall be provided in a separate laboratory manual (see the Operation Manual for details).

Main pharmacokinetic parameters include: AUC_last,_ AUC_inf_, C_max_, T_max_, CL/F, V_d_/F, and t_1/2_.

# ADVERSE EVENTS AND SERIOUS ADVERSE EVENTS

## Adverse Events

### 9.1.1 Definition of adverse event

Adverse event (AE): Untoward medical occurrence in a subject administered with a pharmaceutical product. The event does not necessarily have a causal relationship with the investigational product, and covers any new or worsening conditions in severity and frequency compared with the baseline, including abnormalities of laboratory tests, medical examinations, and other diagnostic methods.

### 9.1.2 Collection and recording of adverse events

The investigator should collect and document AEs in each subject from the signing of the informed consent form to the safety follow-up (D28 after the last dose of the investigational drug). All adverse events should be coded according to MedDRA terms. The investigator is responsible for observing, recording and following up any adverse event during the study regardless of the cause.

For adverse events, only worsening conditions with clinical significance against the baseline will be recorded. Abnormalities of screening or baseline examinations are recorded as medical history, rather than adverse events.

Any AEs during the trial, regardless of the severity or correlation with the investigational product, shall be recorded in the AE sheet of eCRF, documenting the occurrence date, symptoms, severity (as per Toxicity Grading Scale), duration, treatment and outcome, etc. The correlation with the investigational product and the relation with underlying diseases will be evaluated after taking into account complications and concomitant medications. The record shall be provided with a signature and a date. AEs shall be recorded using medical terms and provided with diagnosis of the disease as far as possible instead of listing symptoms and signs.

The investigators must determine whether the abnormal laboratory test results have clinical significance. In case of clinical significance, the abnormalities shall be recorded on the original medical record and eCRF, and determined whether they are AEs.

Unexplainable laboratory abnormalities must be re-examined and followed up until the measurements return to the normal range or baseline level and/or have reasonable and adequate interpretation, and shall be recorded on the original medical record and eCRF.

The investigator shall follow up the subjects with adverse events until the adverse events recover (return to normal state or to baseline), until stable disease or until there is reasonable interpretation.

### 9.1.3 Criteria for relationship between adverse events and the drug

According to *Adverse Drug Reactions Reporting and Monitoring Manual* and *Provisions for Adverse Drug Reactions Reporting and Monitoring, and Serious Adverse Event Report* , the investigator should perform a causality assessment based on the 5 principles, classifying each adverse reaction/event into one of the 5 categories, i.e. "definitely related, possibly related, unlikely related, not related, or indeterminable". Adverse reactions refer to events classified as "definitely related, possibly related, or indeterminable". The adverse drug reactions should be recorded using medical terms. AE without a causality assessment will be considered “possibly related” to the investigational drug.

• Definitely related: • Reasonable time relationship +

• Known adverse reaction type +

• Positive dechallenge +

• Positive rechallenge +

• Might be explained by other causes -

• Possibly related: • Reasonable time relationship +

• Known adverse reaction type ±?

• Positive dechallenge ±?

• Positive rechallenge?V

• Might be explained by other causes ±?

• Unlikely related: Reasonable time relationship -

• Known adverse reaction type -

• Positive dechallenge ±?

• Positive rechallenge?

• Might be explained by other causes ±?

• Not related: Reasonable time relationship -

• Known adverse reaction type -

• Positive dechallenge -

• Positive rechallenge -

• Might be explained by other causes +

• Indeterminable: • Data necessary for assessment cannot be obtained

**Causality Assessment Criteria**

|  | **Definitely related** | **Possibly related** | **Unlikely related** | **Not related** | **Indeterminable** |
| --- | --- | --- | --- | --- | --- |
| Reasonable time relationship | + | + | - | - | Data necessary for assessment cannot be obtained |
| Known adverse reaction type | + | ±? | - | - |  |
| Positive dechallenge | + | ±? | ±? | - |  |
| Positive rechallenge | + | ? | ? | - |  |
| Can be explained by other causes | - | ±? | ±? | + |  |
| Note: + Yes - No ± Maybe ? Unknown | | | | | |

### 9.1.4 Severity grading scale for adverse events

Grades or severities of adverse events by National Cancer Institute Common Terminology Criteria for Adverse Events, Version 4 (NCI-CTC AE V4.03) should be recorded.

## Serious Adverse Events

### 9.2.1 Definition of serious adverse event

Serious Adverse Event (SAE): Any of the following situations during the clinical trial shall be considered as serious adverse event:

1. Requiring hospitalization;
2. Prolonged hospital stay;
3. Disability;
4. Incapacity;
5. Life-threatening experience or death;
6. Congenital malformation;
7. Other important medical event that may not result in Articles 1 to 6 may be considered a serious adverse drug event when, based upon medical judgment, it may jeopardize the subject and may require medical or surgical intervention to prevent one of the outcomes listed in Articles 1 to 6.

The following are not considered SAE: 1) Progressive disease: progressive disease (including signs and symptoms of progression) should not be reported as a serious adverse event during the trial or safety reporting period; 2) Death, hospitalization/prolonged hospitalization and disability caused by the disease itself or direct complications should not be reported as a serious adverse event.

### 9.2.2 Reporting of serious adverse events

If an SAE occurs during the trial, the investigator must fill out the serious adverse report , and submit it to the pharmaceuticals supervisory and administrative department, the department of public health administration, the sponsor, and the ethics committee within 24 hours. The investigator must sign and date the report.

The sponsor, Suzhou Zelgen Biopharmaceuticals Co., Ltd., or its designated CRO must report a serious adverse event to drug regulatory authorities and the department of public health administration immediately upon learning of the serious adverse event, and notify other hospitals participating in the trial.

- Ethics Committee of The First Affiliated Hospital, Zhejiang University

Contact: Zhou Huili Tel: 0571-87236685 Fax: 0571-87236685

Address: No. 79, Qingchun Road, Hangzhou, Zhejiang Postal Code: 310058

- Suzhou Zelgen Biopharmaceuticals Co., Ltd.

Contact: Wu Liqing Tel: 0512-57309965 Mobile: 18015826715

Fax: 0512-57309965 E-mail: wulq@zelgen.com

Address: No. 209, Chenfeng Road, Kunshan, Jiangsu

### 9.2.3 Responsibilities of various parties for serious adverse events

**Responsibilities of the sponsor**

1. The sponsor should provide the investigator with legal and economic guarantees, except for medical accident;
2. To provide corresponding treatment and other expenses for serious adverse events which may be associated with the trial or investigational drug;
3. To provide corresponding financial compensation for serious adverse events which may be associated with the trial or investigational drug;
4. To quickly learn about the serious adverse events during the trial from the investigators;
5. To timely report to relevant units as required by GCP;
6. To provide research and development data of corresponding drugs as deemed necessary by the investigator;
7. To provide other assistance as required by the investigator.

**Responsibilities of investigators**

1. To take necessary and reasonable medical measures for the subject's safety;
2. To determine whether the serious adverse events are related to the trial itself or the investigational product;
3. To record and follow up the serious adverse events;
4. To timely report to relevant units as required by GCP.

**Responsibilities of contract research organization**

1. To review the completeness, accuracy and authenticity of SAE report of the study site and report to the sponsor;
2. To report to relevant administration authorities within 24 hours after learning of SAEs according to CFDA regulations;
3. To collect and track clinical trial safety data;
4. To summarize and report the safety data of the clinical trials.

### 9.2.4 Follow-up of serious adverse events

Any SAE (including abnormal laboratory tests) that is unresolved at the end of the trial or when subject prematurely withdraws from the trial must continue to be followed-up, until any of following conditions is met:

1. The event is resolved;
2. The event turns stable;
3. The event returns to baseline;
4. The event can be attributed to drugs other than the investigational product or is unassociated with the trial;
5. It's unable to acquire more information (the subject refuses to provide more information, or the subject is still lost to follow-up after best effort).

## Pregnancy

If a female subject or the female partner of a male subject becomes pregnant during the trial, the medication will be discontinued immediately and she should be removed from the trial with the case reported as specified. The investigator should notify Suzhou Zelgen Biopharmaceutical Co., Ltd. within 24 hours after becoming aware of the pregnancy. The pregnancy related events shall be followed up until 30 days after the end of pregnancy. Pregnancy related events include spontaneous abortion or induced abortion, delivery details, birth defects or congenital malformation of newborns, malformation and abnormality in stillbirths, and complications in mothers and newborns. Report, record, follow-up and progression of pregnancy related events shall be handled as SAE.

# DATA MANAGEMENT

## Electronic Case Report Form

Clinical data are remotely input into eCRFs in this trial. The investigators must keep the source files of all subjects, including the original medical records and all other paper data or records. The data input into the eCRF must be traceable to the source files among subject files.

## Establishment and Testing of Database, and Data Input and Modification

- Database design: The data manager designs the database and tests it with analog data or real eCRF data to ensure the accuracy.
- Data input: The sponsor is responsible for training the investigators of all study sites or CRCs in the *eCRF Completion Guide* so as to understand the eCRF content, be familiar with the database structure and function, and understand the problems to be avoided in the input. The investigators or CRCs may contact the data department at any time during the data input, to talk about and solve the problems encountered.
- Data verification: Data verifications during data management include logic verification, manual verification, medical verification, statistical pre-analysis verification and other stages. All data queries are provided in EDC system in the form of electronic queries for the study sites to answer. If the answer meets the requirement, the query will be closed. If the data problems are still unresolved or new queries arise after updating the database based on the solutions of previous data problem, the investigator or CRC shall answer again. The above processes shall be repeated until all data in the database are verified.

## Locking of Database

Statisticians shall compile a blind audit report based on the trial protocol, data review standard and databases. The project manager will hold a data audit meeting with the sponsor, the principal investigator, the statistician and the data manager to review data, and representatives of all parties attending the meeting will sign the data audit resolution. Upon all parties' approval, the data manager will lock the database. The locked data is submitted to the statistician for statistical analysis.

The data may be locked when all the following conditions are met.

1. All data are collected into the database
2. All codes are verified and confirmed
3. All data challenges are resolved (including those raised during data audit)
4. Database has passed the QC
5. Data review is completed
6. Original data verification is completed
7. Review of serious adverse events is completed
8. All investigators sign for completion
9. The analyzable cases are defined and saved in the final analysis database
10. The statistical analysis plan is signed.

# STATISTICAL ANALYSIS

## Sample Size

Approximately 100 subjects will be enrolled during Stage 1, with 50 subjects per group. In accordance with the results of clinical trials published by Momelotinib and the results of the COMFORT-I trial studying Ruxolitinib in the treatment of MF, assuming Jaktinib has a 48% response rate (efficacy indicator: reduction in spleen volume at Week 24 ≥ 35%), and the dropout rate is no more than 10%, then 50 subjects can ensure that the width of 95% confidence interval of the response rate does not exceed ± 15%.

The 100 mg b.i.d. group will be expanded during Stage 2. Assuming the efficacy of Jaktinib for the treatment of MF (efficacy endpoint: ≥ 35% reduction in spleen volume at 24 weeks) is 48% (no less than 23%), with 80% power, 95% confidence interval (two-sided), and a drop-out rate no more than 10%, then the sample size for Stage 2 is 36 subjects.

A total of 136 subjects will be enrolled for this study.

## Study Population

The analysis population of this trial include Full Analysis Set (FAS), Per Protocol Set (PPS), Safety Set (SS), and PK set.

**Full Analysis Set (FAS):**

FAS is used for dropout analysis, equilibrium analysis of basic parameters, and primary efficacy endpoint analysis.

According to the Intention-to-Treat (ITT) principle, the FAS includes all randomized and enrolled subjects who have received at least 1 dose of Jaktinib per protocol. Subjects with the any of the following will be excluded from FAS: 1) missing baseline radiographic assessment; or 2) baseline radiographic assessment not reviewed by IRC. For cases with no PFS and LFS data obtained, the PFS or LFS shall be considered censored, and the censoring time shall be the latest time of confirmed non-PD. In the efficacy analysis, subjects who do not receive the allocated study treatment will be still summarized by the allocated treatment group.

**Per Protocol Set (PPS):**

The PPS is a subset of the FAS, which includes all randomized and enrolled subjects who have received at least 12 weeks of Jaktinib treatment and completed at least 1 efficacy assessment per protocol. Subjects who haven't completed 12 weeks of treatment but have clear medical evidence and disease progression after starting treatment are also included in the PPS. For cases with no PFS and LFS data obtained, the PFS or LFS shall be considered censored, and the censoring time shall be the latest time of confirmed non-PD.

Efficacy is analyzed in the FAS and PPS, with PPS as the primary analysis results. Detailed methods of missing data processing and sensitivity analysis are described in the Statistical Analysis Plan (SAP).

**Safety Set (SS):**

All subjects who signed the informed consent form, and received at least one dose of Jaktinib. In the safety analysis, subjects who do not receive the allocated study treatment will be summarized by the actual treatment. All safety analysis are performed in the SS.

**Pharmacokinetics Analysis Set (PK Set):**

PKAS includes all subjects who received at least one dose of Jaktinib, and had at least one drug concentration sample, collected according to the scheduled PK time points, with no major protocol violations that may significantly affect the PK assessment. The PKAS is used for PK analysis.

Before locking the database, the major protocol violation shall be defined and various sets for analysis shall be finalized by the principal investigator, statisticians and the sponsor together.

## Baseline and Demographic Characteristics

Descriptive statistics are provided for different treatment groups.

## Efficacy Analysis

### 11.4.1 Primary efficacy parameter

All randomized and enrolled subjects (ITT population) will be included in the primary analysis. The efficacy analysis will be carried out using PPS and FAS data, with FAS as the main analysis set.

The primary efficacy endpoint is response rate. The response rate and 95% CI for different dose groups and the overall total are described. Inter-group comparisons will be carried out using the CMH test stratified by study site.

### 11.4.2 Secondary efficacy parameters

Secondary efficacy endpoints are: DoMSR, PFS, LFS, and OS.

The median PFS, LFS, OS, and DoMSR of the two groups and the overall are estimated using the Kaplan-Meier curves.

The HR of Jaktinib efficacy and the corresponding 95% confidence interval (CI) will be estimated using an univariate Cox proportional hazards model, and a survival curve will be plotted.

The HR of each covariate and the corresponding 95% CI will be estimated using a multivariate Cox proportional hazards model. Covariates in Cox regression model include groups (Jaktinib groups at different doses), cause (primary vs. secondary), DIPSS-plus score (intermediate risk vs. high risk), age (18-60 years vs. over 60 years), and JAK2V617F (***mut*** vs. ***wt***). The Cox proportional hazards model will be constructed using a stepwise method. The test is statistically significant if the upper limit of the 95% CI of HR of a certain covariate in the model is less than 1 (*p* < 0.05). A subgroup analysis will be performed for covariates that are statistically significant.

PFS refers to the time from the date of randomization and enrollment to the date of any of the following events: 1) ≥ 25% increase in spleen volume from nadir (including baseline); 2) death from any cause. For subjects who start an MF treatment not described in the protocol before any of the events above, their PFS time will be censored at the date of last confirmed non-PD. Other anti-MF treatments include: a. chemotherapy: such as cladribine, melphalan, busulfan and hydroxyurea; b. radiotherapy to the spleen; c. splenectomy; d. allogeneic hematopoietic stem cell transplantation; e. other JAK inhibitors.

LFS: The time from the date of randomization and enrollment to the date of any of the following events: 1) first bone marrow blasts ≥ 20%; 2) first peripheral blood blasts ≥ 20% and absolute blast count ≥ 1 × 10^9^/L lasting for at least 2 weeks; 3) death from any cause. For subjects who start an MF treatment not described in the protocol before any of the events above, their LFS time will be censored at the date of last confirmed non-PD prior to the database lock. Other anti-MF treatments include: a. chemotherapy: such as cladribine, melphalan, busulfan and hydroxyurea; b. radiotherapy to the spleen; c. splenectomy; d. allogeneic hematopoietic stem cell transplantation; e. other JAK inhibitors.

For subjects who do not progress or transform into leukemia prior to the database lock, their PFS and/or LFS time will be censored at the date of last confirmed non-PD prior to the database lock.

DoMSR refers to the time between the first occurrence of spleen volume reduction of ≥ 35% from baseline to an increase in spleen volume so that the reduction is less than 35% from baseline. For subjects with no DoMSR endpoint event prior to the database lock, their DoMSR will be censored at the date of last confirmed ≥ 35% reduction from baseline prior to the database lock. For subjects who start an MF treatment not described in the protocol before meeting the DoMSR endpoint, their DoMSR will be censored at the date of last confirmed ≥ 35% reduction from baseline. Other anti-MF treatments include: a. chemotherapy: such as cladribine, melphalan, busulfan and hydroxyurea; b. radiotherapy to the spleen; c. splenectomy; d. allogeneic hematopoietic stem cell transplantation; e. other JAK inhibitors.

The anemia response rate and 95% CI for each dose groups and the total will be described. Inter-group comparisons will be carried out using the CMH test stratified by study site.

Survival data within 2 years after treatment discontinuation will be collected. For subjects who are still alive at the end of survival follow-up, their survival time will be censored at the date of last confirmation of their survival.

Data from secondary and exploratory endpoints are collected until the database is locked for the primary endpoint analysis.

Refer to the Statistical Analysis Plan (SAP) for details.

## Safety Analysis

Descriptive summary of all safety parameters is provided for treatment groups. Adverse events will be monitored using NCI-CTCAE V4.03. Treatment-emergent adverse events and laboratory parameters will be summarized by treatment group and CTCAE V4.03 severity grade. After the end of the trial, safety data will be collected from all subjects who received the investigational drug until 28 days after the last dose.

Refer to the Statistical Analysis Plan (SAP) for details.

## PK Analysis

Appropriate descriptive statistics are listed to summarize the plasma concentrations of Jaktinib hydrochloride and its metabolites in each dose group. If necessary, the plasma concentration of Jaktinib hydrochloride and its metabolites may be used in pharmacokinetics or combined pharmacokinetics/pharmacodynamics analysis to further explore Jaktinib hydrochloride’s pharmacokinetics and the relationships between exposure and safety/efficacy. Based on the analysis, a separate analysis plan shall be prepared and the results will be separately reported (not provided in the clinical study report).

## Interim Analysis

An interim analysis is planned for this study to evaluate the safety, efficacy data, and pharmacokinetic properties. The same statistical methods will be used for the interim analysis and the final analysis.

Refer to the Statistical Analysis Plan for details.

# ETHICS

## Ethics Committee

This trial is implemented in accordance with GCP, Declaration of Helsinki, relevant regulations and ethics committee reviews.

The investigator must ensure that this trial is reviewed and approved by a qualified ethics committee which meets the requirements of GCP. Prior to the start of the trial, the investigator must submit the trial protocol, informed consent form, and other necessary materials to the ethics committee for review and approval. The sponsor may only provide the investigational drugs after receiving the approval from the ethics committee. The ethics committee must be notified of any subsequent supplementary of the protocol and serious adverse events that may affect the safety and continued participation of subjects. The investigator is responsible for reporting the progress of the trial to the ethics committee. In addition, the investigator must promptly provide the sponsor with a copy of records of communication with the ethics committee. When reviewing and approving the trial protocol, the ethics committee must verify the protocol title and protocol number, and note the reviewed documents and the date of the review. During the trial, according to the regulations, written approval from the relevant authorities must be obtained for any revisions to the trial protocol or informed consent form.

## Informed Consent

The investigator must provide information regarding this trial both in oral and written form. Subjects, guardians and legal representatives (if necessary) have the right to know all details regarding this trial.

The informed consent form (along with the trial protocol) must be submitted to the ethics committee for review and approval. If necessary, the investigator is responsible for explaining the contents of the informed consent form in a manner and wording that subjects can understand. Subjects and representatives must have sufficient time to read the informed consent form prior to signing it.

The final informed consent form should include the following: purpose of the trial, process and duration of the trial, examinations and procedures, potential benefits and risks, the different intervention groups that subjects may be assigned to, the treatment and corresponding compensation available to subjects in the event of harm to subjects associated with the trial, and the confidentiality nature of subjects' personal information.

The informed consent form must be signed and dated by the subject (or the subject's statutory guardian). The investigator should also sign and date the informed consent form. The form should also be signed by an independent witness who can prove that the subject has agreed to participate in the trial. One copy of the informed consent form should be kept by the investigator and one by the subject. If important new information is discovered regarding the investigational drug, the informed consent form must be revised and submitted to the ethics committee for approval, after which informed consent must be obtained again from the subject.

## Subject Confidentiality

The investigator is responsible for maintaining subjects' confidentiality. Only capital letter, number and/or codes can be used on the case report form or other documents to identify the subjects. Names must not be used. The investigator must safely keep the subjects enrollment log documented with subjects' codes, name, and home address. The investigator must maintain strict confidentiality of documents that show the identity of subjects.

# TRIAL MANAGEMENT

## Independent Data Monitoring Committee

An independent data monitoring committee is established to ensure the trial can be conducted safely. Safety review meeting is held according to the independent data monitoring committee (IDMC) charter. Subject recruitment will continue during the scheduled meeting of the data monitoring committee. After assessing the recommendations from the data monitoring committee, the sponsor may decide to terminate the trial, modify or discontinue the recruitment of a certain dose group based on safety and efficacy results.

## Protocol Revision

All revisions must be kept as protocol supplementary documents. All revisions must be approved by the ethics committee.

## Training

Based on GCP principles, the monitor should have the qualifications recognized by the sponsor. Prior to the start of the trial, the directors of each study site should train investigators to familiarize themselves with the contents of the clinical trial protocol, master the GCP principles, standardize recording methods and judgment standards, and strictly follow the protocol.

## Standard Operation

The sponsor must manufacture the investigational drugs in workshops which meet the requirement of GMP, and undergo strict quality inspection, as required by the GCP and Drug Registration Regulations. Laboratory tests are performed by each study site according to SOP. The test methods and quality control of each study site must be standardized. The clinical laboratory of each study site must undergo quality control and receive the certificate of conformance for quality evaluation from national center for clinical laboratory.

## Monitoring

The clinical trial monitor is the primary contact person between the sponsor and the investigator. The monitor must follow the GCP and SOP, visit and monitor the trial site on a regular basis or according to actual conditions, supervise the progress of the trial, check and verify the recording and reporting of all data, ensure the eCRF is accurate, complete, and consistent with source data, and ensure the trial is being carried out in accordance with the protocol. The investigator should actively cooperate with the monitors. Responsibilities of the monitor include the following:

1. Prior to the start of the trial, confirm that the trial site has the appropriate conditions including staffing and training, that the laboratory is fully equipped and well-run, has various test conditions related to the trial, and has enough subjects to enroll, and that trial personnel is familiar with the protocol requirements;
2. During the trial, monitor how the investigator is implementing the trial, ensure informed consent form is obtained from all subjects prior to the trial, understand the enrollment rate and the progress of the trial, and confirm that the enrolled subjects meet the inclusion criteria;
3. Verify the recording and reporting of all data to ensure the accuracy and integrity of the data, and ensure that all report forms are correct and consistent with source data. Any errors or omissions has been corrected or noted, and signed and dated by the investigator. Dosage changes, treatment changes, concomitant medications, intercurrent diseases, loss to follow-up, and inspection omissions for each subject should be verified and recorded. Verify that the reasons for subjects' withdrawal and loss to follow-up are documented;
4. Ensure all adverse events are recorded, and serious adverse events are recorded and reported within the required time frame. Verify the provision, storage, dispensing, and return of the investigational drug according to regulations, and corresponding records are available;
5. Assist the investigator in notifications and applications when necessary, and report trial data and results to the sponsor;
6. Clearly record follow-up visits, tests, and examinations that the investigator failed, and whether errors and omissions are corrected;
7. Complete a written monitoring report after each visit. The report should include the date, time, name of the monitor and any findings.

## Quality Control and Quality Assurance

1. A standard operating procedure (SOP) is established for the entire trial.
2. Qualifications of trial sites: The clinical trial sites should have ideal clinical conditions as required by the NMPA.
3. Qualifications of the trial personnel: Trial personnel should be physicians with clinical trial training, who should work under the supervision of senior professionals.
4. Quality control of the laboratory: The laboratory should establish SOPs and quality control procedures for experimental observations.
5. Trial monitoring: Trained monitors need to regularly monitor the progress of the trial. The monitor should ensure the clinical facilities of the study site meet the requirements, ensure trial personnel follows the trial protocol and accurately records trial results. During every monitoring, the monitor should review the eCRF and source documents, and check the medication storage conditions and drug usage records.
6. Trial auditing: According to the requirements of GCP, the study site must be audited by a auditor designated by the sponsor. The auditor conducts a systematic check for relevant clinical trial activities and documents, to assess whether the trial is in accordance with trial protocol, SOPs, and relevant regulations, and whether the trial data is recorded promptly, truthfully, accurately, and completely. The auditing should be performed by personnel not directly involved in the trial.

## Audit and Inspection

The sponsor's quality assurance department should audit the clinical trial institution. Audit includes: the provision of the medication, the required documents and the recording of informed consent process, as well as the consistency between case report forms and source documents. The content and scope of the audit may be increased depending on the situation. Upon reasonable notification, the investigator should allow the auditor designated by Suzhou Zelgen Biopharmaceuticals Co., Ltd. to audit the trial, and allow the inspections conducted by regulatory authority. The main purpose of audits and inspections are to verify that the rights and health of participating subjects are protected, that the signing of the informed consent forms and the trial are carried out properly, and the drug evaluation data is processed and reported in accordance with the SOPs of scheduling, protocol, facilities, and ethics, ICH/GCP, and applicable regulations. The investigator should ensure the trial documents, source data, and source documents are accessible.

## Trial Data Archiving

According to GCP, the investigator must keep the clinical trial data for 5 years after the termination of the trial. The sponsor must keep the clinical trial data for 5 years after the product is approved for manufacturing and marketing.

# EXPECTED PROGRESS AND COMPLETION DATE OF THE CLINICAL TRIAL

Estimated to be Jan 2018 to Jan 2021.

# FOLLOW-UP AFTER THE COMPLETION OF THE TRIAL AND MEDICAL MEASURES

The investigator should follow up the AEs and SAEs that occur during the trial until the AEs and SAEs resolve. If an AE or SAE persists, continue follow-up until one of the following occurs (whichever occurs first): 1) adverse reaction resolves; 2) safety follow-up (28 days after the last dose of investigational drug); 3) death; 4) study termination; 5) start of another anti-MF treatment.

Any AE/SAE (including abnormal laboratory tests) that is unresolved after the termination of the trial or after the subject prematurely withdraws from the trial must continue to be followed-up. Refer to section "9.2.4 Follow-up of serious adverse events" for details. After the termination of the trial, the investigator should provide the necessary and reasonable medical measures to ensure the safety and rights of the subjects.

# RESPONSIBILITIES OF EACH PARTY

## Responsibilities of the Investigator

The responsibilities of the investigator primarily include but are not limited to the following:

1. The investigator should come up with the trial protocol together with the sponsor, sign and submit the protocol to the ethics committee. The trial only can be carried out upon approval.
2. The investigator must carefully read and understand the contents of the protocol, and carry out the trial in strict accordance with the protocol.
3. The investigator should be familiar with the characteristics, function, efficacy, and safety of the investigational drug (including relevant preclinical information), and be aware of any new information related to the investigational drug discovered during the trial.
4. The investigator should conduct the study at a medical institution that has optimal medical facilities, laboratory equipment, and staffing. To ensure the safety of the subjects, the institution must be able to deal with emergencies. Laboratory test results should be accurate and reliable.
5. The investigator should obtain the approval of the medical institution or competent authority, and ensure that the trial can be completed within the time frame specified in the protocol. The investigator should explain the relevant trial information, requirements and responsibilities to all participating personnel, and ensure a sufficient number of eligible subjects enrolled into the clinical trial.
6. The investigator should explain to subjects the details regarding the trial approved by the ethics committee, and obtain informed consent forms.
7. The investigator is responsible for making medical decisions related to the clinical trial, ensuring subjects are adequately treated for any adverse events that occur during the trial.
8. The investigator is duty-bound to take necessary measures to ensure the subject's safety, and to document any measures taken. If a serious adverse events occurs during the trial, the investigator should immediately provide appropriate treatment, and report the event to the Food and Drug Administration, the department of public health administration, the sponsor, and the ethics committee. The investigator must sign and date the report.
9. The investigator should ensure the data inputted into the medical records and eCRF is true, accurate, complete, timely, and legal.
10. To ensure the quality of the clinical trial, the investigator should accept the monitoring, and audit of auditors and monitors designated by the sponsor and the audit and inspection of drug regulatory authorities.
11. The investigator should consult with the sponsor about the cost of the clinical trial, which should be included in the contract. The investigator must not charge subject for the investigational drug.
12. After the clinical trial is completed, the investigator must write a summary report, sign and date, and submit it to the sponsor.

## Responsibilities of the Sponsor

The responsibilities of the sponsor primarily include but are not limited to the following:

1. The sponsor should obtain approval from the NMPA.
2. The sponsor is responsible for initiating and applying for a clinical trial, as well as providing the funding for the trial.
3. The sponsor should provide the investigator's brochure to specify the chemical, pharmaceutical, toxicological, pharmacological, and clinical (including previous or ongoing trials) information and data of the investigational drug.
4. The sponsor should design the clinical trial protocol together with the investigator. Sign the trial protocol and contract agreed by both parties.
5. The sponsor should provide the investigator with the investigational drug and control drug that are easily identified, accurately coded and affixed with special labels, and guarantee the quality. The investigational drugs should be packaged and stored according to protocol requirements. The sponsor should establish the system for managing and recording the use of the investigational drug.
6. The sponsor should designate a qualified monitor who is accepted by the investigator.
7. The sponsor should establish quality control and quality assurance systems for the clinical trial, and organize clinical trial auditing to ensure the quality.
8. The sponsor, together with the investigator, should promptly deal with the SAEs that occur during the trial, take the necessary measures to the ensure subjects' safety and rights, and immediately report the SAEs to the drug regulatory authorities and the department of public health administration.
9. The sponsor is responsible for submitting the final summary report to the NMPA.
10. The sponsor should provide insurance for all participating subjects, and cover the cost of treatment and the corresponding financial compensation for subjects who suffer from trial-related damage or death. The sponsor should provide the investigator with legal and economic guarantees, except for medical accident.

# CONFIDENTIALITY AND PUBLICATION OF TRIAL RESULTS

All information regarding this trial (not limited to the following: protocol, and investigator's brochure) is confidential. The investigator must realize the scientific or medical information derived from this trial may have commercial value for the sponsor. The investigator should keep all information and data of this trial confidential. The investigator must first obtain the written consent of the sponsor before publishing any information or results related to this trial. In order to protect the sponsor's rights, the sponsor may ask the investigator not to publish any information about the trial before the investigational drug is approved for marketing.

The sponsor has the right to publish information or data related to the trial, or to report it to the drug regulatory authorities. The sponsor must obtain the consent of the investigator if the name of the investigator is to appear in any published content or advertisements.

# REGULATORY AGENCY

## Sponsor

| Name: | Suzhou Zelgen Biopharmaceuticals Co., Ltd. |
| --- | --- |
| Project Leader: | Wu Liqing |
| Address: | No. 209, Chenfeng Road, KunShan, Jiangsu |
| Telephone: | 0512-57309965 |

## Trial Site

### 18.2.1 Lead unit

| Site Name: | The First Affiliated Hospital, Zhejiang University |
| --- | --- |
| Principal Investigator: | Professor Jin Jie |
| Address: | No. 79, Qingchun Road, Hangzhou, Zhejiang |
| Telephone: | 0571-87236896 |

### 18.2.2 Participating sites

Around 25 study sites across the country.

# REFERENCES

- - - 1. Prassopoulos P, Daskalogiannaki M, Raissaki M, Hatjidakis A, Gourtsoyiannis N. Determination of normal splenic volume on computed tomography in relation to age, gender and body habitus.Eur Radiol. 1997; 7(2):246-8.
      2. Muggli D, Müller MA, Karlo C, Fornaro J, Marincek B, Frauenfelder T. A simple method to approximate liver size on cross-sectional images using living liver models. Clinical Radiology. 2009 Jul; 64 (7): 682–9.
      3. CFDA. Guidelines for Pharmacokinetic Studies of Pharmaceuticals. March 2005.
      4. CFDA. Good Clinical Practice. 2003.
      5. CFDA. Drug Registration Regulations. 2007.
      6. Verstovsek S, Mesa RA, Gotlib J, et al. A double-blind, placebo-controlled trial of ruxolitinib for myelofibrosis [J]. N Eng J Med 2012, 366 (9): 799–807.
      7. Harrison C, Kiladjian JJ, Al-Ali HK, et al. JAK inhibition with ruxolitinib versus best available therapy for myelofibrosis[J].N Eng J Med 2012;366(9):787-798.
      8. Verstovsek S, Mesa RA, Gotlib J, et al. Consistent benefit of ruxolitinib over placebo in spleen volume reduction and symptom improvement across subgroups and overall survival advantage: results from COMFORT-I. Blood,2011;118:abstract 278
      9. Harrison C, Kiladjian JJ, Gisslinger H,et al. Ruxolitinib provides reductions in splenomegaly across subgroups: an analysis of spleen response in the COMFORT-II study.Blood 2011;118:abstract 279
      10. JAKAVIR the first medication to receive Health Canada approval to treat patients with myelofibrosis [News release].Canadian Newswire Web site. Available from: http://www.newswire.ca/en/story/1003655/-pr-jakavi-the-first-medicationto-receive-health-canada-approval-totreat-patients-with-myelofibrosis 2012. [Accessed 28 August 2012]
      11. Novartis drug Jakavir first medication to receive European Commission approval to treat patients with myelofibrosis [News release]. Novartis Web site. Available from: http://www.novartis.com/newsroom/media-releases/en/2012/1636508.shtm
      12. Verstovsek S, Kantar Jian H, Mesa Ramjet al. Safety and efficacy of INCB018424, a JAK1 and JAK2 inhibitor, in myelofibrosis[J]. N Engl J Med 2010; 363:1117

# Appendix I. European Consensus on Grading of Myelofibrosis (MF)

| Grade | Description |
| --- | --- |
| MF-0 | Scattered linear reticulin with no intersections, corresponding to normal bone marrow. |
| MF-1 | Loose network of reticulin with many intersections, especially in perivascular areas. |
| MF-2 | Diffuse and dense increase in reticulin with extensive intersections, occasionally with only focal bundles of collagen and/or focal osteosclerosis. |
| MF-3 | Diffuse and dense increase in reticulin with extensive intersections with coarse bundles of collagen, often associated with significant osteosclerosis. |

# Appendix II. Diagnostic Criteria for PMF (WHO 2016)

| **Major Criteria** | **Minor Criteria (with confirmation from two consecutive lab tests)** |
| --- | --- |
| Megakaryocyte hyperplasia and atypia often accompanied by either reticular and/or grade 2 or 3 collagen fibrosis, or without significant reticulin fibrosis (≤ MF-1). Megakaryocyte changes are often accompanied by increased granulocytic proliferation and often decreased erythropoiesis | Anemia, not attributed to a comorbid condition |
| Not meeting WHO criteria for PV, CML (BCR-ABL+), myelodysplastic syndromes (with granulocytic and erythroid dysplasia), or other myeloid neoplasms. | White blood cell count ≥ 11 × 10^9^/L |
| Presence of JAK2 V617F, CALR, or MPL mutation. Or in the absence of these mutations, presence of another clonal marker (such as ASXL1, EZH2, TET2, IDH1/IDH2, SRSF2, and SF3B1), or absence of evidence for reactive BM fibrosis. | Palpable splenomegaly |
|  | Increased LDH level |
|  | Leukoerythroblastosis |

Note: Diagnosis requires meeting all three major criteria and at least one minor criterion.

# Appendix III. Diagnostic Criteria for Post-PV-MR and Post-ET-MF (IWG-MRT)

|  | | | **POST-PV MF** | |  | **POST-ET MF** | |
| --- | --- | --- | --- | --- | --- | --- | --- |
| **Item** | | **Criteria** | **Major Criteria** | **Minor Criteria** |  | **Major Criteria** | **Minor Criteria** |
| Medical history | | Previous diagnosis of PV (WHO criteria) | ◎ |  |  |  |  |
|  |  | Previous diagnosis of ET (WHO criteria) |  |  |  | ◎ |  |
| Clinical examination | | Splenomegaly |  | ◎ |  |  | ◎ |
|  |  | Development of at least 1 of the following signs or symptoms: (1) > 10% weight lost in the past 6 months (2) night sweats (3) unexplained fever |  | ◎ |  |  | ◎ |
| Bone marrow biopsy | | Bone marrow fibrosis grade 2-3 | ◎ |  |  | ◎ |  |
| Other lab results | Routine blood test | Anemia |  | ◎ |  |  | ◎ |
|  | Blood biochemical test | Increased LDH level |  |  |  |  | ◎ |
|  | Peripheral blood smear | Leukoerythroblastosis |  | ◎ |  |  | ◎ |
| Note: Diagnosis for Post-PV MF and Post-ET MF requires meeting at least 2 major criteria and 2 minor criteria. | | | | | | | |

# Appendix IV. Prognostic Scoring Systems for MF

| **Prognostic Factors** | **IPSS^6^** | **DIPSS** | **DIPSS-Plus^5^** |
| --- | --- | --- | --- |
| Age > 65 years | 1 | 1 |  |
| Presence of constitutional symptoms^7^ | 1 | 1 |  |
| HGB < 100g/L^8^ | 1 | 2 |  |
| WBC > 25 × 10^9^/L^9^ | 1 | 1 |  |
| Peripheral blood blasts ≥ 1%^10^ | 1 | 1 |  |
| PLT < 100 × 10^9^/L^11^ | -- | -- | 1 |
| Transfusion dependency^12^ | -- | -- | 1 |
| Unfavorable karyotype^1^ | -- | -- | 1 |
| DIPSS intermediate-1 risk^2^ | -- | -- | 1 |
| DIPSS intermediate-2 risk^3^ | -- | -- | 2 |
| DIPSS high risk^4^ | -- | -- | 3 |

Note: 1. Unfavorable karyotype includes complex karyotype or single or two abnormalities including +8, −7/7q-, i(17q), −5/5q-, 12p-, inv(3) or 11q23 rearrangement; history of “unfavorable karyotype”;

2. DIPSS intermediate-1 risk: score = 1 or 2;

3. DIPSS intermediate-2 risk: score = 3 or 4;

4. DIPSS high risk: score = 5 or 6;

5. DIPSS-Plus groups: low risk (score = 0), intermediate-1 risk (score = 1), intermediate-2 risk (score = 2 or 3), high risk (score = 4-6)

6. IPSS groups: low risk (score = 0), intermediate-1 risk (score = 1), intermediate-2 risk (score = 2), high risk (score ≥ 3);

7. Constitutional symptoms: History of “weight loss, unexplained fever, and night sweats”;

8. HGB < 100g/L: Meets any one of the following criteria: 1) History of RBC transfusion (e.g., MF-related anemia requiring transfusion); or 2) Two or more consecutive tests of HGB < 100 g/L (MF-related anemia); or 3) baseline HGB < 100 g/L;

9. WBC > 25 × 10^9^/L: History of “WBC > 25 × 10^9^/L”;

10. Peripheral blood blasts ≥ 1%: History of “peripheral blood blasts ≥ 1%”;

11. PLT < 100 × 10^9^/L: Meets any one of the following criteria: 1) history of platelet transfusion (e.g., MF-related platelet decrease requiring transfusion); or 2) two or more consecutive tests of PLT < 100 × 10^9^/L (MF-related platelet decrease); or 3) baseline PLT < 100 × 10^9^/L;

12. Transfusion dependence: History of “transfusion dependence (e.g., MF-related anemia requiring transfusion)”;

# Appendix V. MPN-SAF TSS Assessment

| **Symptom** | **Score** |
| --- | --- |
| Fatigue: please rate your fatigue (weakness or debility) by circling the one number that best describes your WORST level of fatigue during the past 24 hours. | (None) 0 1 2 3 4 5 6 7 8 9 10 (Worst Imaginable) |
| Circle the one number that best describes how severe each of the following symptoms was during the past week | |
| Early satiety | (None) 0 1 2 3 4 5 6 7 8 9 10 (Worst Imaginable) |
| Abdominal discomfort | (None) 0 1 2 3 4 5 6 7 8 9 10 (Worst Imaginable) |
| Inactivity | (None) 0 1 2 3 4 5 6 7 8 9 10 (Worst Imaginable) |
| Problems with concentration | (None) 0 1 2 3 4 5 6 7 8 9 10 (Worst Imaginable) |
| Night sweats | (None) 0 1 2 3 4 5 6 7 8 9 10 (Worst Imaginable) |
| Pyrexia | (None) 0 1 2 3 4 5 6 7 8 9 10 (Worst Imaginable) |
| Weight loss (in the past 6 months) | (None) 0 1 2 3 4 5 6 7 8 9 10 (Worst Imaginable) |
| Pruritus | (None) 0 1 2 3 4 5 6 7 8 9 10 (Worst Imaginable) |
| Bone pain (diffuse, not joint pain or arthritis) | (None) 0 1 2 3 4 5 6 7 8 9 10 (Worst Imaginable) |

# Appendix VI. Response Criteria for Myelofibrosis (IWG-MRT)

| Complete response (CR) | Meeting all of the following: 1) Bone marrow: age-adjusted normocellularity; < 5% blasts; ≤ Grade 1 MF (European classification); 2) Peripheral blood: HGB ≥ 100 g/L, PLT ≥ 100 x 10^9^/L, ANC ≥ 1 x 10^9^/L, and all of the above < ULN; < 2% immature myeloid cells; 3) Resolution of clinical signs and symptoms (hepatauxe and splenomegaly not palpable); no evidence of extramedullary hematopoiesis. |
| --- | --- |
| Partial response (PR) | Meeting one of the following: 1) Peripheral blood: HGB ≥ 100 g/L, PLT ≥ 100 × 10^9^/L, ANC ≥ 1 × 10^9^/L, and all of the above < ULN; < 2% immature myeloid cells; resolution of clinical signs and symptoms (hepatauxe and splenomegaly not palpable); no evidence of extramedullary hematopoiesis. 2) Bone marrow: age-adjusted normocellularity; < 5% blasts; ≤ Grade 1 MF; peripheral blood: HGB (85- < 100 g/L), PLT (50- < 100) x 10^9^/L, ANC ≥ 1 x 10^9^/L, and all of the above < ULN; < 2% immature myeloid cells; resolution of clinical signs and symptoms (hepatauxe and splenomegaly not palpable); no evidence of extramedullary hematopoiesis. |
| Clinical improvement (CI) | The achievement of anemia, spleen or symptom response without progressive disease or increase in severity of anemia, thrombocytopenia, or neutropenia.  Anemia response: Transfusion-independent patients have a ≥ 20 g/L increase in HGB level; transfusion-dependent patients becoming transfusion-independent (no RBC transfusions for at least 12 consecutive weeks and HGB ≥ 85 g/L).  Spleen response: 1) A baseline splenomegaly that was palpable at 5-10 cm below the LCM becomes not palpable; 2) A baseline splenomegaly that was palpable at > 10 cm below the LCM decreases by ≥ 50%; 3) A baseline splenomegaly that was palpable at < 5 cm below the LCM is not eligible for spleen response; 4) A spleen response requires confirmation by MRI or CT showing ≥ 35% spleen volume reduction.  Symptom response: A ≥ 50% reduction in the MPN-SAF TSS. |
| Progressive disease (PD) | Meeting one of the following: 1) Appearance of a new splenomegaly that is palpable < 5 cm below the LCM 2) A ≥ 100% increase in palpable distance, below LCM, for baseline splenomegaly of 5-10 cm; 3) A > 50% increase in palpable distance, below LCM, for baseline splenomegaly of > 10 cm; 4) Leukemic transformation confirmed by a bone marrow blast count of ≥ 20%; 5) A peripheral blood blast content of ≥ 20% associated with an absolute blast count of ≥ 1 × 10^9^/L that lasts for at least 2 weeks. |
| Stable disease (SD) | Belonging to none of the above listed response categories |
| Relapse | Meeting one of the following: 1) No longer meeting criteria for at least CI after achieving CR, PR, or CI; 2) Loss of anemia response persisting for at least 1 month; 3) Loss of spleen response persisting for at least 1 month |
| Cytogenetic remission | At least 10 metaphases must be analyzed for cytogenetic response evaluation, and confirmation by repeated testing within 6-month window is required. 1) Complete response: eradication of a pre-existing abnormality 2) Partial response: a ≥ 50% reduction in abnormal metaphases (partial response applies only to patients with at least 10 abnormal metaphases at baseline). |
| Molecular remission | Molecular remission evaluation must be analyzed using peripheral blood granulocytes and requires confirmation by repeated testing within 6-month window. 1) Complete response: eradication of a pre-existing abnormality; 2) Partial response: a ≥ 50% decrease in allele burden (partial response applies only to patients with at least 20% mutant allele burden at baseline). |
| Cytogenetic/molecular relapse | Re-emergence of a pre-existing cytogenetic or molecular abnormality that is confirmed by repeated testing. |

# Appendix VII. Strong CYP3A4 Inducers/Inhibitors/CYP 2B6 Substrates

| **Strong CYP3A4 inhibitors** | **Strong CYP3A4 inducers** | **CYP 2B6 substrates** |
| --- | --- | --- |
| - Clarithromycin, erythromycin, troleandomycin, telithromycin, - conivaptan, gemfibrozil - indinavir, lopinavir, nelfinavir, ritonavir, saquinavir, tipranavir, telaprevir, elvitegravir, boceprevir - itraconazole, ketoconazole, posaconazole, voriconazole, nefazodone, - mibefradil, and grape fruit juice | Rifampin, St. John's wort, carbamazepine, phenytoin, and avasimibe | Ketamine, efavirenz, and nicotine |

# Appendix VIII. QTcB Formula

QTcB = QT/(RR^0.5)

# Appendix IX. Glucocorticoids Dose Equivalence Conversion

0.75 mg dexamethasone = 5 mg prednisone = 4 mg methylprednisolone = 20 mg hydrocortisone
