# Supplementary material for: Safety and efficacy of jaktinib in the treatment of Janus kinase inhibitor‐naïve patients with myelofibrosis: Results of a phase II trial
Source: Am J Hematol. 2022 Oct 4;97(12):1510–9. doi: 10.1002/ajh.26709 (PMC10092883; doi:10.1002/ajh.26709)

**Supplementary 1**

Trial protocol

**Supplementary 2**

Table S1. Dose adjustment

| Platelet count | ▼Dose Group▼ | |
| --- | --- | --- |
|  | 100 mg (2#) BID | 200 mg (4#) QD |
|  | ▼Dose adjustment▼ | |
| ≥ 100 × 10^9^/L | Original dose or increased dose | Original dose or increased dose |
| 75 – < 100 × 10^9^/L | 150 mg (3#) QD | 150 mg (3#) QD |
| 50 – < 75 × 10^9^/L | 100 mg (2#) QD | 100 mg (2#) QD |
| < 50 × 10^9^/L | Discontinue the drug | Discontinue the drug |
| Neutrophil count | ▼Dose Group▼ | |
|  | 100 mg (2#) BID | 200 mg (4#) QD |
|  | ▼Dose adjustment▼ | |
| ≥ 1.5 × 10^9^/L | Original dose | Original dose |
| 1.0 – <1.5 × 10^9^/L | 150 mg (3#) QD | 150 mg (3#) QD |
| 0.5 – <1.0 × 10^9^/L | 100 mg (2#) QD | 100 mg (2#) QD |
| < 0.5 × 10^9^/L | Discontinue the drug | Discontinue the drug |

**Supplementary 3**

Table S2. Data Set

| **Set** | **100 mg BID** | **200 mg QD** | **Total** |
| --- | --- | --- | --- |
| Successfully enrolled subjects | 66 | 52 | 118 |
| Intention-to-treat (ITT) | 66 (100%) | 52 (100%) | 118 (100%) |
| Per-protocol analysis (PPS) | 65 (98.5%) | 48 (92.3%) | 113 (95.8%) |
| Pharmacokinetic analysis (PKAS) | 12 (18.2%) | 11 (21.2%) | 23 (19.5%) |

**Supplementary 4**

Fig. S1. Subgroup analysis on the primary end point - Spleen volume decreased ≥35% from baseline at week 24


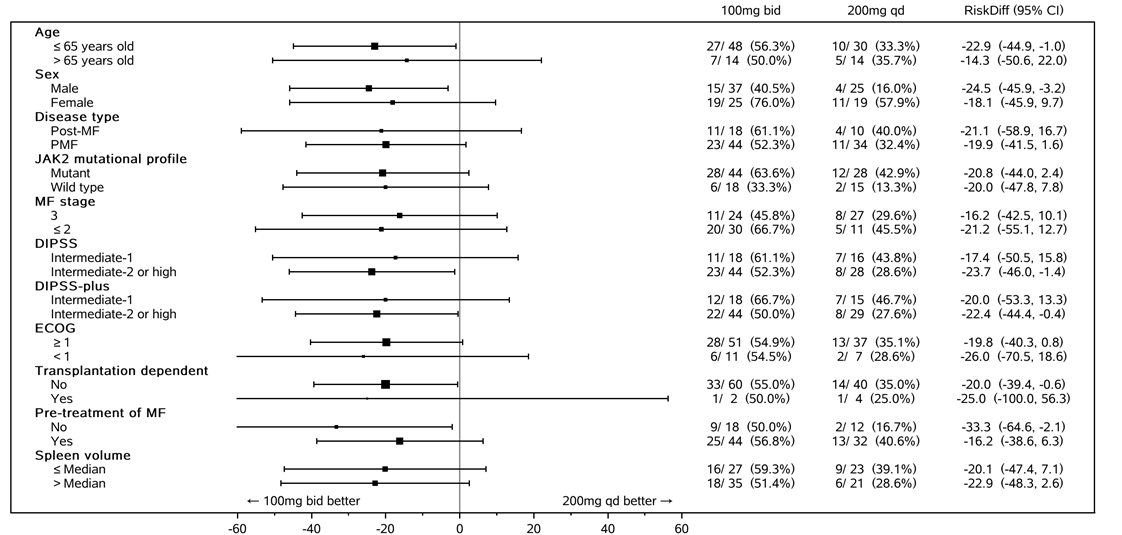


**Supplementary 5**

Table S3. Anemia improvement

| **Parameter** | **100 mg BID**  **(N = 66)** | **200 mg QD**  **(N = 52)** | **Total**  **(N = 118)** |
| --- | --- | --- | --- |
| Number of patients who converted to transfusion-independent after treatment, n | 2 - 0 | 4 - 2 | 6 - 2 |
| Number of patients with HGB ≤100 g/L and transfusion independent at baseline, n | 30 | 29 | 59 |
| Experienced HGB increase of ≥ 20 g/L after treatment, n (%, 95% CI) | 13  (43.3, 25.5 – 62.6) | 8  (27.6, 12.7 – 47.2) | 21  (35.6, 23.6 – 49.1) |
| Number of patients who needed RBC transfusion at baseline*, n | 2 | 5 | 7 |
| Transfusions reduced ≥ 50% after treatment#, n (%, 95% CI) | 1  (50.0, 1.3 – 98.7) | 4  (80.0, 28.4 – 99.5) | 5  (71.4, 29.0 – 96.3) |

*Number of patients who needed RBC transfusion at baseline: Defined as patients who received red blood cell transfusions within 30 days prior to study drug administration.

#Transfusions reduced ≥ 50% after treatment: Defined as the average frequency of blood transfusions per month after jaktinib treatment compared with the frequency of blood transfusions within 30 days prior to the first dose.

**Supplementary 6**

Fig. S2. Change in MPN-SAF TSS.

Panel A shows the change in Total Symptom Score (TSS) from baseline in individual patients at week 24. Panel B shows the proportion of patients whose MPN-SAF TSS decreased ≥50% from baseline over time. I bars denote 95% confidence interval. Panel C shows the mean absolute and percent changes in TSS from baseline. I bars denote standard errors. A、B and C only patients with evaluable results at the time point are included.

**
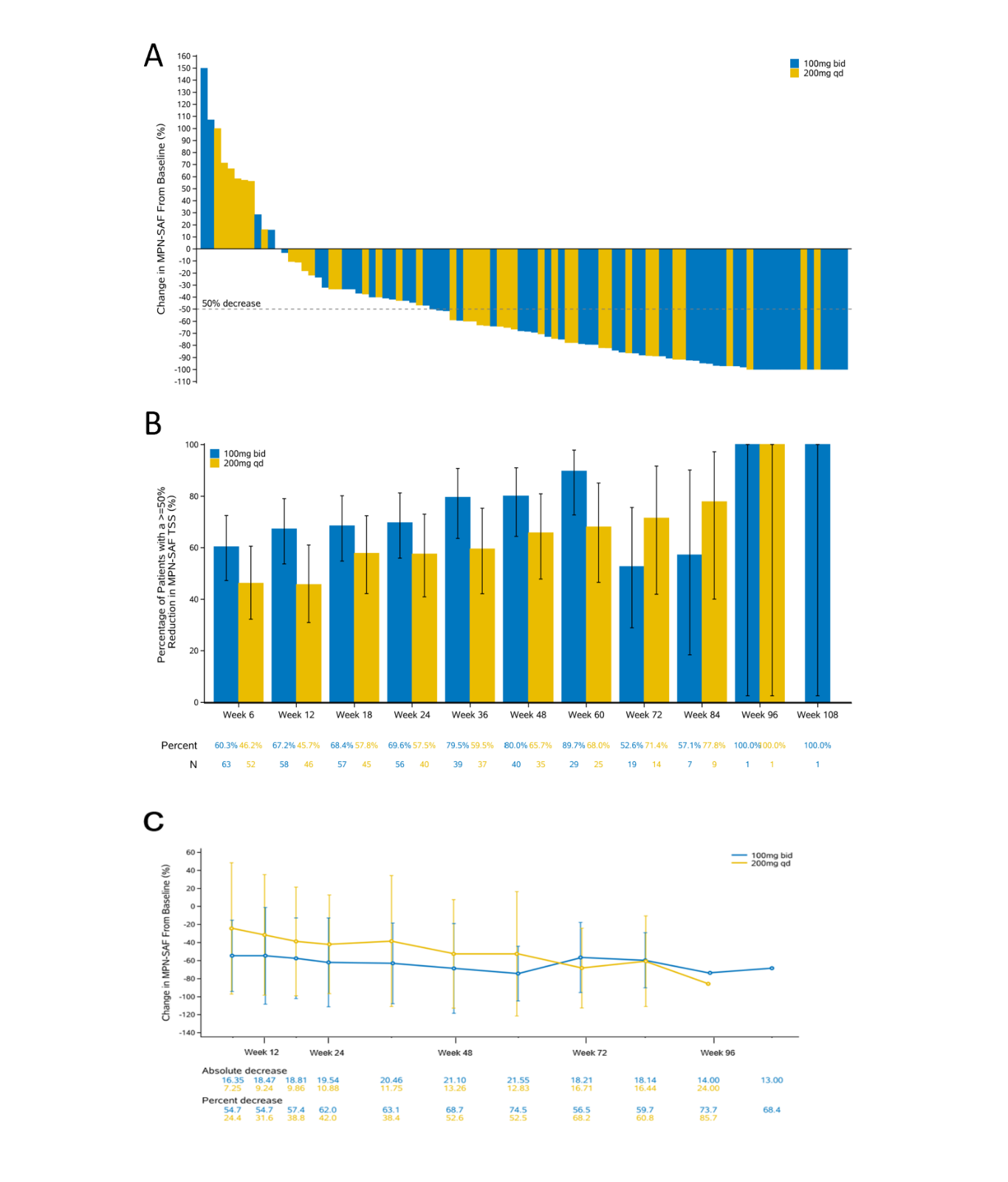
**

**Supplementary 7**

Fig. S3. Survival curves of OS


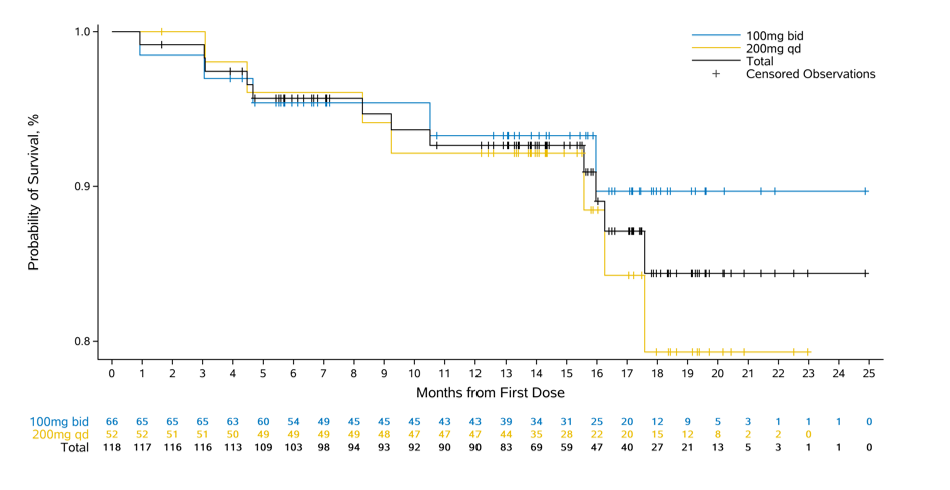

Supplement: Supplementary file 2 — Appendix S2. Supporting Information. [file AJH-97-1510-s001.docx]
